# Pyrene-Functionalized *o*‑COSAN Noncovalently Anchored onto Graphene Oxide for Advanced Photocatalytic Alcohol Oxidation

**DOI:** 10.1021/acsomega.6c07608

**Published:** 2026-09-11

**Authors:** Syrine Affès, Nabila Mimouni, Isabel Guerrero, Rosario Núñez, Ahlem Kabadou, Clara Viñas, Francesc Teixidor, Isabel Romero

**Affiliations:** † Departament de Química i Serveis Tècnics de Recerca, 16738 Universitat de Girona, C/M. Aurèlia Capmany, 69, E-17003 Girona, Spain; ‡ Institut de Ciencia de Materials de Barcelona (ICMAB-CSIC), Carrer dels Til·lers, 08193 Cerdanyola del Vallès,, Spain; § Laboratoire des Sciences des Matériaux et d’Environnement, Faculté des Sciences, Université de Sfax, Sfax 3029, Tunisie

## Abstract

We report the synthesis and characterization of new pyrene-functionalized cobaltabis­(dicarbollide) anions [o-COSAN]^‑^ and their first successful immobilization onto graphene oxide (GO). The complex Na­[3,3′-Co­(8-O­(CH_2_CH_2_O)_2_C­(O)-(CH_2_–pyrene)­(1,2-C_2_B_9_H_10_)­(1′,2′-C_2_B_9_H_11_], denoted as Na­[**3**], was obtained via a ring-opening reaction of a cyclic oxonium derivative with pyreneacetic acid. Subsequent cation exchange yielded the tetramethylammonium salt, [N­(CH_3_)_4_]­[3,3′-Co­(8-O­(CH_2_CH_2_O)_2_C­(O)­(CH_2_–pyrene-1,2-C_2_B_9_H_10_)­(1,2-C_2_B_9_H_11_)], [N­(CH_3_)_4_]­[**3**]. Electrochemical analysis revealed irreversible oxidation peaks for the pyrene moiety alongside well-defined Co­(IV)/Co­(III) and Co­(III)/Co­(II) redox couples. Noncovalent anchoring of [Na]­[**3**] onto GO produced the hybrid material GO@**3**, which was thoroughly characterized by TEM, SEM, XPS, UV–vis, and DPV. GO@**3** exhibited the narrowest optical and electrochemical bandgaps among the studied species. All complexes demonstrated exceptional catalytic efficiency and selectivity for the UVA-driven photooxidation of alcohols in water. Notably, homogeneous Na­[**3**] achieved high conversions at a remarkably low loading of 0.01 mol %. While the insoluble [N­(CH_3_)_4_]­[**3**], acted as a robust heterogeneous catalyst, the hybrid **GO@3** surpassed the activity of the homogeneous system, showing enhanced performance in shorter reaction times. This enhancement is attributed to the synergistic role of GO in facilitating electron transfer and suppressing charge recombination. GO@**3** was easily recovered by filtration and reused over six cycles without loss of activity, reaching turnover numbers (TON) exceeding 45,000.

## Graphical abstract

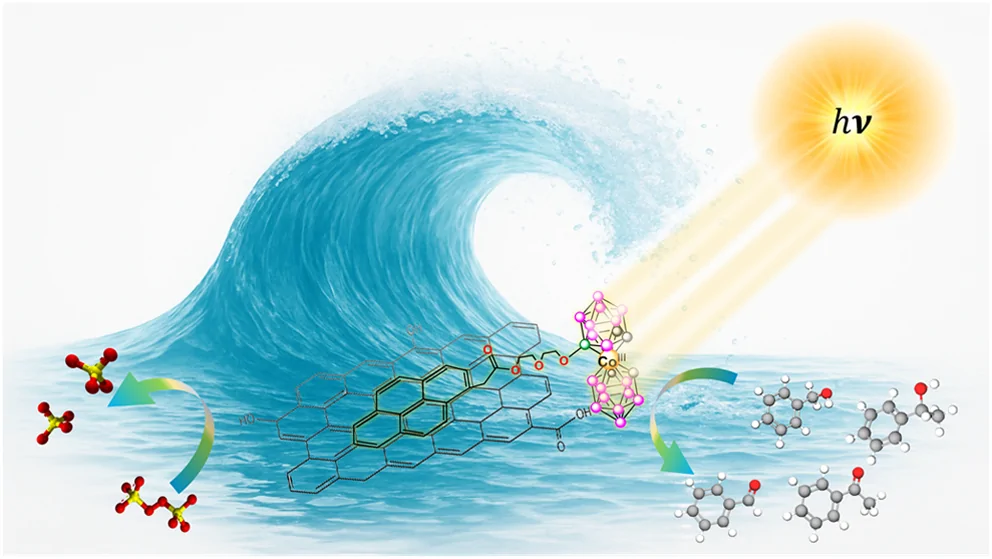

## Introduction

The catalytic oxidation of alcohols to carbonyl compounds is a cornerstone of synthetic chemistry, with profound implications ranging from the production of fine chemicals and pharmaceuticals,^1–3^ to the advancement of hydrogen-based energy technologies.^4,5^ Traditionally, these transformations have relied on noble metal catalysts, organic oxidants, and volatile organic solvents.^6,7^ However, the current shift toward sustainable chemistry demands the development of environmentally friendly processes that operate under mild conditions, ideally utilizing earth-abundant materials and benign reaction media.^8^

Photoredox catalysis in aqueous media has emerged as a compelling strategy for alcohol photooxidation, leveraging water as an abundant, non-toxic, and biocompatible solvent.^9–13^ Integrating water-soluble photocatalysts into industrial and pharmaceutical workflows offers significant advantages in terms of scalability and waste reduction.^14^ In this context, molecular catalysts with high stability and tunable electronic properties are essential for optimizing performance in such demanding environments.

In parallel with the development of sustainable photocatalytic processes, boron science has advanced rapidly owing to the unique electronic properties, chemical robustness and structural versatility of boron-based materials.^15^ These materials have found widespread applications in photocatalysis, electrocatalysis, energy conversion and storage, environmental remediation, sensing, nanoelectronics and biomedicine.^16–22^ Beyond preliminary work with metallacarboranes supported on functionalized graphene oxide (GO),^23^ the integration of molecular boron clusters with carbon nanomaterials for recyclable photocatalysts in aqueous media remains largely unexplored.

θ-metallacarborane compounds, containing two such icosahedral [7,8-*nido*-C_2_B_9_H_11_]^2–^ units, among them Na­[3,3′-Co­(1,2-C_2_B_9_H_11_)_2_], exhibit great stability due to the combined structural robustness and 3D aromatic properties,^24^ which contribute to their exceptional chemical and thermodynamic stability. We have recently demonstrated that these θ-metallacarboranes act as a highly efficient and selective photocatalysts for the oxidation of alcohols and alkenes in water.^25–28^ Their superior performance is attributed to the high water solubility of their sodium salts, a potent (Co^4+/3+^)^29,30^ redox couple, the absence of fluorescence and their unique surfactant-like behavior.^31^ Moreover, these clusters exhibit a rich landscape of non-covalent interactions, including C_c_–H···O hydrogen bonds and C_c_–H···H–B dihydrogen bonding, which govern their self-assembly and reactivity.^32–39^

Despite their high activity, the transition from homogeneous to large-scale industrial applications is often hindered by catalyst recovery and sustainability concerns. Heterogenization offers a viable solution, facilitating simpler workups, recyclability, and continuous operation.^40^ Recent studies have further demonstrated that immobilization of cobaltabis­(dicarbollide) within chiral silica nanohelices affords recyclable heterogeneous photocatalysts capable of promoting enantioselective photooxidation of aromatic secondary alcohols in water, highlighting the versatility of supported metallacarborane-based catalytic systems.^41^ While we previously reported the heterogenization of cobaltabis­(dicarbollide) onto silica-coated magnetite,^14^ exploring new supports with high surface areas remains a priority, particularly considering that oxidizing agents are generated and that the grafting of the catalyst onto the support may be compromised if the linker contains several −CH_2_– groups.

The zwitterionic 8-dioxanate derivative, [3,3′-Co­(8-(CH_2_CH_2_O)_2_-1,2-C_2_B_9_H_10_)­(1′,2′-C_2_B_9_H_11_)] **2** (see Chart 1),^42,43^ serves as a versatile platform for such functionalization. Through nucleophilic ring-opening of the oxonium fragment, a wide array of ligands can be introduced.^44–46^ However, the functionalization of metallacarboranes with polycyclic aromatic groups for non-covalent anchoring onto carbon-based nanomaterials remains unexplored.

**Chart 1:**
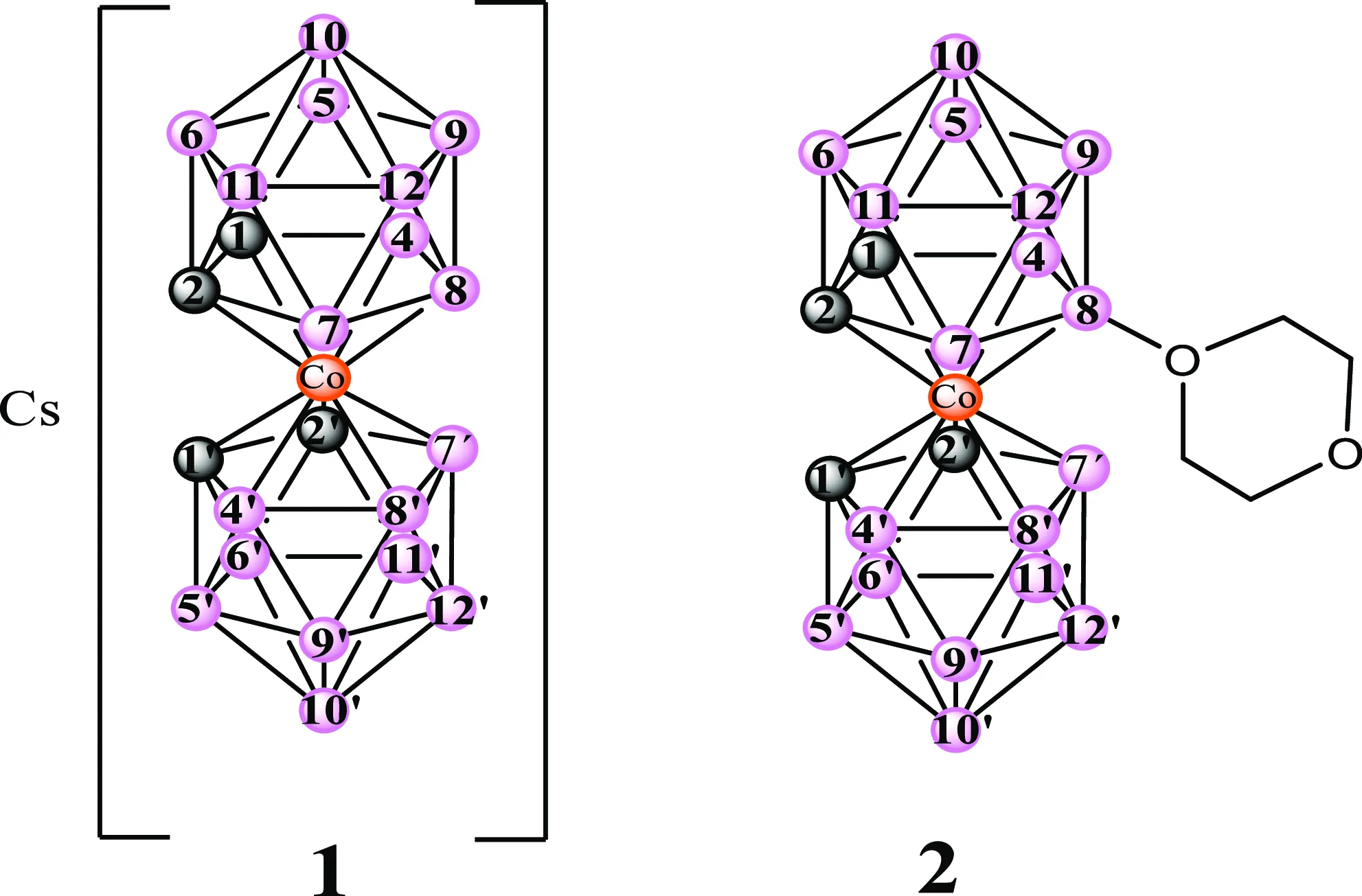
Starting Materials Used in This Work

Graphene oxide (GO) represents an ideal support due to its high dispersibility in water and its hybrid aromatic/aliphatic structure, which allows for diverse supramolecular interactions.^47,48^ By employing non-covalent functionalization such as π–π and CH–π interactions, it is possible to enhance catalyst stability and dispersibility without altering the intrinsic electronic structure of the support.^12,49^ Moreover, GO can act as an electron sink, potentially suppressing charge recombination and enhancing photocatalytic efficiency.^50^

In this work, we report a straightforward synthetic route to obtain new pyrene- functionalized metallacarborane derivatives, Na­[**3**] and [N­(CH_3_)_4_]­[**3**] (Scheme 1) and the immobilization of Na­[**3**] onto GO to yield the hybrid material GO@**3** (Scheme 2). We provide a comprehensive characterization of these systems in both solution and the solid state. Furthermore, we evaluate their performance as both homogeneous and heterogeneous photocatalysts for alcohol oxidation in water under UVA irradiation. Our results demonstrate that anchoring the catalyst to GO not only improves yields through enhanced substrate-catalyst interactions and facilitated electron transfer but also enables efficient catalyst recycling, paving the way for more sustainable photocatalytic transformations.

**Scheme 1:**
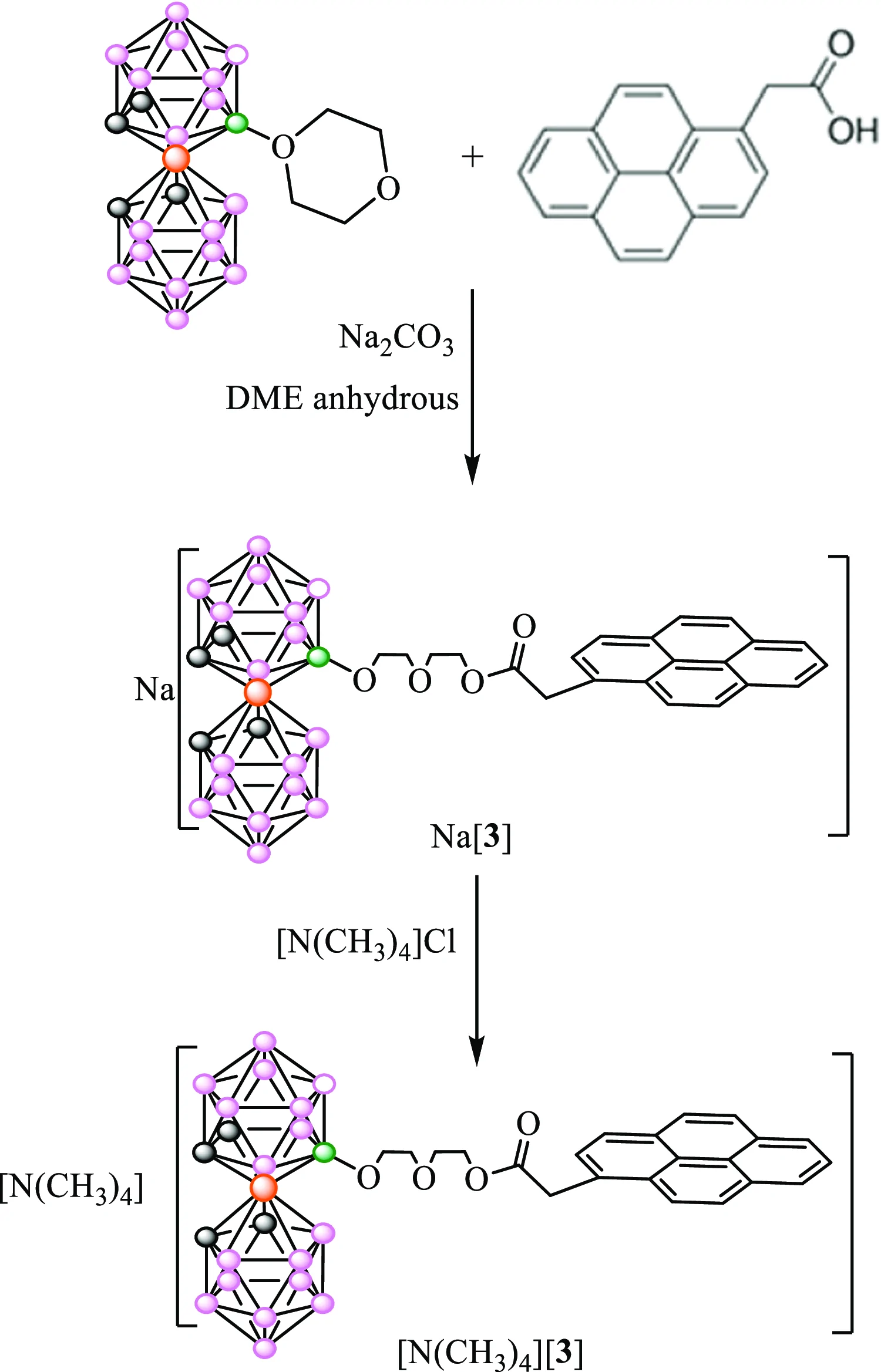
Synthetic Strategy Used for the Formation of Na­[**3**] and [N­(CH_3_)_4_]­[**3**]

**Scheme 2:**
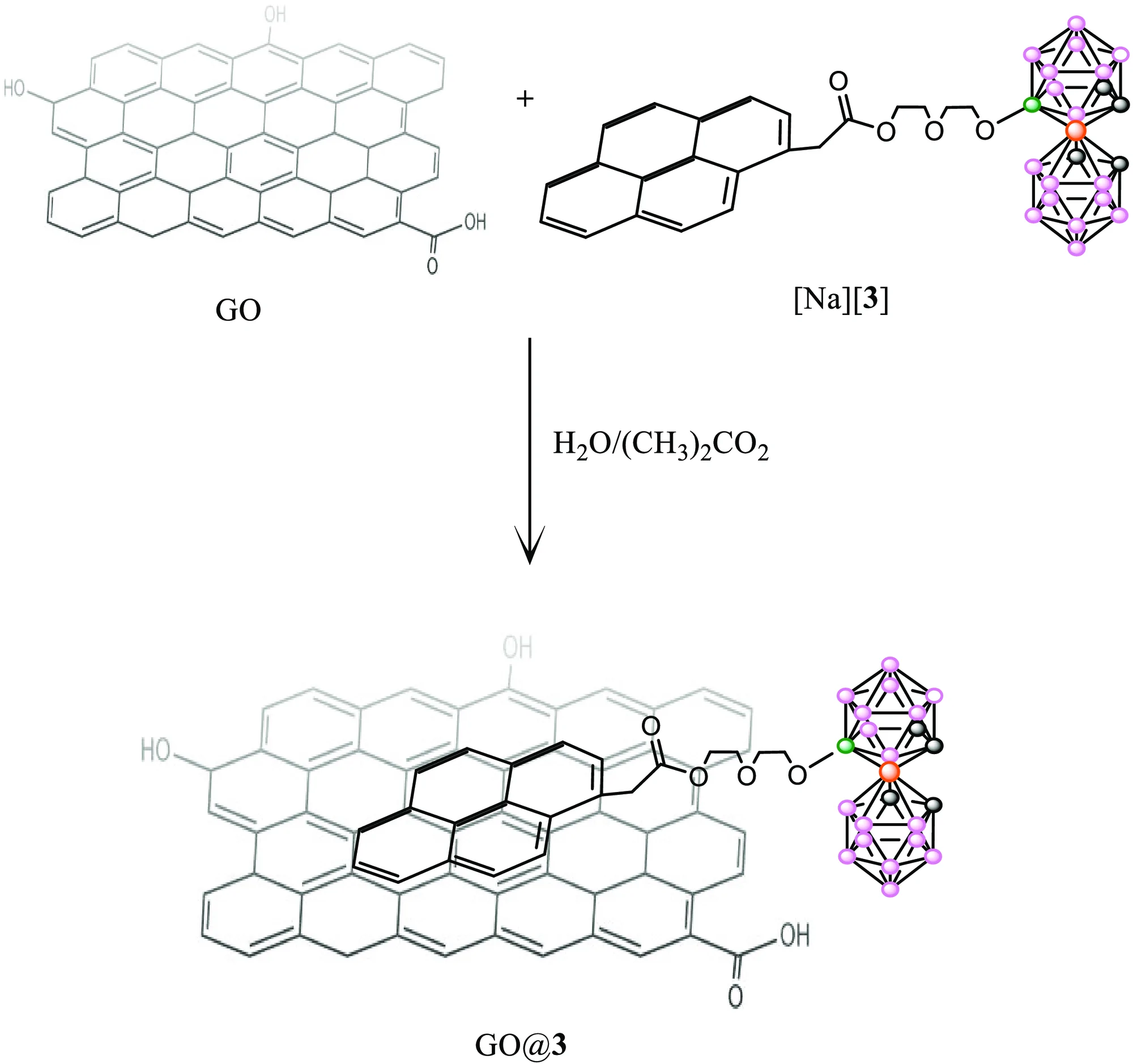
Synthetic Strategy Used for the Immobilization of Na­[**3**] onto GO

## Materials and Methods

### Materials

All reagents used in the present work were obtained from Sigma-Aldrich and were used without further purification. Cs­[*o*-COSAN], **1** was obtained from Katchem Spol.sr.o (Kralupy nad Vltavou, Czech Republic). Reagent grade organic solvents were obtained from Carlo Erba and high purity de-ionized water was obtained by passing distilled water through a nano-pure Mili-Q water purification system.

All prepared θ-metallacarborane complexes are air stable. All manipulations were carried out under nitrogen atmosphere. DMF was distilled from sodium benzophenone prior to use. [3,3′-Co­(8-C_4_H_8_O_2_-1,2-C_2_B_9_H_10_)­(1′,2′-C_2_B_9_H_11_)], **2**, was prepared according to the literature.^42,43^

### Instrumentation and Measurements

IR spectra were captured using an Agilent Cary 630 FTIR spectrometer with an ATR MK-II Golden Gate Single Reflection system. UV-Vis spectroscopy was carried out using a Cary 50 Scan (Varian) UV-Vis spectrophotometer with 1 cm quartz cells. For CV and DPV experiments, an IJ Cambria 660C potentiostat with a three-electrode cell setup was employed. A Glassy Carbon electrode (3 mm diameter) from BAS served as the working electrode, while platinum wire and SCE (Saturated Calomel Electrode) were used as auxiliary and reference electrodes, respectively. All cyclic voltammograms were recorded under a nitrogen atmosphere. The solvent for the complexes contained [N*n*-Bu_4_]­[PF_6_] (TBAPF_6_) as a supporting electrolyte to achieve a 0.1 M ionic strength solution. *E*_1/2_ values were determined from CV experiments as the average of the oxidative and reductive peak potentials [(Epa + Epc)/2], or directly from DPV. Unless otherwise specified, the complexes were at a concentration of approximately 1 mM. The ^1^H and ^1^H­{^11^B}-NMR ^13^C­{^1^H}-NMR and ^11^B and ^11^B­{^1^H}-NMR spectra were recorded on a Bruker DPX 400 MHz instrument equipped with the appropriate decoupling accessories. All NMR spectra were performed in the indicated deuterated solvent at 22 °C. The ^11^B and ^11^B­{^1^H}-NMR chemical shifts were referenced to external BF_3_·OEt_2_. Samples for the complexes characterization were registered in (CD_3_)_2_CO and for the catalytic reaction products were registered in CDCl_3_. Chemical shifts are reported in units of parts per million downfield from reference, and all coupling constants in Hz. Elemental analyses were carried out using a CHNS-O Elemental Analyzer EA-1108 from Fisons. ESI-MS experiments were conducted on a Navigator LC/MS system from Thermo Quest Finnigan with acetonitrile as the mobile phase. TEM studies utilized a JEOL JEM 1210 operating at 120 kV. Scanning electron microscopy (SEM) and EDX analyses were performed using the QUANTA FEI 200 FEG-ESEM and FESEM Hitachi S4100, respectively. Cobalt loading was quantified by inductively coupled plasma-atomic emission spectrometer (ICP-AES, Agilent 7500c). Samples were digested in a HCl/HNO_3_ mixture at room temperature prior to analysis. XPS measurements were carried out at room temperature using a SPECS PHOIBOS 150 hemispherical analyzer with monochromatic Al Kα radiation (1486.74 eV) as the excitation source. Thermogravimetric analysis (TGA) was performed using a Netzsch STA 449 thermal analyser at a heating rate of 10 °C min^–1^ under an Ar atmosphere.

The hydrodynamic diameter of the samples dispersed in water/acetone were studied by dynamic light scattering (DLS) in a Zetasizer Nano ZS (Malvern Instruments Ltd) equipped with a He–Ne 633 nm laser using 1 mL of the sample’s dispersion in a disposable glass cuvette. For each compound a couple of samples were run, and the measurements were done in triplicate at ambient temperature with multiple sub-runs. The aqueous sample solutions were previously filtered using a syringe filter (PTFE, 0.2 μm pore diameter).

### Preparations

#### Synthesis of Na­[3,3′-Co­(8-O­(CH_2_CH_2_O)_2_C­(O)-(CH_2_–pyrene)­(1,2-C_2_B_9_H_10_)-1,2-C_2_B_9_H_11_], Na­[**3**]

Under an inert atmosphere, a dry mixture of pyreneacetic acid (0.032 g, 0.122 mmol) and sodium carbonate (0.041 g, 0.3 mmol) was refluxed in 10 mL of anhydrous DME for 2 h. Subsequently, a solution of **2** (0.050 g, 0.122 mmol) in 5 mL of DME was added dropwise to the solution. Refluxing was continued overnight. The excess sodium carbonate was then filtered off and discarded. After solvent removal, the product was isolated, washed with petroleum ether, and dried under vacuum. 0.042 g (50 %) of Na­[**3**]. Elemental analysis for Na­[CoO_4_B_18_C_26_H_40_]·2 DME: Calc.: C, 46.74; H, 6.92. Found: C, 47.03; H, 6.55. IR (ν): 3037, 2921, 2869, 2530, 1714, 1450, 1244, 1091, 972, 845 cm^–1^.

^1^H NMR (δ): 8.36 (d, 1H, C_16_H_9_), 8.33–8.25 (m, 4H, C_16_H_9_), 8.14 (d, 2H, C_16_H_9_), 8.09–8.05 (m, 2H, C_16_H_9_), 4.46 (s, 2H, COCH_2_), 4.31 (br s, 4H, C–H), 4.28 (t, 2H, OCH_2_CH_2_), 3.67 (t, 2H, OCH_2_CH_2_), 3.57 (t, 2H, OCH_2_CH_2_), 3.49 (t, 2H, OCH_2_CH_2_).^13^C­{^1^H} NMR (δ): 171.08 (CO), 128–123.6 (C_pyrene_), 71.88 (C_8_), 68.78 (C_6_), 68.36 (C_7_), 64.06 (C_5_), 54.43 (C_1–4_), 38.63 (C_9_).^11^B NMR (δ): 22.90 (s, 1B, B(8)), 3.83 (d, 1B), 0.40 (d, 1B), −2.33­(d, 1B), −4.19 (d, 2B), −7.20 (d, 2B), −7.84 (d, 4B), −17.22 (d, 2B), −20.38 (d, 2B), −22.14 (d, 1B), −27.73 (d, 1B). ESI-MS (*m*/*z*): 716.4 [**3** + 2Na]^+^;732.4 [**3** + Na + K]^+^; 670.2 [**3**]^−^; 686.2 [**3** + O]^−^. *E*_1/2_ (Co^IV/III^) = 1.53 V and *E*_1/2_ (Co^III/II^) = −1.36 V *vs* SCE.

#### Synthesis of [N­(CH_3_)_4_]­[3,3′-Co­(8-O­(CH_2_CH_2_O)_2_COCH_2_(C_16_H_9_)-1,2-C_2_B_9_H_10_)­(1′,2′-C_2_B_9_H_11_)], [N­(CH_3_)_4_]­[**3**]

0.017 g (0.024 mmol) of Na­[**3**] was dissolved in the minimum volume of diethyl ether, and an aqueous solution containing an excess of [N­(CH_3_)_4_]­Cl was added. This led to the formation of an orange precipitate, which was isolated, washed with petroleum ether, and subjected to vacuum drying to obtain the final product. Yield: 0.010 g (55 %). Elemental analysis for [N­(CH_3_)_4_]­[CoO_4_B_18_C_26_H_40_]·1.5 DME·0.5­[N­(CH_3_)_4_]­Cl: Calc.: C, 48.8; H, 7.8; N, 2.24 Found: C, 49.30; H, 7.29; N, 2.60. IR (ν): 3036, 2928, 2914, 2854, 2527, 1717, 1479, 1441, 1248,1095, 972, 942, 846 cm^–1^.^1^H NMR (δ): 8.38 (d, 1H, C_16_H_9_), 8.33–8.23 (m, 4H, C_16_H_9_), 8.16 (d, 2H, C_16_H_9_), 8.11-8.04 (m, 2H, C_16_H_9_), 4.48 (s, 2H, COCH_2_), 4.32 (br s, 4H, C–H), 4.28 (t, 2H, OCH_2_CH_2_), 3.67 (t, 2H, OCH_2_CH_2_), 3.59 (t, 2H, OCH_2_CH_2_), 3.51­(t, 2H, OCH_2_CH_2_), 3.45 (s, 12H, N­(CH_3_)_4_).^13^C­{^1^H} NMR (δ): 171.13 (s, COO), 130–122. (m, C_16_H_9_), 72.58­(C_8_), 68.75 (C_7_), 64.14 (C_6_), 64­(C_5_), 55 (N­(CH_3_)_4_) 46.43­(C_1–4_), 38.66 (C_9_).^11^B NMR (δ): 22.85 (s, 1B, B(8)), 3.84 (d, 1B), 0.38 (d, 1B), 0.76 (d, 1B), −2.43­(d, 1B), −4.25 (d, 2B), −7.84 (t, 6B), −17.28 (d, 2B), −20.44 (d, 2B), −22.60 (d, 1B), −28.42 (d, 1B). ESI-MS (*m*/*z*): 818.6 [**3** + 2N­(CH_3_)_4_]^+^; 670.4 [**3**]^−^; 758.2­[**3** + (OCH_2_CH_2_)_2_]^−^. *E*_1/2_ (Co^IV/III^) = 1.55 V and *E*_1/2_ (Co^III/II^) = −1.34 V *vs* SCE.

### Functionalization of GO with Na[3] Complex, GO@[3]

0.050 g of GO was dispersed in 25 mL of water/acetone (3:1 ratio) containing Na­[**3**] (0.10 mM). The mixture was sonicated in an ultrasound bath for 30 min and the suspension was stirred for 24 h at room temperature to yield the immobilized complex GO@[**3**]. This compound was then filtered, washed with the same solvent and dried under vacuum. The quantification of the anchored complex was done through ICP-AES.

### Homogeneous Photocatalytic Studies

An aqueous solution (5 mL) at pH 7 (K_2_CO_3_ solution is used to adjust the pH) containing Na­[**3**] as photocatalyst, alcohol as the substrate, and Na_2_S_2_O_8_ as a sacrificial acceptor was placed in a quartz tube. This setup was then exposed to UVA-I light (2.2 W, λ = 352 nm, Long/Ultra-long UVA) for different times, at room temperature and atmospheric pressure. Different ratios of complex/substrate/sacrificial oxidant were employed (1:10000:20000 and 1:1000:2000, corresponding to concentrations of 0.002:20:40 and 0.020:20:40 mM, respectively). The resultant solutions were extracted thrice with CH_2_Cl_2_, dried with anhydrous sodium sulfate, and the solvent was then evaporated under reduced pressure. To ensure repeatability, all reactions were conducted in triplicate. The reaction products were quantified and characterized using ^1^H NMR spectroscopy.

### Heterogeneous Photocatalytic Studies

Alcohol (1 mmol) and Na_2_S_2_O_8_ (2 mmol) were dissolved in 5 ml of water (K_2_CO_3_, pH = 7) together with GO@**3** (0.10 μmol). The amount of heterogenized catalyst was calculated considering the functionalization of the GO support. By using [N­(CH_3_)_4_]­[**3**] as photocatalyst, the concentration values were, catalyst (1.30 μmol), substrate (0.26 M) and Na_2_S_2_O_8_ (0.53 M).The photocatalytic oxidation experiments were all performed by exposing the reaction quartz vials to UVA-I irradiation (λ ∼ 352 nm), at room temperature and atmospheric pressure, for different times. For every experiment, light illumination was supplied by a light reactor with 12 lamps that produce UVA-I light at room temperature. The reaction products were extracted with dichloromethane (3 × 10 mL). The combined organic phases were dried over sodium sulfate and the solvent was evaporated under reduced pressure. To check the reproducibility of the reactions all the experiments were carried out three times. The reaction products were quantified using ^1^H-NMR spectroscopy.

### Recycling Experiments

The reaction conditions indicated above were used in these experiments. After finishing each run, the heterogeneous catalyst was separated by centrifugation, washed with water and was reused in a subsequent run.

## Results and Discussion

### Synthesis and Spectroscopic Characterization of Molecular Compounds

The reaction of Cs­[3,3′-Co­(C_2_B_9_H_11_)_2_], (Cs­[*o*-COSAN]), **1** with BF_3_.Et_2_O in refluxing dioxane affords the zwitterionic species [3,3′-Co­(8-C_4_H_8_O_2_-1,2-C_2_B_9_H_10_)­(1′,2′-C_2_B_9_H_11_)], **2**.^42,43^ As shown in Chart 1, the oxonium oxygen atom in **2** is highly susceptible to nucleophilic attack, making it an excellent precursor for further functionalization. In this work, we have synthesized new derivatives based on the [*o*-COSAN]^−^ framework via the ring-opening reaction of cyclic oxonium **2** with 1-pyreneacetic acid. The synthetic route for the preparation of the Co (III) complexes Na­[**3**] and [N­(CH_3_)_4_]­[**3**], is displayed in Scheme 1. The sodium salt of the pyrene ligand, is generated *in situ* from 1-pyreneacetic acid and sodium carbonate; the resulting nucleophile then promotes the ring-opening of **2** to yield the monoanionic species Na­[3,3′-Co­(8-O­(CH_2_CH_2_O)_2_C­(O)-(CH_2_–pyrene)­(1,2-C_2_B_9_H_10_)**-**1′,2′-C_2_B_9_H_11_], denoted as Na­[**3**]. Subsequent cation exchange (metathesis) with tetramethylammonium chloride afforded the corresponding salt [N­(CH_3_)_4_]­[**3**].

The proposed structures were confirmed by FTIR, multinuclear NMR spectroscopy, ESI-MS, UV–vis spectroscopy and thermogravimetric analysis (Figures S1–S7). The ^1^H-NMR spectra of Na­[**3**] and [N­(CH_3_)_4_]­[**3**] show two distinct set of resonances: one in the aromatic region, corresponding to the protons of the pyrene ring, and another in the aliphatic region. The methylene protons attached to the pyrene ring appear as a singlet at 4.50 ppm, while a signal integrating for four protons, attributed to the C_cluster_-H protons, appears around 4.32 ppm. In both compounds, signals corresponding of the B(8)-OCH_2_–, −CH_2_OCH_2_– and −CH_2_–OCO- groups are observed in the range of 4.34–3.40 ppm. For compound [N­(CH_3_)_4_]­[**3**], the resonance corresponding to the methyl protons of the [N­(CH_3_)_4_]^+^ cation appears as a singlet at 3.45 ppm. The ^1^H­{^11^B} NMR spectra show signals in the range of 3–1.25 ppm, which are assigned to B–H protons in both compounds. The ^11^B­{^1^H} NMR spectra are fully consistent with the expected structures. In particular, the characteristic resonance assigned to the substituted B(8) vertex appears as a singlet at δ + 23 ppm (Figure 1), providing clear evidence for the regioselective functionalization of the metallacarborane cage. The remaining boron resonances display the characteristic intensity pattern (1:1:1:1:2:2:4:2:2:1:1) expected for an asymmetric cobaltabis­(dicarbollide) framework.

**Figure 1:**
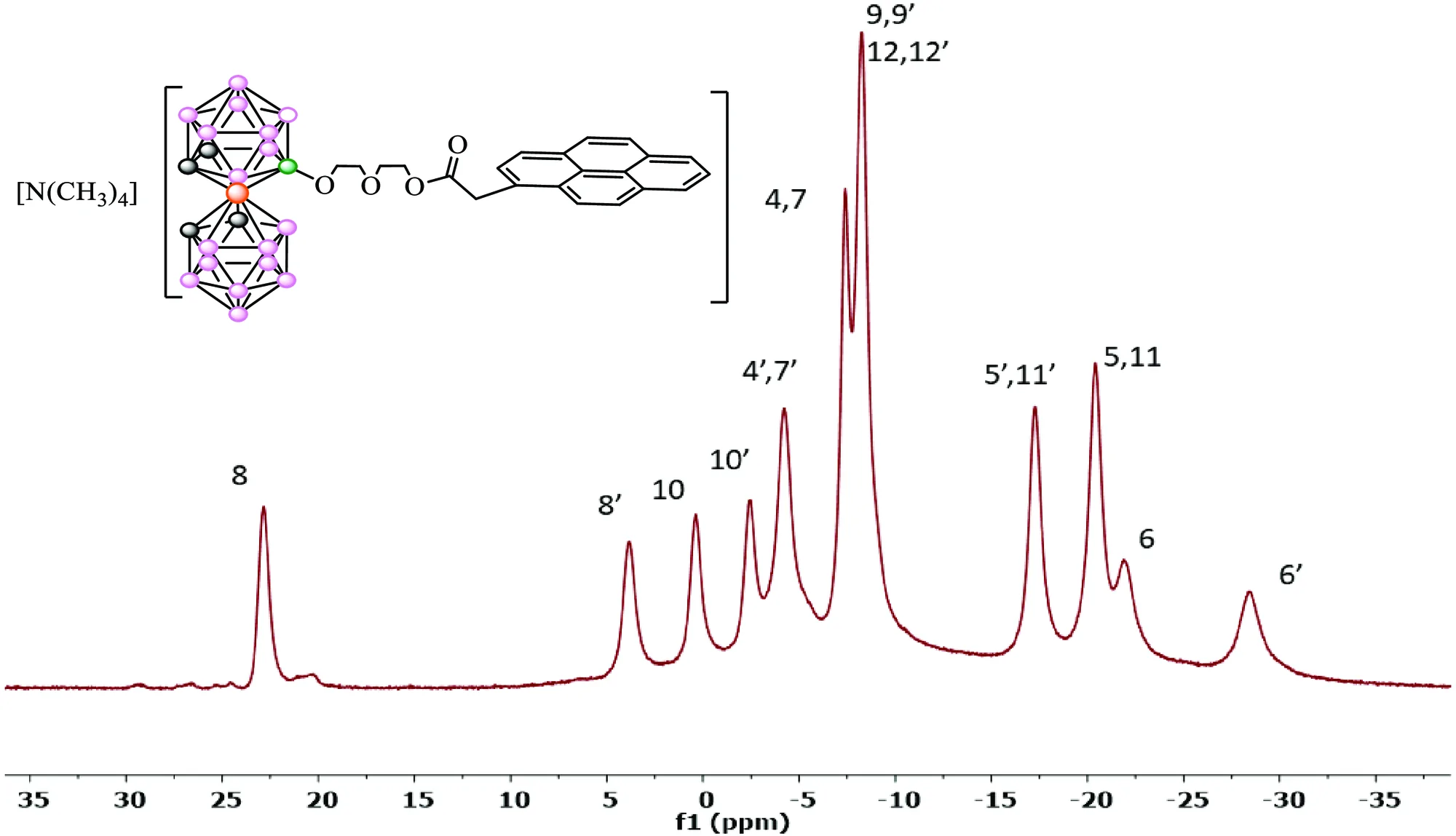
^11^B­{^1^H} NMR spectrum of [N­(CH_3_)_4_]­[**3**] in *d*_6_-acetone at room *T*.

UV–vis spectra exhibits intense bands between 230 and 345 nm, assigned to π–π* transitions of the pyrene chromophore that partially eclipse the absorptions corresponding to the cobaltabis­(dicarbollide) moiety. The absorption band around 300 nm is tentatively assigned to the ligand-to-metal charge-transfer (LMCT) transition of the cobaltabis­(dicarbollide) framework.^25,51^ Compared with precursor 2, the slight bathochromic shift of this transition reflects the electronic influence of the pyrene-containing substituent. It is worth noting that replacement of [Na]^+^ by [N­(CH_3_)_4_]^+^ has a negligible effect on the electronic absorption spectra.

ESI-MS analyses further confirmed the molecular composition of both derivatives. In negative ion mode, both spectra exhibit a base peak at *m*/*z* 670.2 and 670.4, corresponding to the monoanionic species [**3**]^−^. Additionally, a peak at *m*/*z* 686.2 for Na­[**3**], corresponds to [**3**+O]^‑^ species, while the peak at *m*/*z* 758.2 for [N­(CH_3_)_4_]­[**3**] corresponds to the [**3**+ (OCH_2_CH_2_)_2_]^−^ fragment.^43^ In positive ion mode, Na­[**3**] displays peaks at *m*/*z* 716.4 and 732.4, which are attributed to the [**3** +2 Na^+^]^+^ and [**3** + Na + K]^+^ adducts, respectively. For [N­(CH_3_)_4_]­[**3**], a peak at *m*/*z* 818.6 is observed, consistent with the [**3** + 2­[N­(CH_3_)_4_]]^+^ ion.

Thermogravimetric analysis (TGA) of Na­[**3**] revealed a three-step thermal decomposition profile. The initial weight loss below 100 ^ο^C is associated with the removal of residual solvent molecules. Two subsequent degradation steps occurring between 100 and 500 °C, result in a cumulative mass loss of 37.5%, which is in good agreement with the calculated value for the elimination of the C­(O)­CH_2_–pyrene moiety.

### Electrochemical Properties

Electrochemical studies of 1-pyreneacetic acid and complexes **2**, Na­[**3**] and [N­(CH_3_)_4_]­[**3**] were performed in CH_3_CN containing 0.1M tetrabutylammonium hexafluorophosphate (TBAPF_6_) as supporting electrolyte, using a glassy carbon working electrode. Representative voltammograms are shown in Figure 2, while the complete electrochemical data are provided in Figures S8–S10.

**Figure 2:**
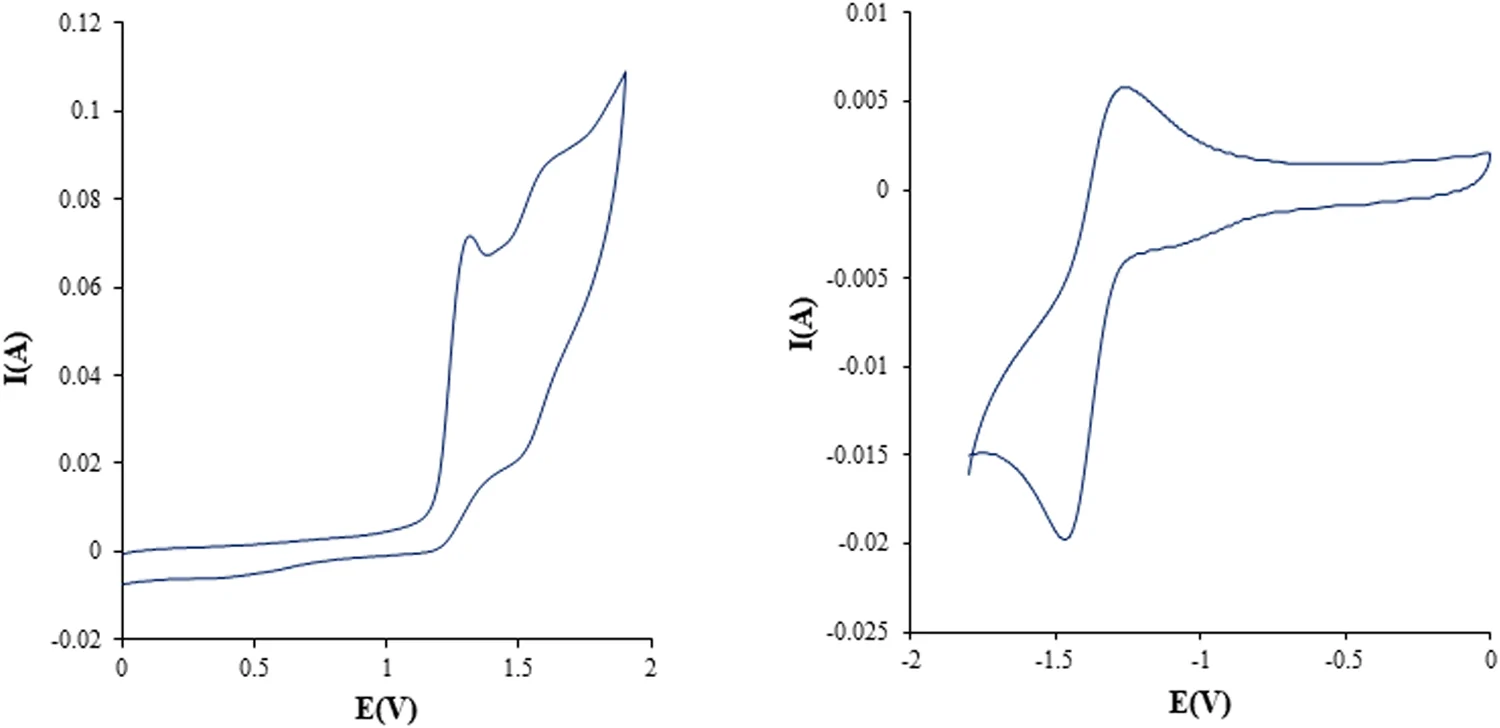
CVs of Na­[**3**] in CH_3_CN (TBAPF_6_ 0.1 M) *vs* SCE, showing the anodic (left) and cathodic (right) regions.

1-pyreneacetic acid exhibits an irreversible oxidation peak at 1.34 V *vs* SCE, attributed to the formation of the pyrene radical cation.^52^ The zwitterionic species **2**, displays an irreversible oxidation wave at *E*_pa_= 1.60 V *vs* SCE, corresponding to the Co­(IV)/Co­(III) redox couple. Furthermore, two reversible reduction waves are observed at *E*_1/2_ = −0.95 and −1.34 V *vs* SCE; the first wave shows higher current intensity than the second. The first reduction is tentatively assigned to the oxonium moiety of compound **2**, while the second corresponds to the Co­(III) to Co­(II) reduction.

The pyrene-functionalized derivatives Na­[**3**] and [N­(CH_3_)_4_]­[**3**] exhibit very similar electrochemical responses, indicating that replacement of the counterion has only a minor influence on their redox behaviour. The Co­(III)/Co­(II) redox couple appears at *E*_1/2_ = −1.36 V (Δ*E* = 200 mV) for Na­[**3**] and −1.34 V (Δ*E* = 130 mV) *vs* SCE for [N­(CH_3_)_4_]­[**3**], while the Co­(IV)/Co­(III) couple is observed at *E*_1/2_ = 1.52 and 1.54 V *vs* SCE, respectively. These redox couples observed are tentatively assigned to the Co­(III)/Co­(II) and Co­(IV)/Co­(III) transitions, respectively (Figures 3 and S9). Although electrochemical data alone provide an indirect assignment, these attributions are strongly supported by well-established literature precedents in related cobaltabis­(dicarbollide) systems.^30,53^ Direct spectroscopic confirmation of these specific oxidation states remains outside the scope of the present study. Additionally, both compounds display an irreversible oxidation peak around 1.32–1.34 V, characteristic of the pyrene moiety. As we have observed, the choice of cation results in a redox potential difference of only 20 mV.

**Figure 3:**
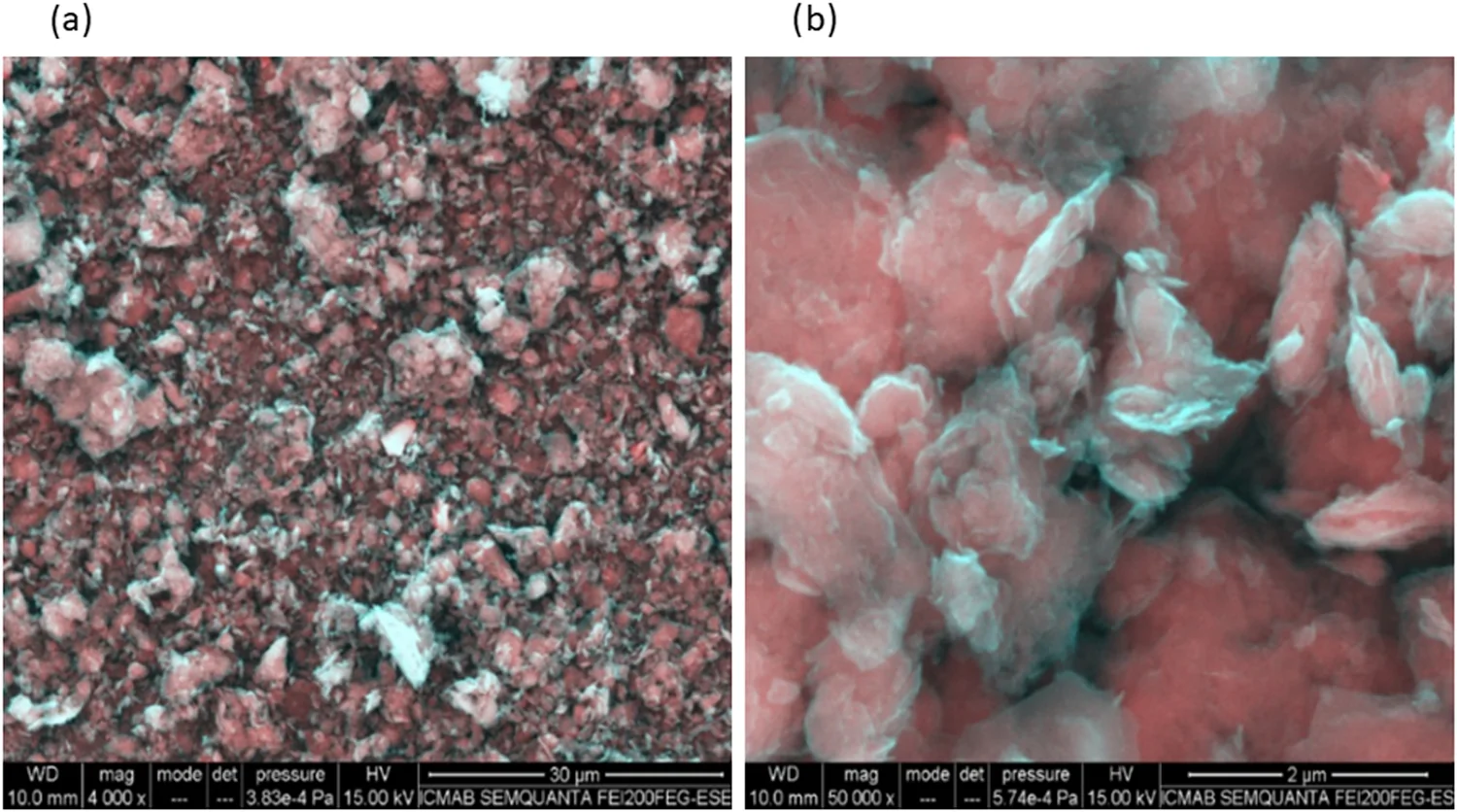
SEM images of GO@**3**, (the bars indicate a length of (a) 30 μm and (b) 2 μm).

### Dynamic Light Scattering (DLS) Studies

Dynamic light scattering (DLS) measurements were conducted at two different concentrations (0.02 and 0.002 mM) for Na­[**3**] and [N­(CH_3_)_4_]­[**3**] in a H_2_O/acetone (1:1) mixture due to the insolubility of [N­(CH_3_)_4_]­[**3**] in water. Figure S11 shows the size distribution graphs. At the higher concentrations (0.02 mM), both compounds form nanometric aggregates,^33^ with average hydrodynamic radii of approximately 140 nm for Na­[**3**] and 168 nm for [N­(CH_3_)_4_]­[**3**]. The polydispersity index (PDI) values are 0.232 and 0.009, respectively, indicating a significantly higher degree of particle size uniformity for [N­(CH_3_)_4_]­[**3**].

These results are consistent with the different influence exerted by the cation on the structure of these compounds, where it was observed that while [N­(CH_3_)_4_]^+^ favors the elongated form of the polyether chain, (CH_2_CH_2_O)_2_, the Na^+^ cation prevents this elongation due to its coordination with three of the oxygen atoms of this polyether chain.^44^ Although the results were not entirely satisfactory at low concentrations (0.002 mM), they seem to suggest the formation of a somewhat higher number of aggregates.

### Synthesis and Characterization of Heterogeneous Material

We have explored the functionalization of graphene oxide (GO) with the molecular compound Na­[**3**] in a single step, to obtain the hybrid material GO@[**3**] (Scheme 2). The synthesis approach involved preparing a dispersion containing 50 mg of GO in a 3:1 water/acetone mixture of Na­[**3**], followed by sonication for 30 min. The resulting dispersion was stirred for 24 h at room temperature to yield the immobilized GO@**3**.

The synthetic strategy relies on the non-covalent anchoring^54^ of Na­[**3**] onto GO, primarily driven by π–π stacking between the pyrene fragment and the GO surface. The dominance of this interaction was confirmed by a control experiment; the adsorption of the parent (non-functionalized) metallacarborane onto GO was found to be five times lower than that of the pyrene-derivative Na­[**3**]. This five-fold increase clearly demonstrates that π–π interactions involving the pyrene moiety are the primary driving force for anchoring, significantly outweighing B–H or other non-bonding interactions between the carborane cage and the GO surface. To the best of our knowledge, this represents the first example of a monoanionic molecular species incorporating a functionalized cobaltabis­(dicarbollide) anion bearing a pyrene moiety, anchored onto GO support through non-covalent interactions. This stands in contrast to previously reported cobaltabis­(dicarbollide) derivatives, which have relied exclusively on covalent bonding.^55^ The loading of Na­[**3**] onto the GO support was evaluated by UV-vis spectroscopy in a water/acetone mixture. A decrease in the initial absorbance of the band at 342 nm was observed for a 0.1 mM solution of Na­[**3**] after 24 h of reaction in contact with the support. This decrease indicated that 95% of [**3**]^−^ complex was successfully anchored onto the GO, resulting in the formation of GO@**3** (Figure S12). ICP analysis of Co indicated that 4.76 μmol of complex was anchored per 100 mg of GO@**3**. Successful formation of the hybrid material was confirmed by complementary microscopic and spectroscopic techniques (Figures 3, 4 and S13–S15).

**Figure 4:**
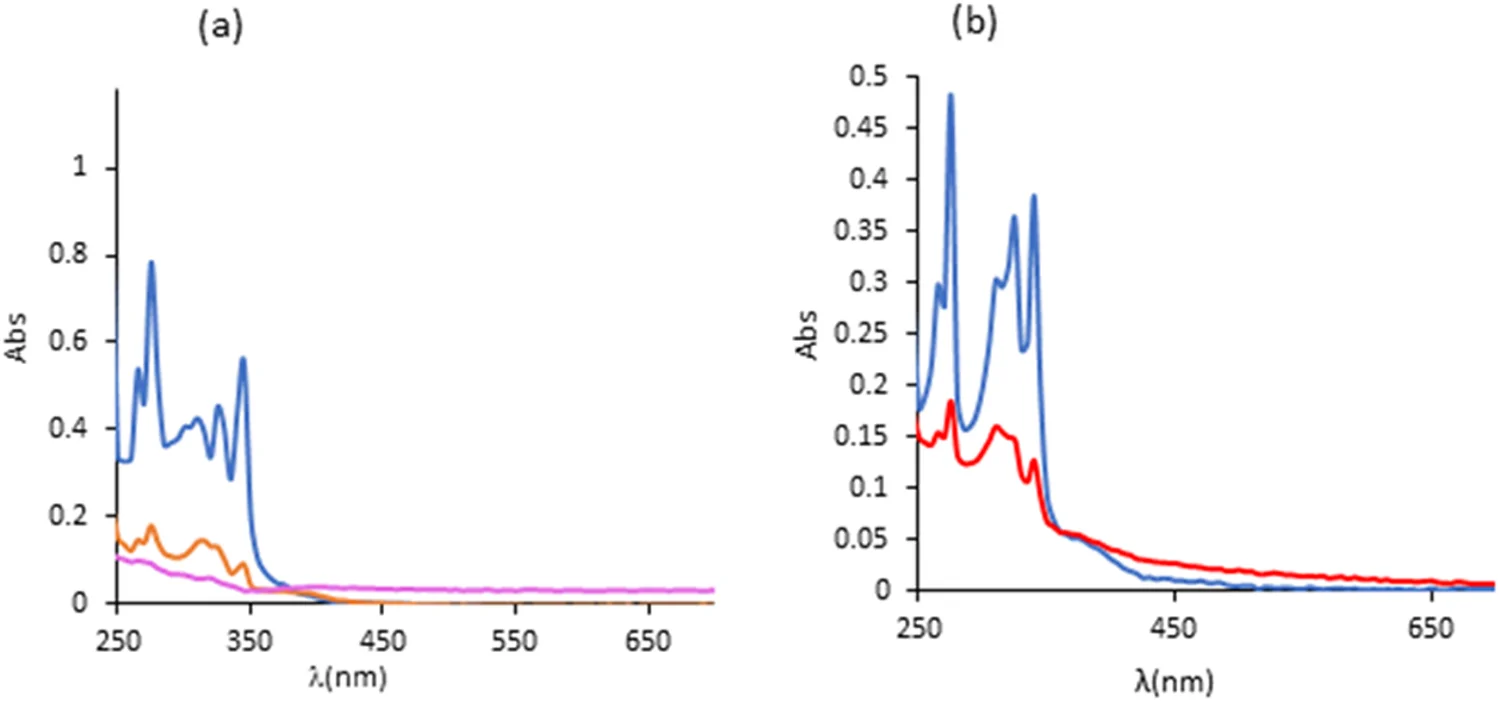
UV-vis spectra of Na­[**3**] (blue line), GO@**3** (orange line) and GO (pink line) in (a) CH_2_Cl_2_ and (b) Na­[**3**] (blue line), GO@**3** (red line) in CH_3_CN.

Scanning electron microscopy (SEM) and transmission electron microscopy (TEM) images reveal the characteristic morphology of the hybrid material, with the formation of aggregates onto the surface with irregular shapes and the formation of exfoliated structures. Whereas, energy dispersive X-ray spectroscopy (EDX) analysis confirms the presence of Co and B in the hybrid material. X-ray photoelectron spectroscopy (XPS) further supports the successful immobilization through the appearance of the characteristic peaks for C 1s, O 1s and B 1s centered at 284.4, 533, and 188 eV, respectively. The peak of C 1s corresponds to different functional groups (C–C/C=C/C–O/O–C=O) present in the material. The deconvolution of the signal revealed contributions around 284.4 eV (C–C/C=C), 285.5 eV (C–O) and 289 eV (O–C=O). Moreover, the less intense peak at 188 eV, assigned to B 1s corroborates the presence of θ-metallacarborane onto the surface of GO.

UV-vis spectrum of GO@**3** (Figure 4) was registered on a suspension of the heterogeneous material in dichloromethane and acetonitrile. We prepared dispersions of GO@**3** with concentration of 1 mg/5 ml from its dry powder. The dispersion is sonicated for 15 min and centrifugated at 4010 rpm for 30 min to remove aggregates. UV-vis exhibits a similar pattern to that of the homogeneous Na­[**3**], observing the bands of greatest absorption between 220 and 350 nm in agreement with that observed in the Na­[**3**] compound.

The electrochemical behaviour of GO@**3** was investigated using differential pulse voltammetry (DPV) in CH_3_CN (Figures 5 and S16). The immobilized θ-metallacarborane complex GO@**3** exhibits electrochemical features similar to those of its homogeneous counterpart, Na­[**3**]. Two anodic processes are observed at approximately 1.26 and 1.62 V, attributed to the oxidation of pyrene moiety and Co­(IV)/Co­(III) couple, respectively. Compared to Na**[3]**, these potentials are shifted cathodically by 60 mV and anodically by 100 mV. Additionally, a cathodic process at *E*_1/2_ = −1.33 V *vs* SCE corresponds to the Co­(III)/Co­(II) redox couple. This potential is 30 mV more anodic than that of Na­[**3**]. These shifts suggest electronic coupling between the molecular catalyst and the GO support, indicating that the electronic environment of the complex is modulated by partial charge transfer, π–π interactions, and hydrogen bonding with the GO surface.

**Figure 5:**
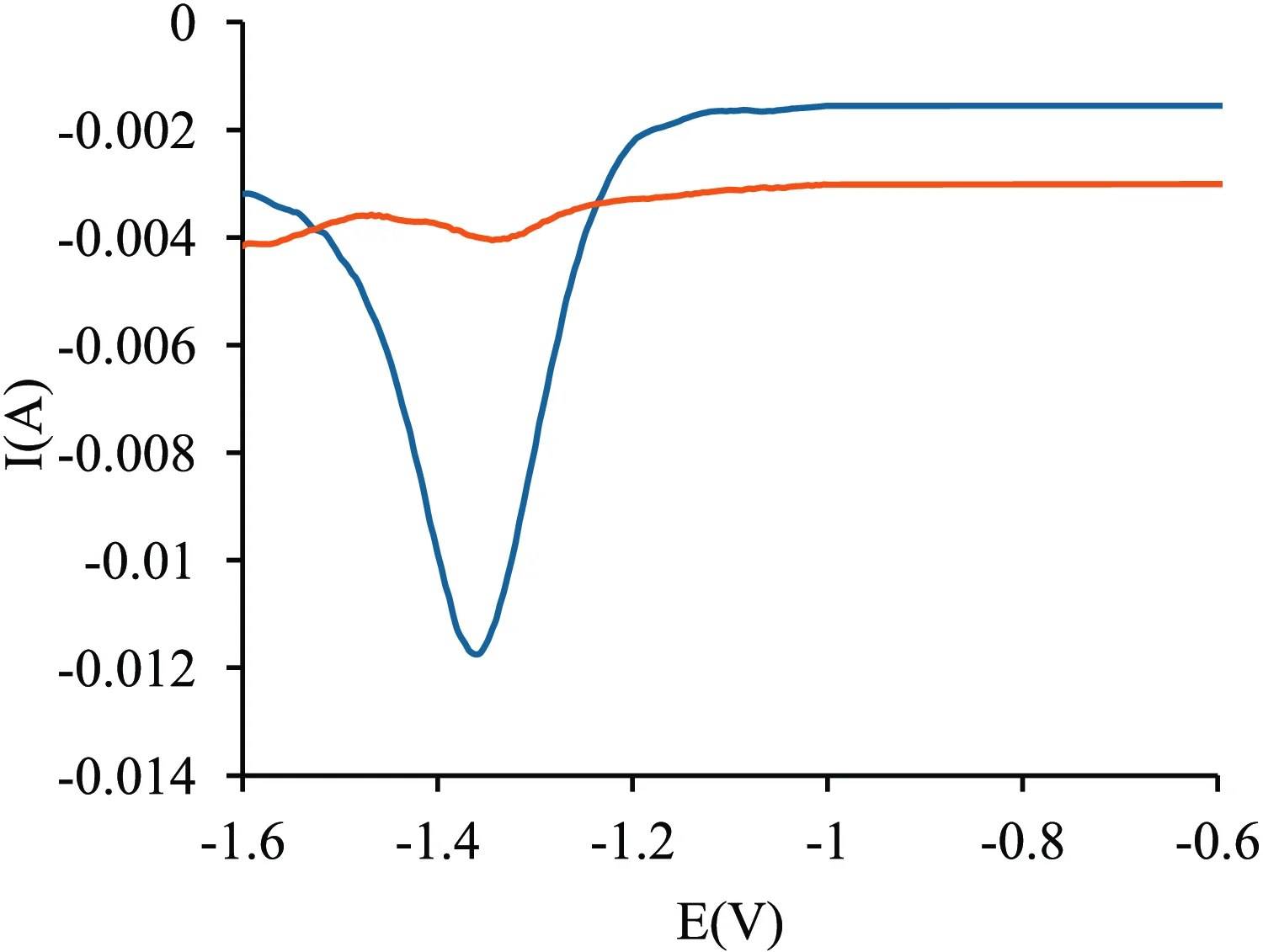
DPV of GO@**3** (orange line) and Na­[**3**] (blue line) corresponding to the Co­(III)/Co­(II) redox couple in CH_3_CN *vs* SCE (reference electrode). Glassy carbon electrode (3 mm diameter) was used as working electrode, platinum wire as auxiliary.

### Optical and Electrochemical Bandgap Analysis of the Photocatalysts

The bandgap is a key property of semiconductor materials used in photocatalysis. It indicates the energy required for an electron to jump from the valence band to the conduction band. When light activates the material, it generates electrons and holes that participate in chemical reactions. The electronic properties of the molecular and hybrid materials were investigated through optical (*E*_g_^op^) and electrochemical bandgap (*E*_g_^ec^) determination to evaluate the effect of immobilization of Na­[**3**] onto graphene oxide. Optical bandgap values (*E*_g_^op^) were estimated from UV–vis absorption spectra using Tauc plots^56,57^ assuming indirect electronic transitions (Figures 6 and S17).

**Figure 6:**
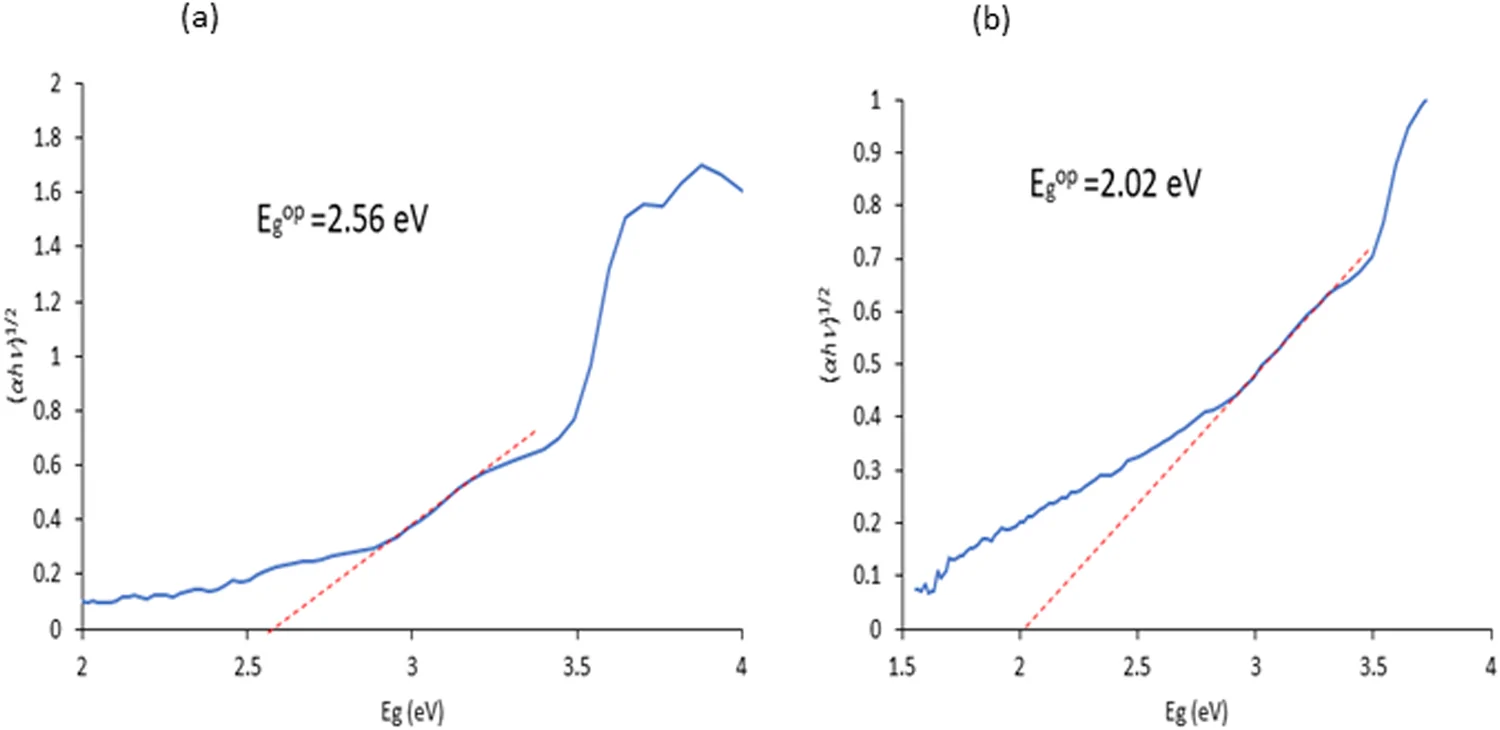
Tauc’s plot to determine the optical bandgap of (a) Na­[**3**] and (b) GO@**3**.

The optical bandgap is defined as the intercept *E*_g_ found from the plotting the equation

$${\left(\alpha h\nu \right)}^{1/n}=A\left(h\nu -E_{g}\right)$$

where α is the absoption coefficient, *h*ν is the incident photon energy (eV), *A* is the proportionality constant, *E*_g_ is the band gap energy, and *n* = 1/2 and 2 for direct and indirect band gap. The Tauc analysis revealed optical bandgap values of 2.56 eV for Na­[**3**], 2.60 eV for [N­(CH_3_)_4_]­[**3**], and 2.02 eV for GO@**3**. The lower bandgap observed for GO@**3** demonstrates that immobilization onto the graphene oxide surface modifies the electronic environment of the molecular complex, promoting a narrower energy separation between the valence and conduction bands.

On the other hand, the electrochemical bandgaps were calculated from DPV measurements, by considering the differences between the HOMO (associated with the first oxidation process) and the LUMO (associated with the first reduction process).^58^ We have calculated the LUMO and HOMO energy values for the compounds using the reduction and oxidation onsets (*E*_red,onset_) and (*E*_oxid,onset_) from DPV (Figures S10 and S16) applying the following equations

$$E_{\mathrm{LUMO}}=-\left(E_{\mathrm{red},\mathrm{onset}}+4.4\right)\;\mathrm{eV}\;\left(E_{\mathrm{red},\mathrm{onset}}\;\mathrm{vs}\;\mathrm{SCE}\right)$$

$$E_{\mathrm{HOMO}}=-\left(E_{\mathrm{oxid},\mathrm{onset}}+4.4\right)\;\mathrm{eV}$$

$$E_{g}=\left(E_{\mathrm{LUMO}}\right)-\left(E_{\mathrm{HOMO}}\right)\;\mathrm{eV}$$

The calculated values were 2.40 eV for Na­[**3**], 2.42 eV for [N­(CH_3_)_4_]­[**3**] and 2.24 eV for GO@**3** (Table S1). Although small differences are observed between optical and electrochemical estimations, both approaches consistently demonstrate that the hybrid material exhibits a reduced bandgap compared with the molecular precursor, which confers more semiconductor character to the hybrid material. This could translate into greater efficiency of solar radiation in photocatalytic processes.

### Photocatalytic Oxidation

Initial photocatalytic alcohol oxidation experiments using Na­[**3**] as catalyst were conducted in aqueous solutions at pH = 7 (adjusted with K_2_CO_3_). Reactions were performed in vials under UVA-I irradiation (λ ∼ 352 nm), at room temperature and atmospheric pressure, using 1-phenylethanol as the model substrate to optimize reaction conditions. Na_2_S_2_O_8_ served as oxidizing agent. To evaluate conversion and yield as a function of time, two different catalyst: substrate: oxidant ratios were tested, 1:1000:2000 and 1:10000:20000 (corresponding to 0.1 and 0.01 mol % of photocatalyst, respectively).

The reaction products were extracted three times with dichloromethane, dried over Na_2_SO_4_ and quantified via NMR analysis. As shown in Figure S18 high yields of acetophenone were achieved after 2 h (89 and 86%, respectively). Yields increased slightly at 4, 6, and 8 h, maintaining high conversion even at low catalyst loadings (96% after 6h and >99% after 8 h). Consequently,the performance of Na­[**3**] was extended to the oxidation of other alcohols at both catalyst loadings (Table 1). While compound [N­(CH_3_)_4_]­[**3**], was insoluble in water, its catalytic activity was evaluated for the oxidation of 1-phenylethanol and 4-methylbenzyl alcohol, over 6 h at 0.1 mol % loading (see Table 1). To confirm the photocatalytic requirement, control experiments were monitored for 8 h; no reaction was observed in the absence of either the catalyst (Na­[**3**] or [N­(CH_3_)_4_]­[**3**]), light, or oxidizing agent.

**Table 1:**
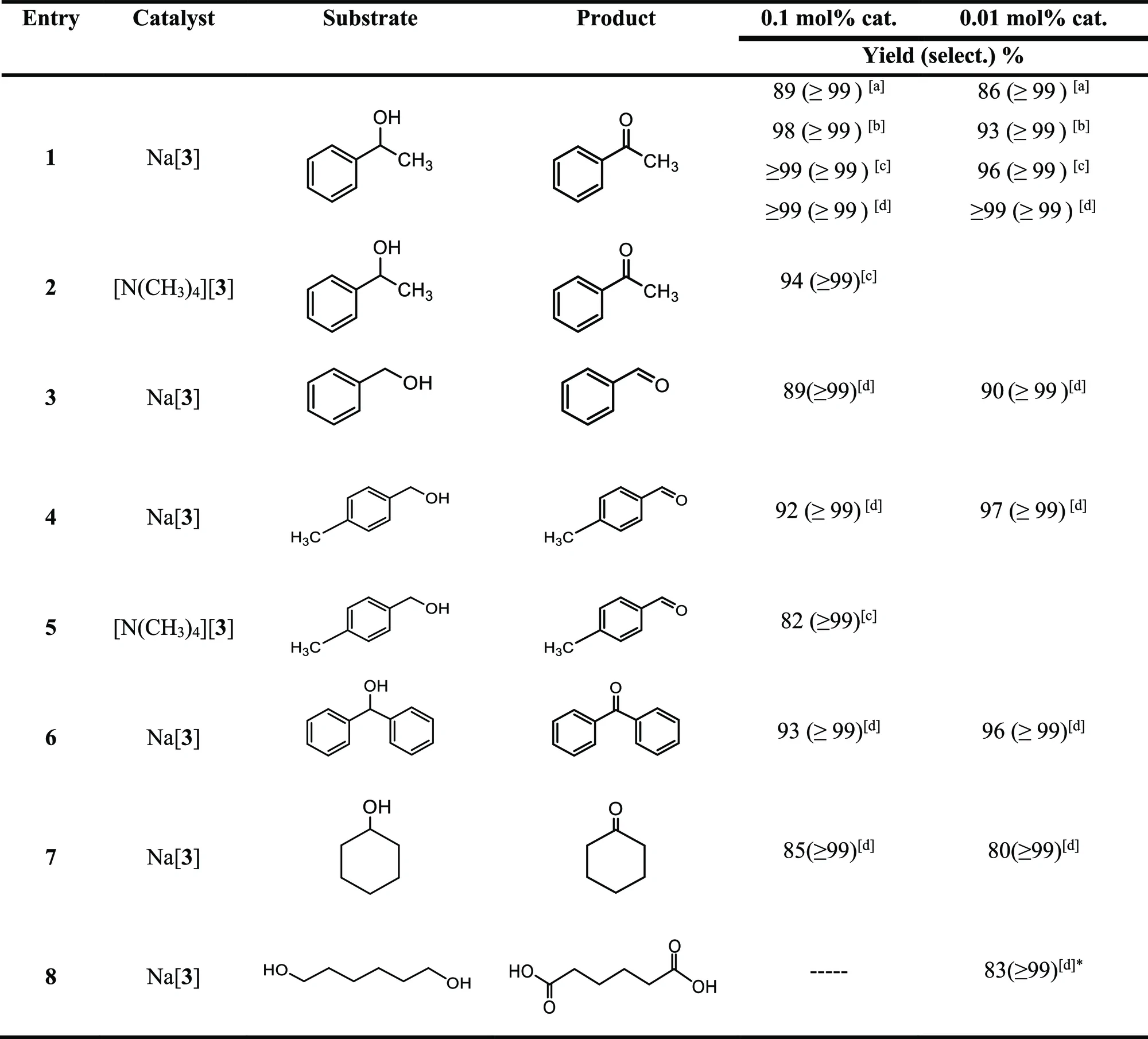
Photocatalytic Oxidation of Alcohols

a After 2 h of reaction.

b After 4 h of reaction.

c After 6 h of reaction.

d After 8 h of reaction. Conditions: Na­[**3**] (0.002 or 0.02 mM), substrate (20 mM), Na_2_S_2_O_8_ (40 mM), 5 mL water at pH = 7; [N­(CH_3_)_4_]­[**3**] (1.3 μmol), substrate (0.26 M), Na_2_S_2_O_8_ (0.53 M), 5 mL water at pH = 7; light irradiation all times using a lamp with λ = 352 nm. Yields of products were determined by NMR spectroscopy.

* Analysis was done on the neat reaction.

Overall, the data in Table 1 demonstrate high conversion values, even at low catalyst loading of 0.01 mol %. This indicates that the potential formation of aggregates at this concentration, as evidenced by our DLS studies, does not hinder the catalytic efficiency of the compound. Regarding substrate scope, the oxidation of primary alcohols, such as benzyl alcohol (entry 3) and 4-methylbenzyl alcohol (entry 4) yielded the corresponding aldehyde as the sole products. Similarly, the oxidation of secondary alcohols, including diphenylmethanol (entry 6) and cyclohexanol (entry 7), resulted in the formation of the respective ketones. In all cases, a remarkable selectivity of >99% was achieved.

The photooxidation of the 1,6-hexanediol (entry 8) resulted in the formation of the diacid in good yield. In general, aromatic alcohols exhibited higher reactivity compared to their aliphatic counterparts. Compound [N­(CH_3_)_4_]­[**3**] demonstrated strong performance in the oxidation of 1-phenylethanol and 4-methylbenzyl alcohols after 6 h; notably, the conversion of 1-phenyletanol was only slightly lower than that achieved with the sodium analogue, Na­[**3**]. Given its insolubility in water, we confirmed the heterogeneous nature of this molecular catalyst through recyclability tests. As shown in Figure S19, [N­(CH_3_)_4_]­[**3**] was successfully reused at least six cycles without any loss of catalytic activity, likely due to the high chemical stability of the compound, which remains intact throughout successive runs.

From a sustainable catalysis perspective, minimizing catalyst loading is essential to reduce costs and waste generation. Consequently, anchoring active complexes onto appropriate supports facilitates the development of green photocatalysts with enhanced stability, and environmental compatibility. In this context, we investigated the activity of the hybrid heterogeneous system GO@**3** for the photooxidation of several alcohols in water. Based on the amount of complex anchored to the GO support, a catalyst loading of 0.01 mol % was employed. Reactions were conducted at room temperature and atmospheric pressure under UVA-I irradiation (λ ∼ 352 nm) for 4 and 6 h. Products were extracted with dichloromethane, dried over Na_2_SO_4_ and quantified via ^1^H NMR spectroscopy. Blank tests conducted with bare GO yielded less than 10% conversion after 6 h of reaction. Similarly, the exclusion of either the light, the photocatalyst GO@**3**, or the oxidant led to no substantial oxidation, verifying that each component is essential for the process to occur.

As summarized in Table 2, the heterogeneous GO@**3** system exhibits excellent performance in the photooxidation of both aromatic and aliphatic alcohols within 4 to 6 h. It is noteworthy that GO@**3** achieves comparable performance in only 6 h (Table 2, entries 3, 5, and 7) to those obtained with homogeneous Na­[**3**] after 8 h (Table 1, entries 4, 6, and 7). This significant enhancement underscores the role of the graphene oxide support. This synergistic effect likely arises from the heterogenization of the complex, which optimizes the exposure of active sites to both UVA-I irradiation and reactants while facilitating more efficient charge separation. Furthermore, the observed shifts in bandgap values suggest an electronic modulation of the catalyst’s environment, potentially enhancing light-harvesting capacity and, consequently, overall catalytic efficiency. This behaviour is consistent with recent reports showing that immobilization of cobaltabis­(dicarbollide) photocatalysts onto solid supports (graphene oxide^23^ and silica-based chiral nanohelices^41^) enhances catalyst stability and recyclability while preserving high photocatalytic activity. Whereas silica-based chiral nanohelices provide a confined environment enabling enantioselective photooxidation,^41^ the graphene oxide support employed here promotes efficient substrate accessibility and electron transfer, leading to efficient photocatalytic oxidation under longer-wavelength UVA irradiation.

**Table 2:**
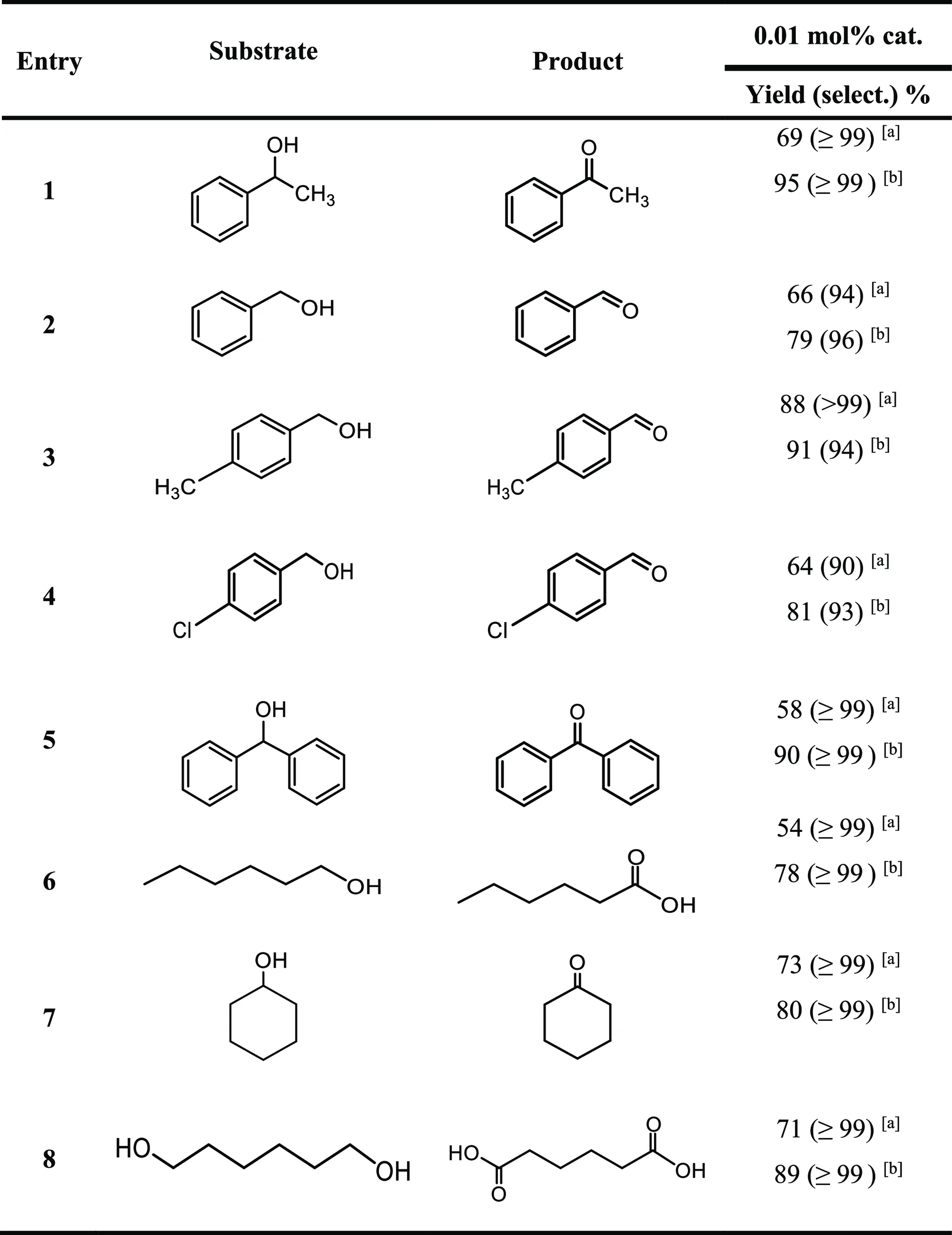
Photocatalytic Oxidation of Alcohols Using GO@**3** as Catalyst

a After 4 h of reaction.

b After 6 h of reaction. Conditions: **GO@3** (0.1 μmol), substrate (1 mmol), Na_2_S_2_O_8_ (2 mmol), 5 mL water solution at pH = 7; light irradiation all times using a lamp with λ = 352 nm. Yields of products were determined by NMR spectroscopy.

The photooxidation of primary benzylic alcohols (entries 2–4) yielded the corresponding aldehydes as the major products, alongside minor amounts of acids, achieving high selectivity (90–99%). For secondary aromatic alcohols, the respective ketones were obtained as the sole products, with excellent selectivity (>99%; entries 1, 5 and 7). Regarding aliphatic substrates, 1-hexanol was efficiently oxidized to hexanoic acid with a 78% yield after 6 h, (entry 6), while cyclohexanol was converted to cyclohexanone with an 80% yield over the same period (entry 7). Furthermore, the oxidation of 1,6 hexanediol successfully afforded the corresponding diacid in a high yield of 89% (entry 8).

To verify that the observed photocatalytic activity is exclusively attributed to the GO- supported species rather than the leaching of θ-metallacarborane into the reaction medium, a UV–vis spectrum of the supernatant was recorded following the oxidation of 1-phenylethanol. The absence of any characteristic absorption bands in the 210–350 nm range confirms that the complex remains firmly anchored to the support with no detectable leaching, thereby establishing the truly heterogeneous nature of the catalyst.

Based on the photocatalytic outcomes, a comprehensive reaction mechanism driven by synergetic photogenerated electron transfer is proposed (Figure 7). Under light irradiation, the cobaltabisdicarbollide core of the Na[3] moieties serves as the primary light-harvesting center. The highly delocalized, three-dimensional aromatic framework of the dicarbollide capping ligands stabilizes the frontier molecular orbitals. Upon photon absorption, an electron from the HOMO of [Co­(III)]^−^, is promoted to the LUMO, generating the transient photoexcited species [CoIII]*^–^. To suppress rapid charge recombination, this high-energy electron is efficiently injected into the adjacent graphene oxide (GO) framework, which acts as an effective electron mediator and sink. The delocalized electrons within the GO sheets subsequently induce the reductive cleavage of persulfate S_2_O_8_^2–^ molecules, generating highly reactive sulfate radical anions. Concurrently, this single-electron transfer (SET) pathway results in the oxidative quenching of the complex, formally converting the cobalt center to its tetravalent state to form the highly oxidizing [Co­(IV)] species. This transient Co­(IV) state physically constitutes the highly reactive photogenerated holes (h^+^) capable of driving substrate activation.

**Figure 7:**
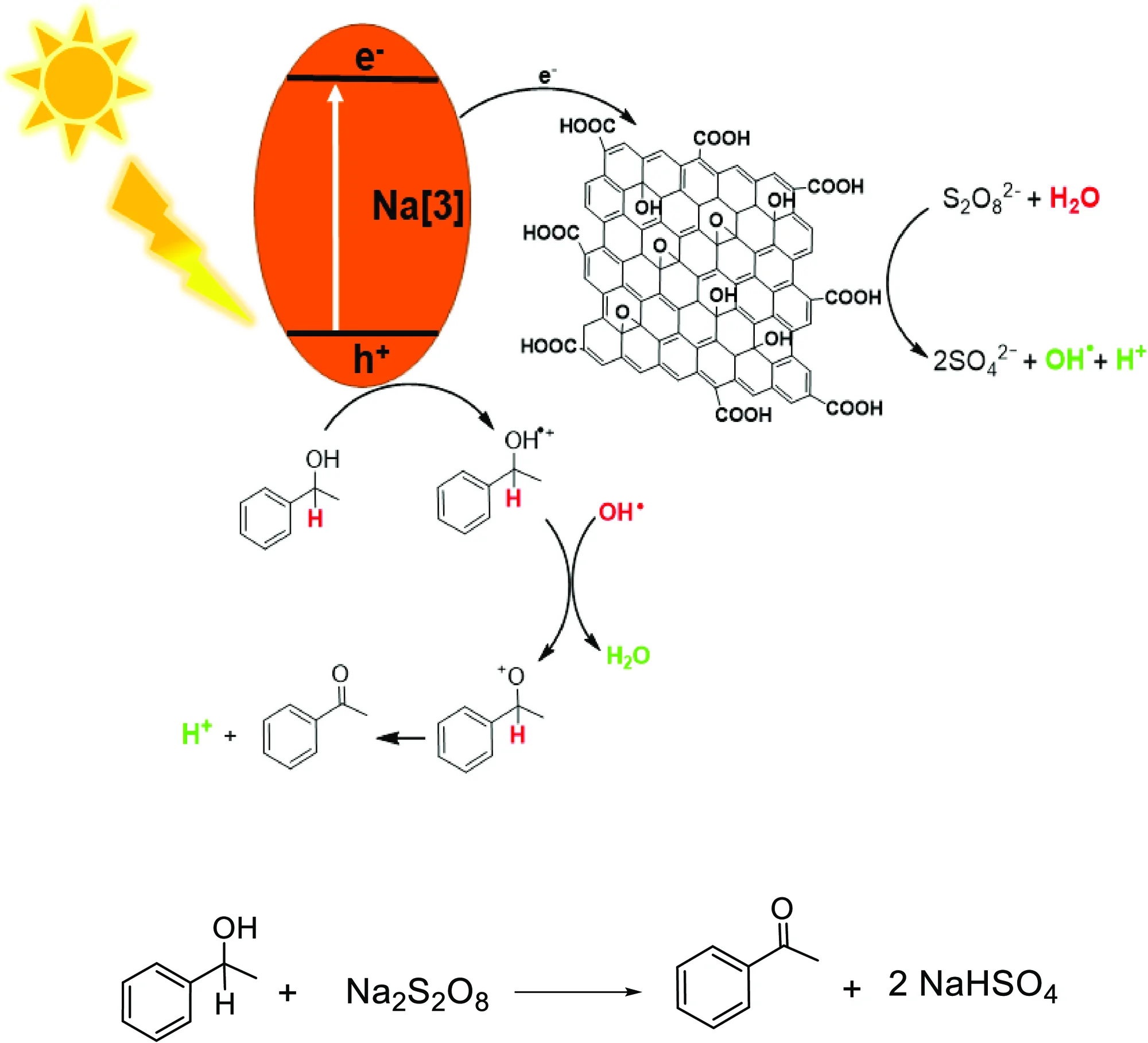
Proposed mechanism for the photocatalytic oxidation of alcohols using GO@**3** as catalyst. Some reagents are shown in red and in green. They indicate that they are produced and consumed during the reaction. At the bottom is the overall reaction that has taken place.

Subsequently, the strong oxidant Co­(IV) abstracts an electron from the organic substrate (alcohol), returning the cobalt center to its original stable Co­(III) ground state to close the photoredox cycle. The resulting alcohol radical/carbocation intermediates then react via proton abstraction mediated by the photogenerated hydroxyl radicals. These pathways lead to the selective formation of carbonyl compounds or, in the case of unhindered linear primary alcohols, undergoes sequential hydration to yield the corresponding carboxylic acids. Finally, the remaining protons and sodium cations are rationally combined with the reduced sulfur species to generate stable NaHSO_4_ the primary reaction by-product.

As mentioned earlier, evaluating the applicability of a photocatalyst, requires that it be reusable while maintaining consistent performance over multiple cycles. Beyond activity, a photocatalyst’s practical applicability depends on its robustness and reusability over multiple cycles. Consequently, the recyclability of GO@**3** was evaluated in the photooxidation of 4-methylbenzyl alcohol (4 h) and cyclohexanol (6 h) using a catalyst loading of 0.01 mol %. As illustrated in Figure 8 the catalyst maintained consistent performance.

**Figure 8:**
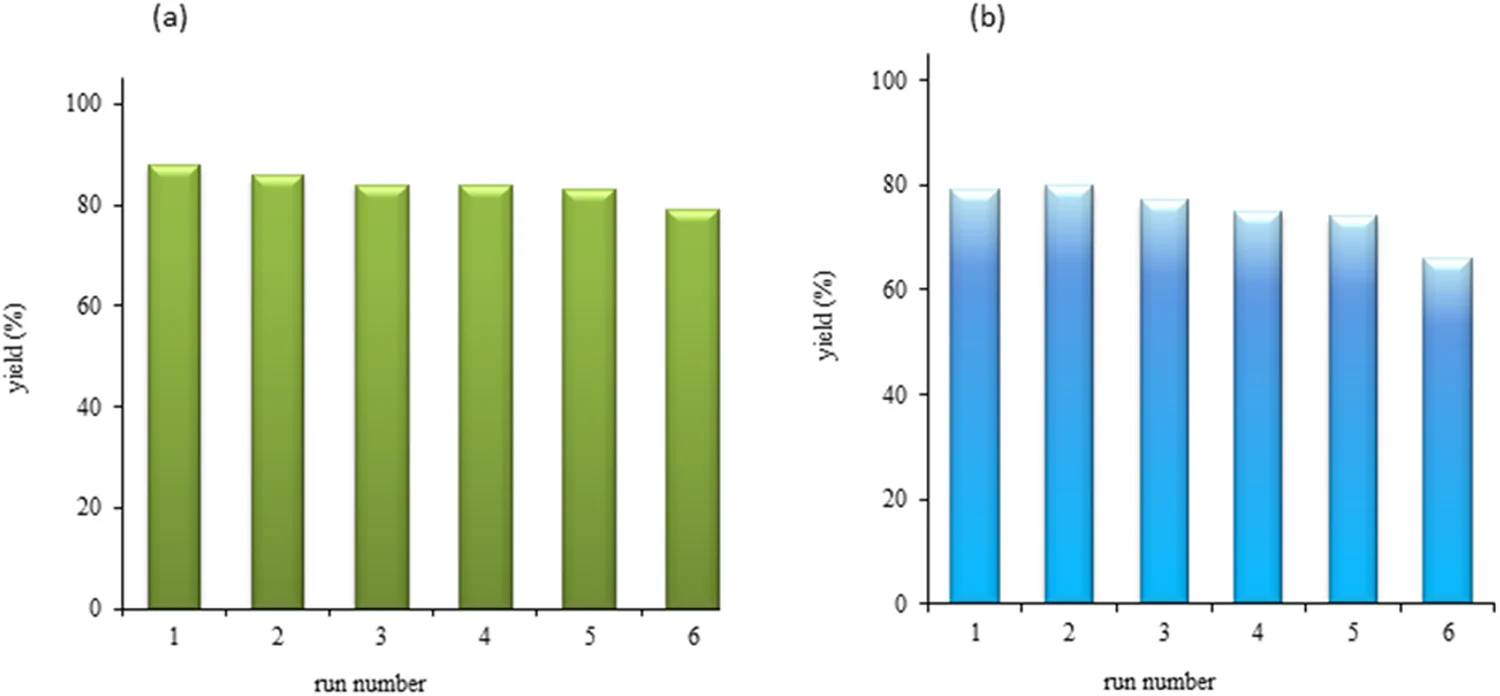
Yield values obtained throughout six successive reuses of GO@**3** in the photooxidation of (a) 4-methylbenzyl alcohol and (b) cyclohexanol. Conditions: GO@**3** (1x10^–3^ mmol), substrate (1 mmol), Na_2_S_2_O_8_ (2 mmol), water solution at pH = 7 (5 mL); light irradiation using a lamp with λ = 352 nm; 4 h per run for methylbenzyl alcohol and 6 h per run for cyclohexanol.

In both instances, the results demonstrate that the hybrid material can be recycled for at least six cycles, with negligible metal leaching confirmed by UV-vis analysis of the filtrates, while maintaining consistent conversion and selectivity, indicating that no significant structural or compositional changes occur during reuse. Notably, selectivity remained above 99% in all runs, with only a minor decline in cyclohexanol conversion observed by the sixth reuse. After six cycles, the system achieved overall turnover numbers (TON) of 50,400 for the photooxidation of 4-methylbenzyl alcohol and 45,100 for cyclohexanol. These exceptionally high TON values underscore the remarkable stability of the hybrid system, which we attribute to the favorable non-covalent interactions between the GO support and the θ-metallacarborane. The slight decrease in catalytic activity observed over time is likely due to the mechanical loss of the catalyst during the recovery and separation steps rather than chemical degradation. The robust structural and physical integrity of the catalyst under the reported reaction conditions is directly supported by TEM analysis. A detailed comparison of the TEM images recorded before photocatalysis (Figures 9 and S13) and after six consecutive cycles (Figure S20) reveals no discernible changes in surface morphology, particle distribution, or structural framework.

**Figure 9:**
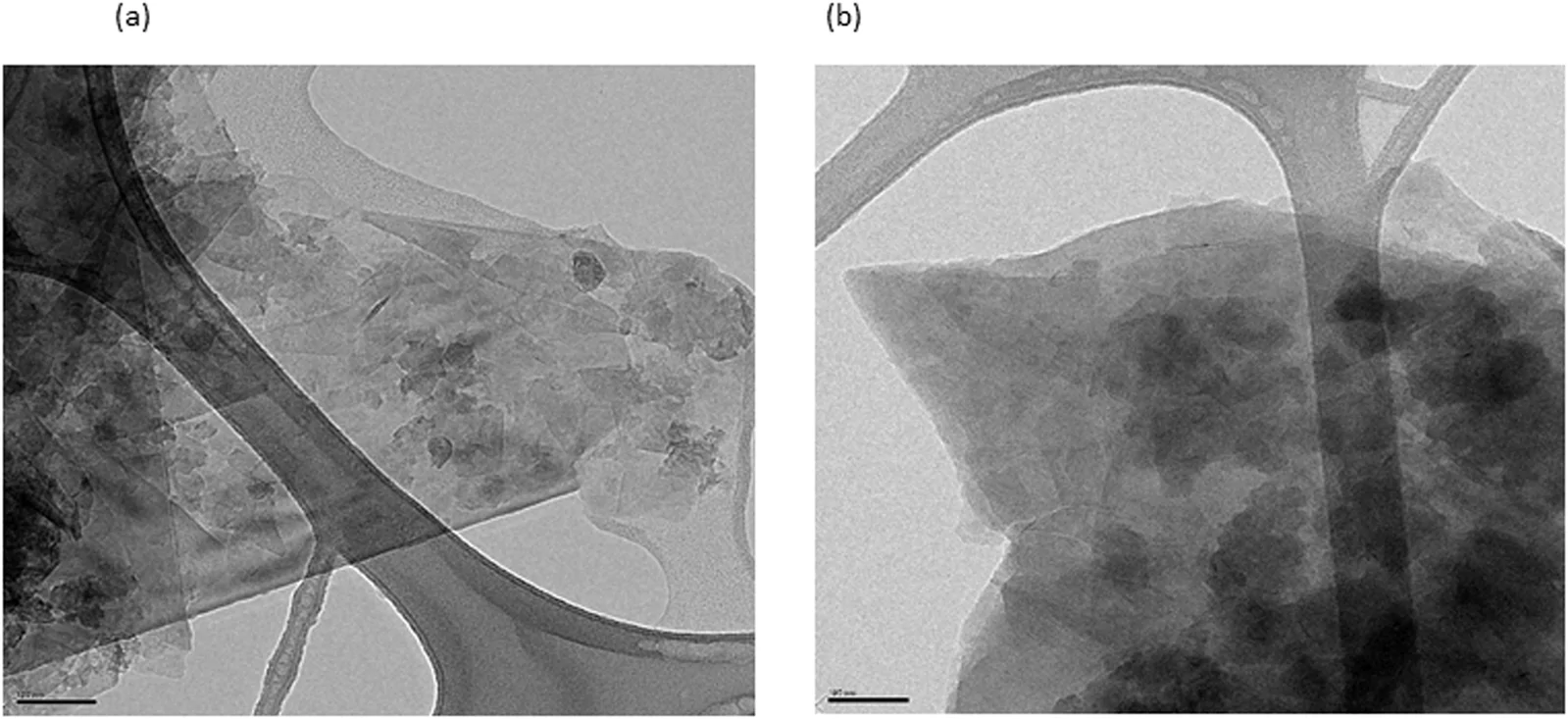
TEM images of GO@**3** (the bars indicate a length of 100 nm), (a) before and (b) after six reuses in the photooxidation of 4-methylbenzyl alcohol.

While these TEM data demonstrate excellent morphological stability and rule out degradation processes such as sintering or support collapse, subtle variations in the local coordination environment or oxidation states of the active centers cannot be entirely excluded without advanced surface spectroscopies (e.g., XPS or XAS). Nevertheless, the combined recycling, leaching, and electron microscopy data confirm that the heterogeneous catalyst exhibits outstanding operational and structural robustness.

Concerning the robustness of the cobaltabisdicarbollide we wish to emphasize its extraordinary stability to radiation, which has proven remarkably resistant to the harsh radiation fields (γ, α, β) and chemically aggressive media (concentrated HNO_3_) encountered in nuclear waste reprocessing, and has been used successfully for the selective extraction of ^137^Cs and ^90^Sr.^59–61^

## Conclusion

This research focused on the development of novel heterogeneous photocatalytic systems based on the functionalization of cobaltabis­(dicarbollide) anions ([o-COSAN]^−^) with a pyrene moiety, exploring the feasibility of immobilizing these homogeneous photoredox catalysts onto graphene oxide (GO) support. In alignment with the principles of green chemistry, our goal was to evaluate their performance in the photocatalytic oxidation of alcohols in water while ensuring the recyclability of the heterogeneous system. The molecular complex Na­[**3**] was synthesized via the ring-opening reaction of the cyclic oxonium **2** with pyreneacetic acid, while [N­(CH_3_)_4_]­[**3**] was subsequently prepared from Na­[**3**] through cation metathesis using tetramethylammonium salts. The hybrid system GO@**3** was obtained by anchoring Na­[**3**] onto GO support in an aqueous medium, a process driven by π–π interactions facilitated by the pyrene group of the functionalized [*o*-COSAN]^−^. Comprehensive spectroscopic and electrochemical characterizations of both the homogeneous and heterogeneous materials confirm the successful assembly of the hybrid systems containing the cobaltabis­(dicarbollide) unit.

The electrochemical behavior of GO@3 exhibited a shift in redox potentials relative to Na­[**3**], suggesting a degree of electronic coupling between the molecular catalyst and the GO support. While the homogeneous Na­[**3**] displayed high catalytic efficiency and total selectivity aqueous alcohols photooxidation, even at low loading (0.01 mol %), The insoluble analogue [N­(CH_3_)_4_]­[**3**], functioned as a heterogeneous catalyst, successfully reused over six cycles.

Notably, the performance of GO@**3** at shorter reaction times was comparable to that of Na­[**3**] after 8 h. The graphene oxide support significantly enhances catalytic performance; GO@**3** consistently outperformed the unsupported complex. This improvement is likely attributed to the support’s ability to narrow the bandgap, facilitate electron transfer, and promote charge separation, thereby preventing recombination of photogenerated electron-hole pairs. Additionally, heterogenization on GO may prevent catalyst deactivation and modulate the electronic environment, enhancing light absorption and overall activity.

Recyclability studies on the photooxidation of 4-methylbenzyl alcohol and cyclohexanol demonstrated the exceptional stability of GO@**3**, which maintaining high conversion and selectivity (>99%) across six consecutive runs. The system achieved remarkably high cumulative turnover numbers (TON > 45,000) underscoring its robustness.

In conclusion, this study establishes the fundamental photocatalytic performance of earth-abundant cobaltabis­(dicarbollide) complexes. It is important to address that the current system operates under UVA-I excitation, which represents only a small fraction of the solar spectrum and constitutes a practical limitation for direct solar energy conversion. While UV-driven artificial light sources find established niches in specific industrial sectors (such as targeted water purification or high-energy chemical synthesis), maximizing solar utilization remains the ultimate goal for sustainable chemistry. In this context, visible-light photocatalysis represents a paramount and rapidly evolving field with emerging large-scale applications. To overcome the limitations of UV activation and align this system with solar applications, the modular nature of these metallacarborane derivatives offers a promising pathway. Ongoing work in our laboratory is focused on anchoring these complexes onto visible-light-responsive supports, such as graphitic carbon nitride. This strategy aims to “red-shift” the operational window into the visible region, thereby enhancing the practical relevance, efficiency, and environmental sustainability of these earth-abundant catalytic materials.

## Supplementary Material



## Notes

Spectroscopic and electrochemical characterization of the homogeneous and heterogenous photocatalysts and additional catalytic data (PDF)
