# Supplementary material for: Pyrene-Functionalized o‑COSAN Noncovalently Anchored onto Graphene Oxide for Advanced Photocatalytic Alcohol Oxidation
Source: ACS Omega. 2026 Sep 11;11(37):56419–33. doi: 10.1021/acsomega.6c07608 (PMC13613445; doi:10.1021/acsomega.6c07608)
Supplement: Supplementary file 1 [file ao6c07608_si_001.pdf]

SUPPORTING INFORMATION

Pyrene-Functionalized *o*-COSAN Non-Covalently Anchored onto Graphene Oxide for Advanced Photocatalytic Alcohol Oxidation

Syrine Affès,[a] Nabila Mimouni,[a] Isabel Guerrero,[b] Rosario Núñez,[b] Ahlem Kabadou, [c] Clara Viñas,[b] Francesc Teixidor,[b] and Isabel Romero [a]**

[a]Departament de Química and Serveis Tècnics de Recerca, Universitat de Girona, C/ M. Aurèlia Capmany, 69, E-17003 Girona, Spain.

[b]Institut de Ciencia de Materials de Barcelona (ICMAB-CSIC), Campus de la UAB, 08193 Bellaterra (Cerdanyola del Vallès), Barcelona, Spain

[c]Laboratoire des Sciences des Matériaux et d'Environnement, Faculté des Sciences, Université de Sfax, Sfax 3029, Tunisie

Corresponding authors

*E-mail for I.R.: marisa.romero@udg.edu;
*E-mail for F.T.: teixidor@icmab.es

Table S1. Energies values of HOMO and LUMO and electrochemical band gaps of complexes.

Figure S1. IR spectra of a) Na[3,3'-Co(8-O(CH_2CH_2O)$_2$C(O)-(CH_2-pyrene)(1,2-$C_2B_9H_{10}$)-1',2'-$C_2B_9H_{11}$],Na[**3**] and b) [N(CH_3)$_4$][3,3'-Co(8-O(CH_2CH_2O)$_2$C(O)(CH_2-pyrene-1,2-$C_2B_9H_{10}$)(1',2'-$C_2B_9H_{11}$)], [N(CH_3)$_4$][**4**] compounds. c) IR spectra of **2** (orange), pyreneacetic acid (grey) and Na[**3**] (blue).

Figure S2. NMR spectra of Na[**3**] (400 MHz, acetone-d_6): a) ^{1}H-NMR; b) ^{1}H{^{11}B}-NMR; c) ^{13}C{^{1}H}-NMR; d)^{11}B-NMR; e)^{11}B {^{1}H}-NMR; f)^{1}H-^{13}C HSQC.

Figure S3. NMR spectra of [N(CH_3)$_4$][**3**](400 MHz, acetone-d_6): a) ^{1}H-NMR; b) ^{1}H{^{11}B}-NMR; c) ^{13}C{^{1}H}-NMR; d)^{11}B-NMR; e)^{11}B {^{1}H}-NMR; f)^{1}H-^{13}C HSQC.

Figure S4. UV-*vis* spectra of a) Na[**3**] (0.010 mM); b) [N(CH_3)$_4$][**3**] (0.015 mM) in CH_3CN and c) spectral superposition of [3,3'-Co(8-$C_4H_8O_2$-1,2-$C_2B_9H_{10}$)(1',2'-$C_2B_9H_{11}$)], **2,** (green) and [Na][**3**] in CH_2Cl_2 (0.080 mM) (blue).

Figure S5. ESI-MS of Na[**3**] in a) negative ion mode and b) positive ion mode.

Figure S6. ESI-MS of [N(CH_3)$_4$][**3**] in a) negative ion mode and b) positive ion mode.

Figure S7. TGA of Na[**3**].

Figure S8. CVs for a) 1-pyreneacetic acid and b) compound **2** in CH_3CN + 0.1 M [*n*-Bu$_4$N][PF$_6$] (TBAPF$_6$) *vs* SCE, showing the anodic and cathodic regions.

Figure S9. CVs for [N(CH_3)$_4$][**3**] compound in CH_3CN + 0.1 M [*n*-Bu$_4$N][PF$_6$] (TBAPF$_6$) *vs* SCE, showing the anodic and cathodic regions.

Figure S10. DPVs for a) Na[**3**] and b) [N(CH_3)$_4$][**3**] compounds in CH_3CN + 0.1 M [*n*-Bu$_4$N][PF$_6$] (TBAPF$_6$) *vs* SCE, showing the anodic and cathodic regions.

Figure S11. Size distribution graphs for a) Na[**3**] and b) [N(CH_3)$_4$][**3**] at concentration of 0.020 mM in water:acetone (1:1) and for c) Na[**3**] and d) [N(CH_3)$_4$][**3**] at concentration of 0.002 mM

Figure S12. UV-*vis* spectrum of Na[**3**] (0.1mM) in water/acetone (blue line); UV-visible of the initial solution after 24 h in contact with GO (orange line).

Figure S13. a) TEM images of GO@**3**, a) 200 nm b)100 nm and c) 20nm.

Figure S14. EDX spectra of GO@**3**.

Figure S15. a) XPS spectra of GO@**3**; Magnification of the XPS spectrum of GO@**3** in b) the C1s c) the O1s and d) B 1s region.

Figure S16. DPVs for GO@**3** in CH_3CN + 0.1 M [*n*-Bu$_4$N][PF$_6$] (TBAPF$_6$) *vs* SCE, showing the anodic (a) and cathodic (b) regions.

Figure S17. Tauc's plot to determine the optical bandgap of $[N(CH_3)_4]$[**3**].

Figure S18. Plot of yield as a function of time for the photoredox catalysis of 1-phenylethanol.

Figure S19. Yield values obtained throughout a number of successive reuses of the catalyst $[N(CH_3)_4]$**[3]** in the photooxidation of 1-phenylethanol. Conditions: $[N(CH_3)_4]$[**3**] (1.3 μmol), substrate (0.26 M), $Na_2S_2O_8$ (0.53 M), 5 mL water at pH=7, light irradiation using a lamp with λ = 352 nm, 6 h per run.

Figure S20. a) TEM images of GO@**3** after six reuses.

Figure S21. Representative NMR spectra of the obtained products in the photooxidation of alcohols.

Figure S22. NMR spectra of the pure products in the photooxidation of alcohols.

Table S1. Energies values of HOMO and LUMO and electrochemical band gaps of complexes.

**compound**	**E_{HOMO} (eV)**	**E_{LUMO}(eV)**	E_g^{op} **(eV)**	E_g^{ec} **(eV)**
Na[**3**]	-5.55	-3.15	2.56	2.40
[$N(CH_3)_4$][**3**]	-5.55	-3.13	2.65	2.42
GO@**3**	-5.43	-3.19	2.02	2.24

Figure S1. IR spectra of a) Na[3,3'-Co(8-O$(CH_2CH_2O)_2$C(O)-(CH_2-pyrene)(1,2-$C_2B_9H_{10}$)**-**1',2'-$C_2B_9H_{11}$],Na[**3**] and b) [N$(CH_3)_4$][3,3'-Co(8-O$(CH_2CH_2O)_2$C(O)(CH_2-pyrene-1,2-$C_2B_9H_{10}$)(1',2'-$C_2B_9H_{11}$)], [N$(CH_3)_4$][**4**] compounds. c) IR spectra of **2** (orange), pyreneacetic acid (grey) and Na[**3**] (blue).

The IR spectra of Na[**3**] and [N$(CH_3)_4$][**3**] (Figure S1a,b) exhibit a characteristic $C_{cluster}$-H ($\upsilon_{Ccluster\text{-}H}$) stretching vibration at 3035 cm^{-1}, while the vibrations around 2950-2800 cm^{-1} are assigned to $\upsilon_{C\text{-}H}$ stretching modes of both the pyrene group and the methylene units from the ring-opened dioxane fragment. For both compounds, the B-H ($\upsilon_{B\text{-}H}$) stretching vibrations of the dicarbollide clusters appear as intense bands centered around 2545 cm^{-1}. Significant bands are also observed around 1670-1680 cm^{-1} corresponding to the $\upsilon_{C=O}$ stretching of the carboxylate group. Additionally, the absorption bands in the 1250-900 cm^{-1} range are attributed to the OCH_2- stretching of the polyether chain, while the band at 845 cm^{-1}, is characteristic of the pyrene moiety. Figure 1Sc, provides a comparison of the IR spectra of the starting materials and the functionalized complex Na [**3**].

a)

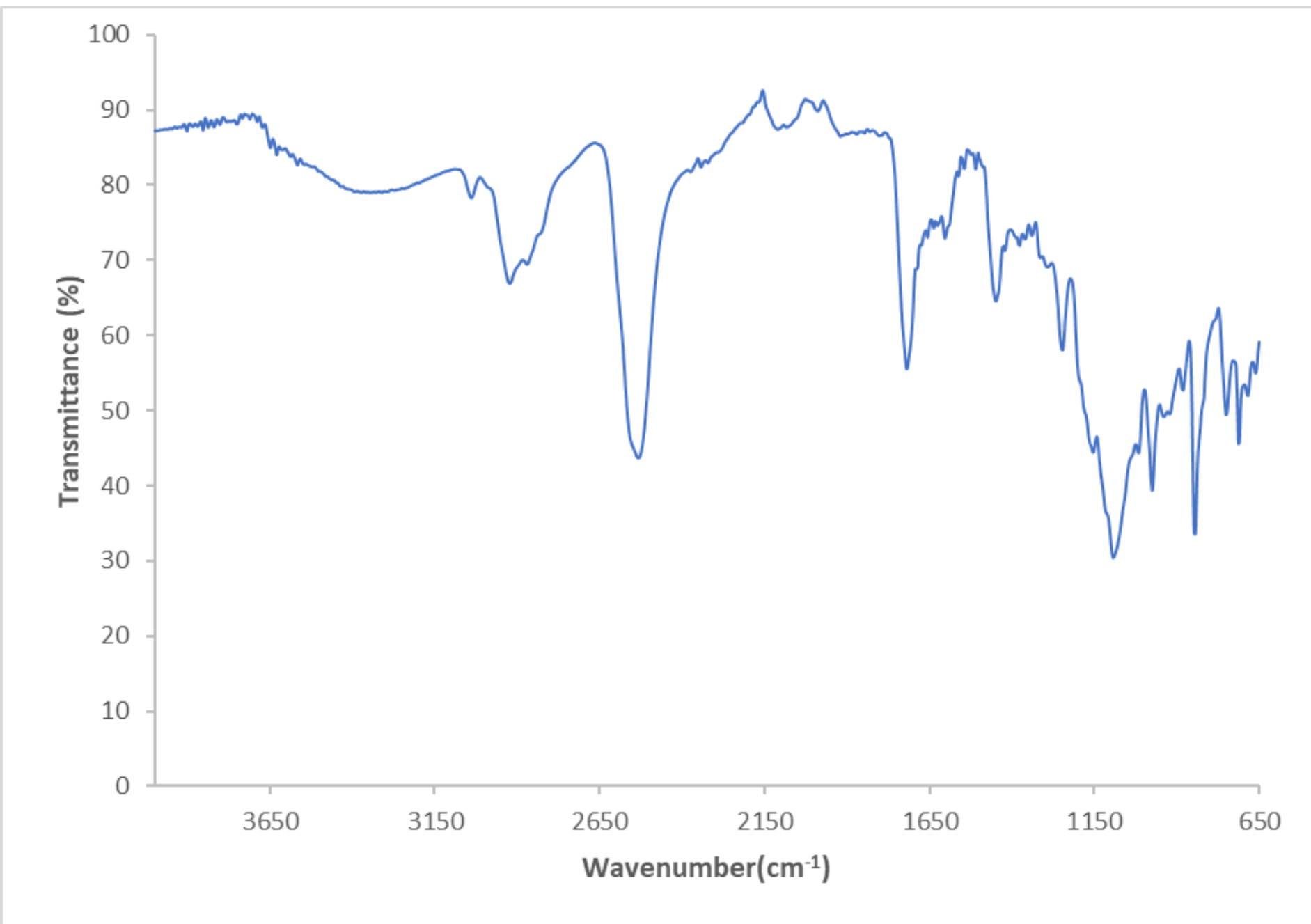

b)

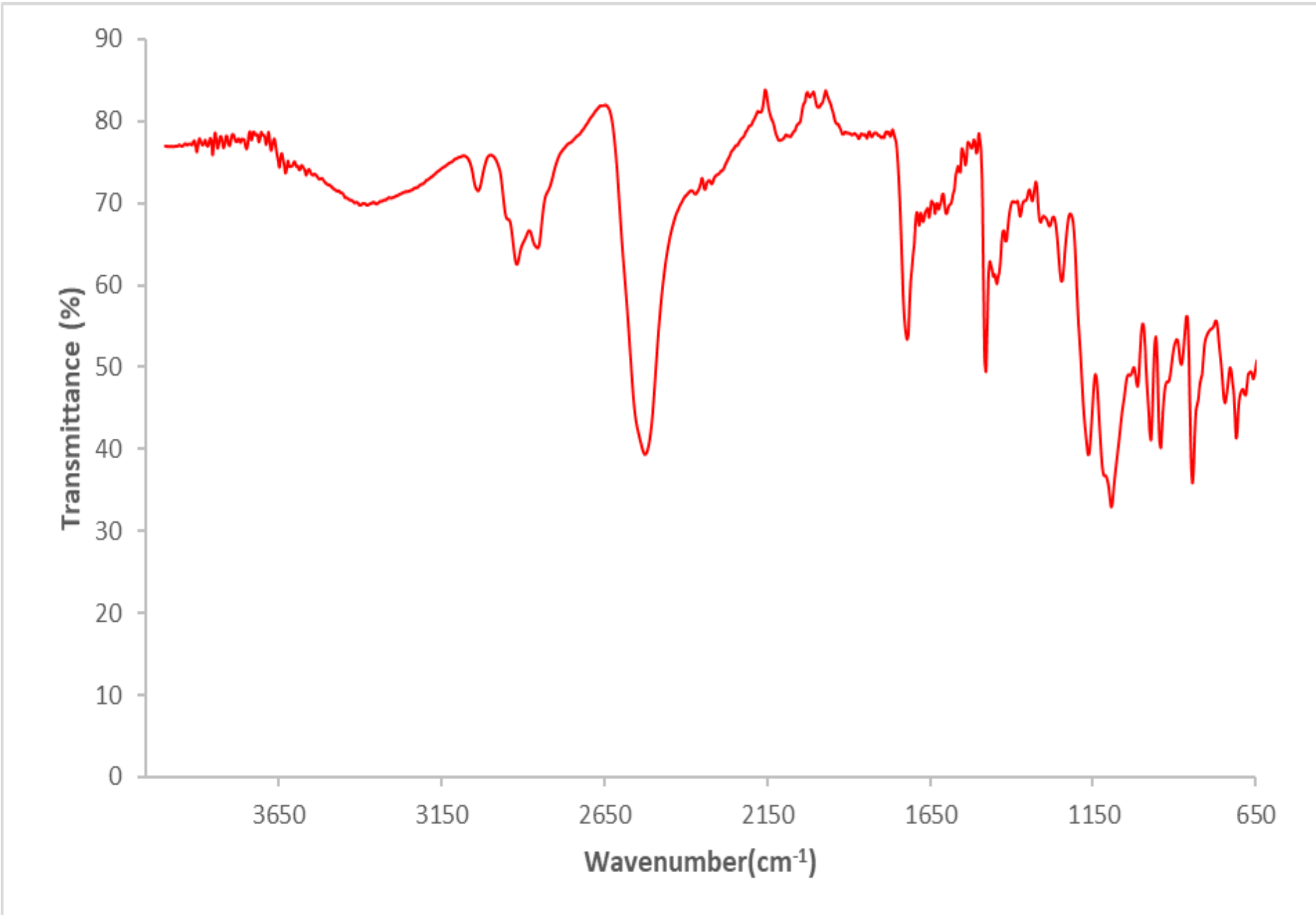

Transmittance (%)
0
10
20
30
40
50
60
70
80
90
3650
3150
2650
2150
1650
1150
650
Wavenumber(cm-1)

c)

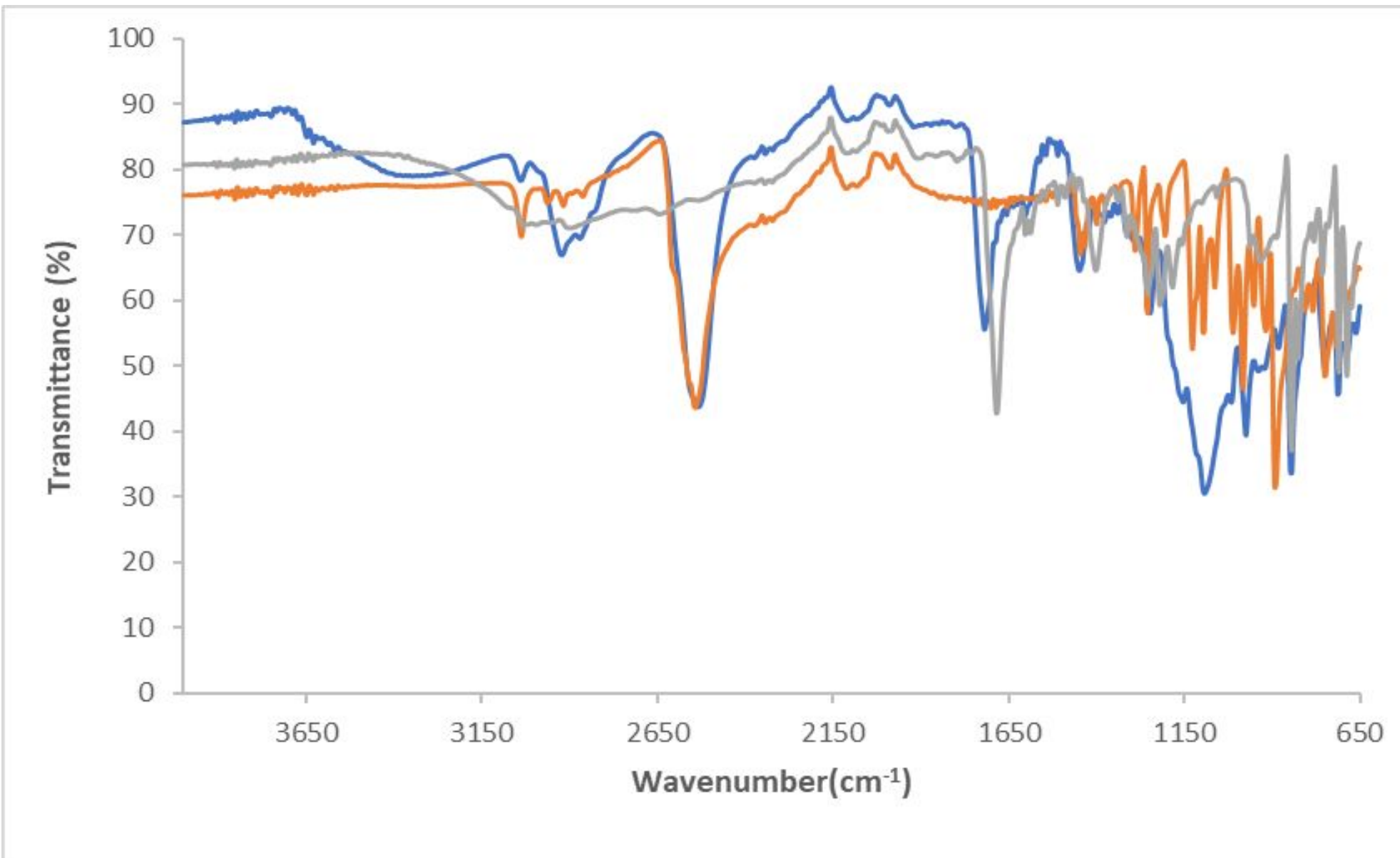

Transmittance (%)
0
10
20
30
40
50
60
70
80
90
100
3650
3150
2650
2150
1650
1150
650
Wavenumber(cm-1)

Figure S2. NMR spectra of Na[**3**] (400 MHz, acetone-d_6): a) ^{1}H-NMR; b) ^{1}H{^{11}B}-NMR; c) ^{13}C{^{1}H}-NMR; d)^{11}B ; e)^{11}B {^{1}H}-NMR; f)^{1}H-^{13}C HSQC

a)

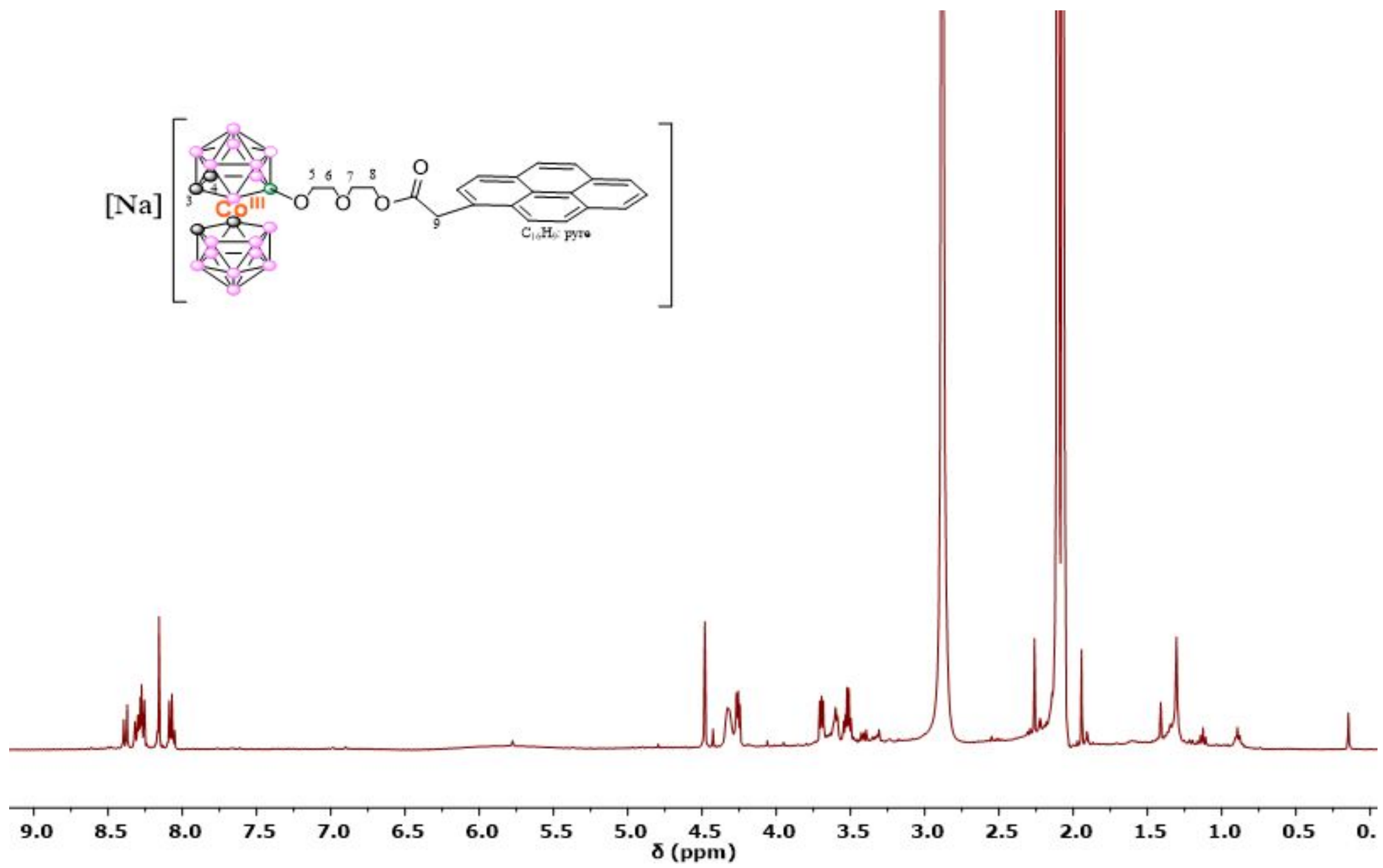

b)

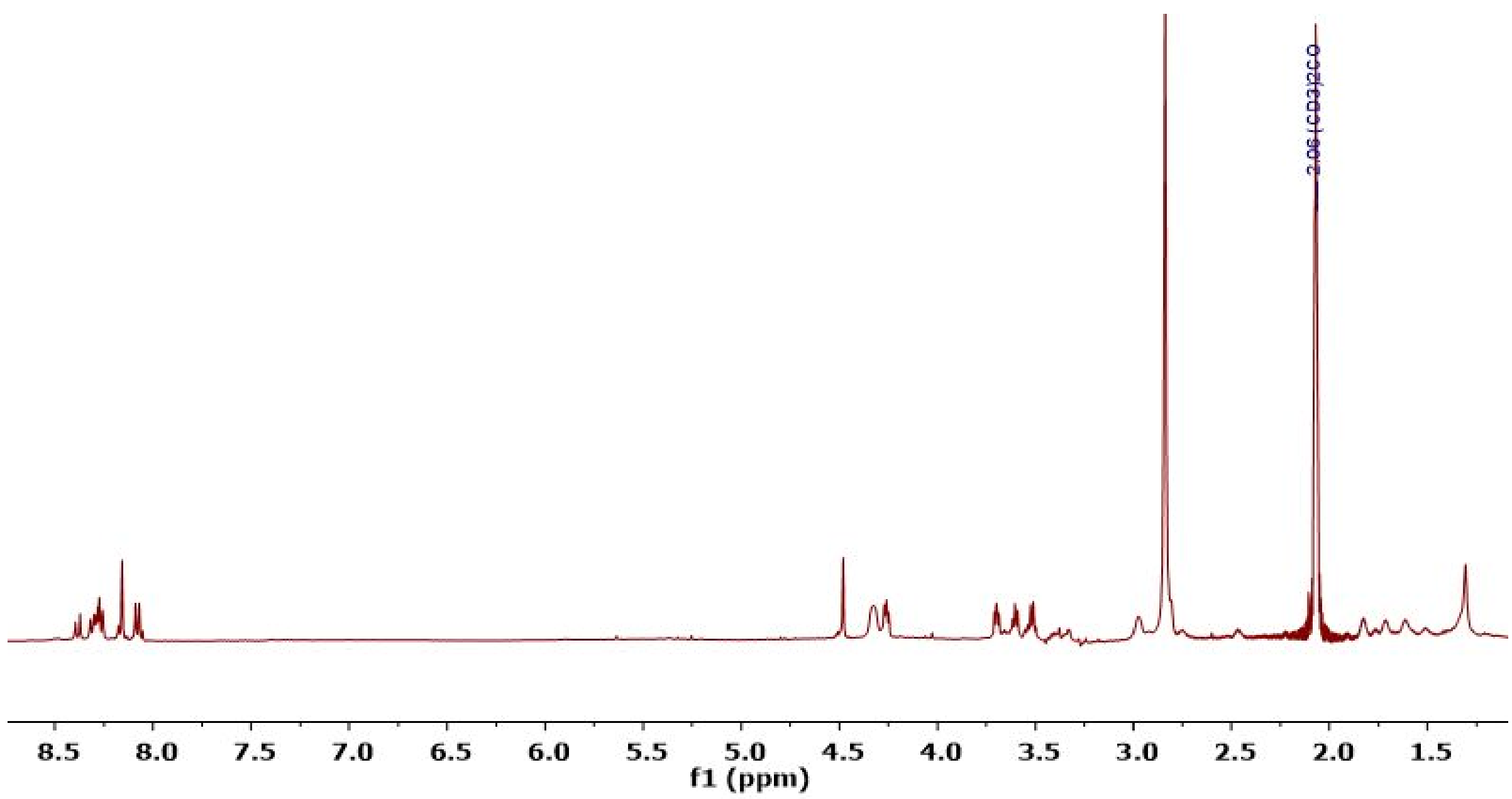

c)

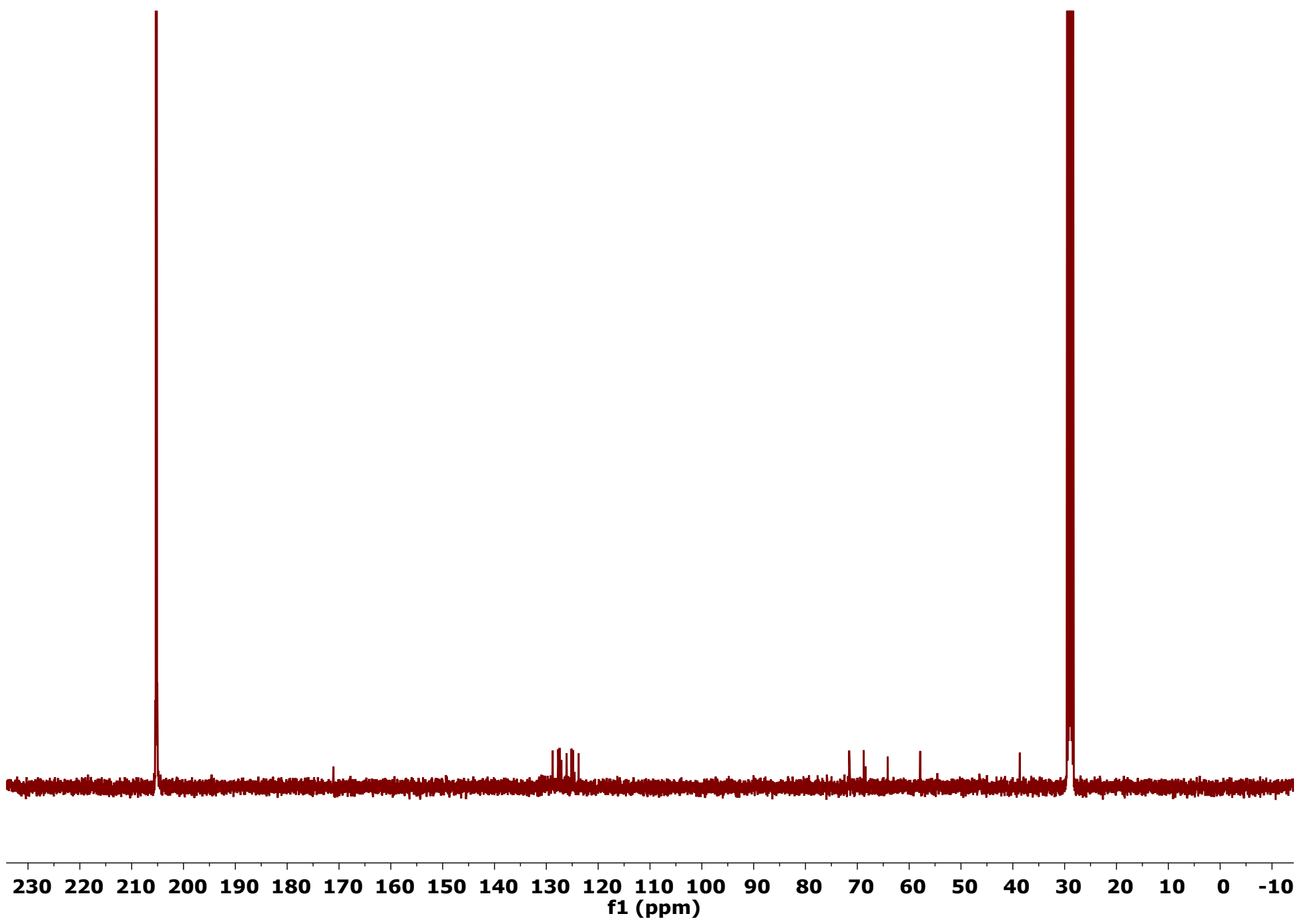
230 220 210 200 190 180 170 160 150 140 130 120 110 100 90 80 70 60 50 40 30 20 10 0 -10
f1 (ppm)

d)

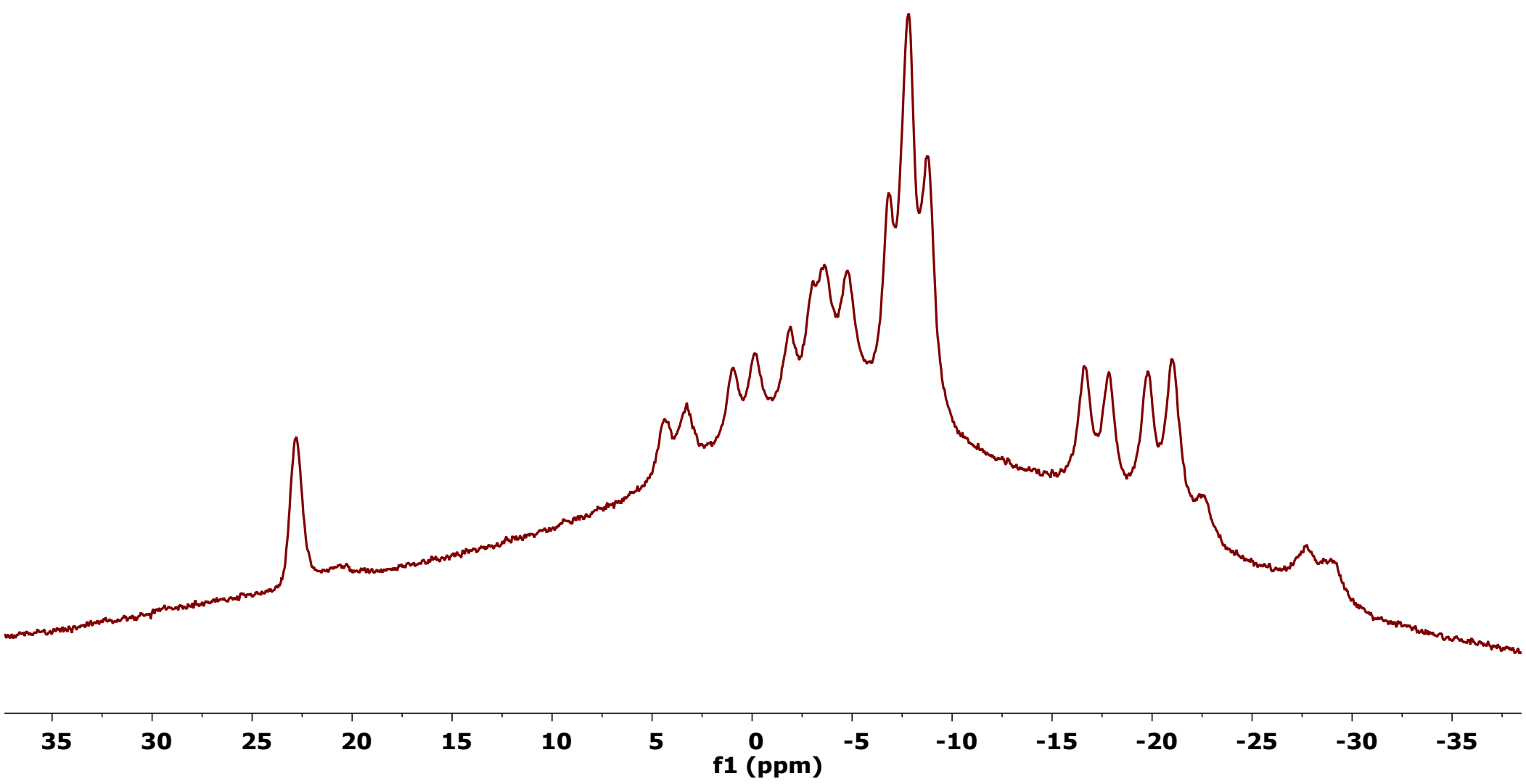
35 30 25 20 15 10 5 0 -5 -10 -15 -20 -25 -30 -35
f1 (ppm)

e)

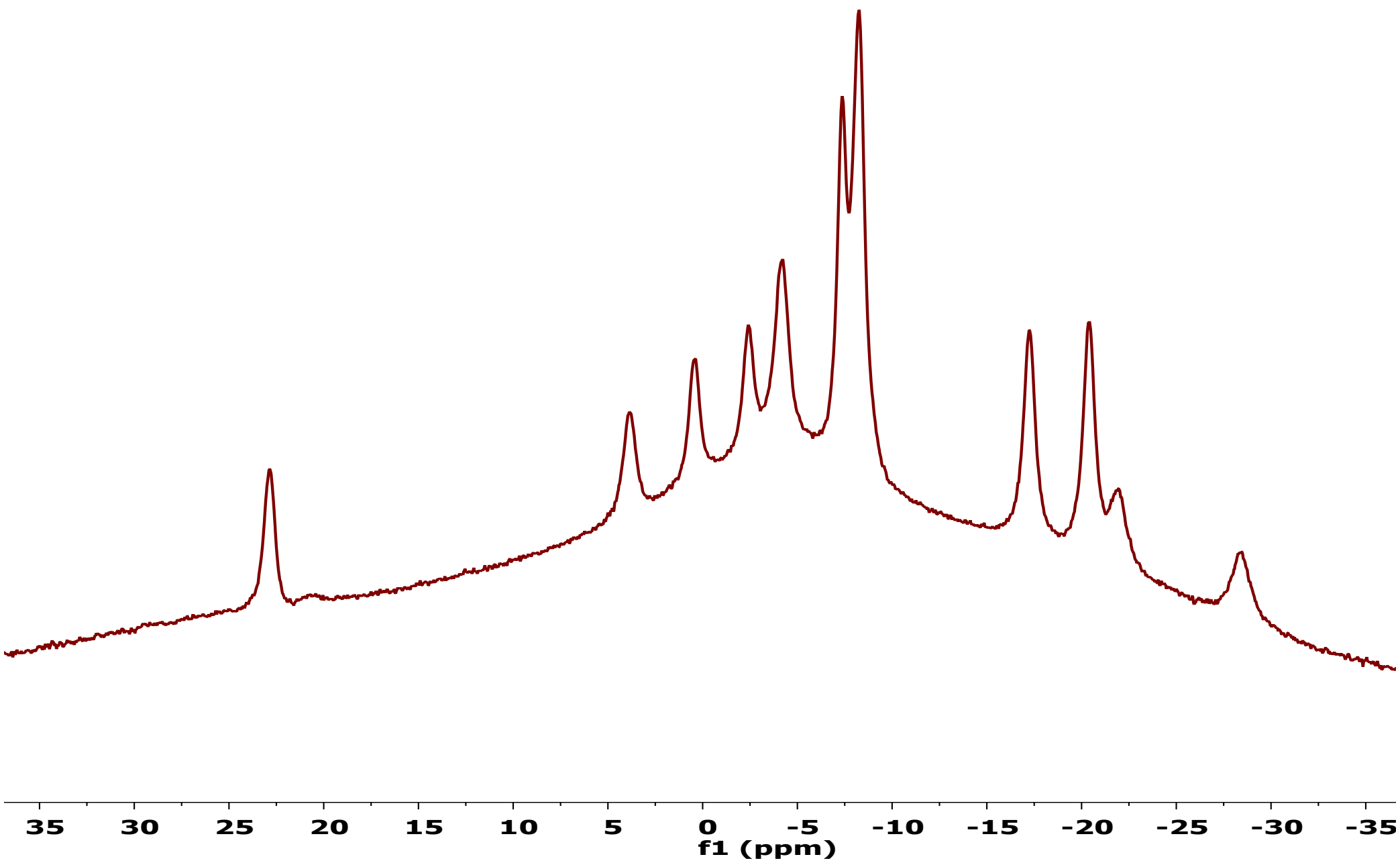

35 30 25 20 15 10 5 0 -5 -10 -15 -20 -25 -30 -35
f1 (ppm)

f)

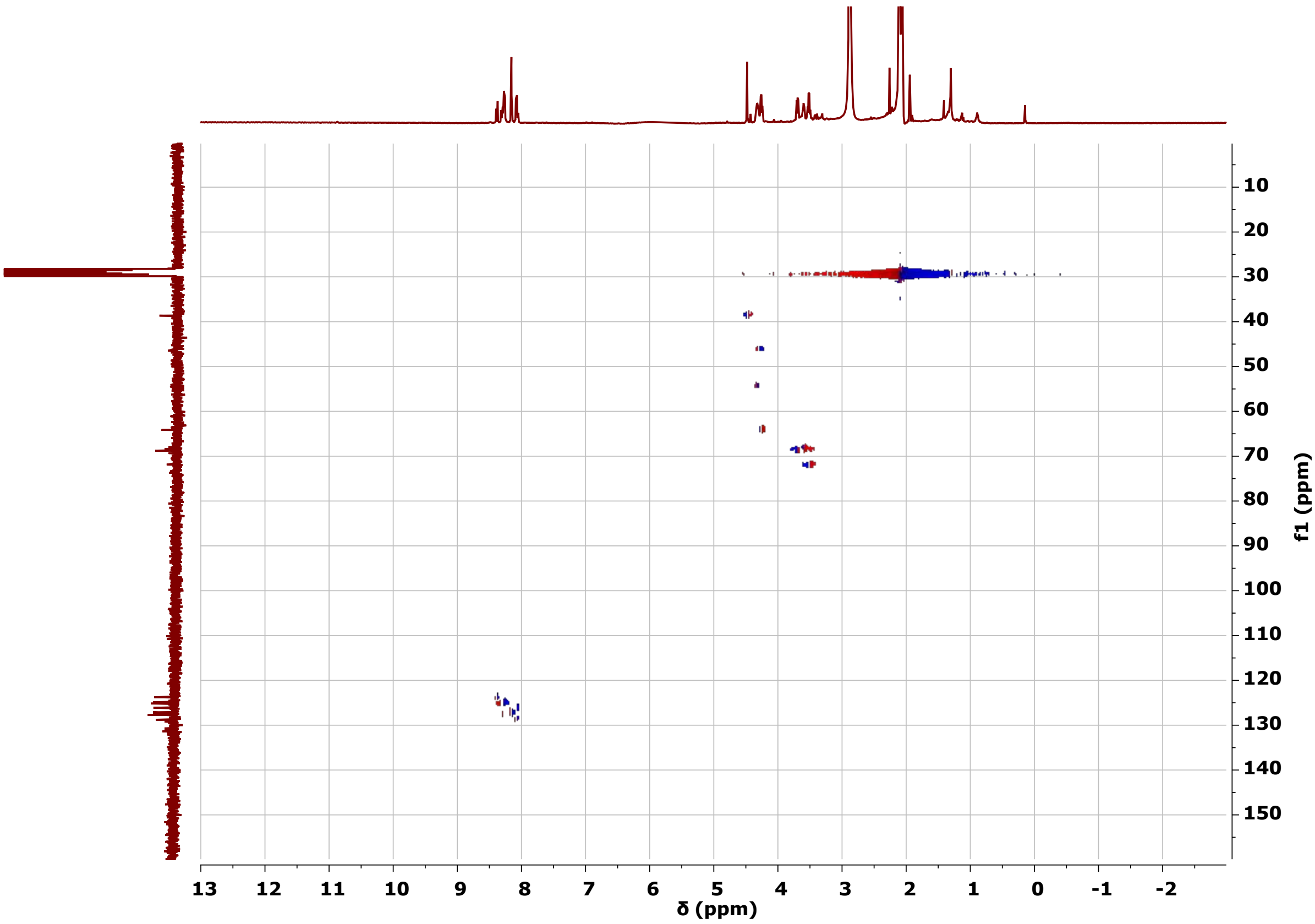

13 12 11 10 9 8 7 6 5 4 3 2 1 0 -1 -2
δ (ppm)
10 20 30 40 50 60 70 80 90 100 110 120 130 140 150
f1 (ppm)

Figure S3. NMR spectra of $[N(CH_3)_4][\mathbf{3}]$ (400 MHz, acetone-d_6): a) ^{1}H-NMR; b) ^{1}H{^{11}B}-NMR; c) ^{13}C{^{1}H}-NMR; d)^{11}B-NMR; e)^{11}B {^{1}H}-NMR; f)^{1}H-^{13}C HSQC.

a)

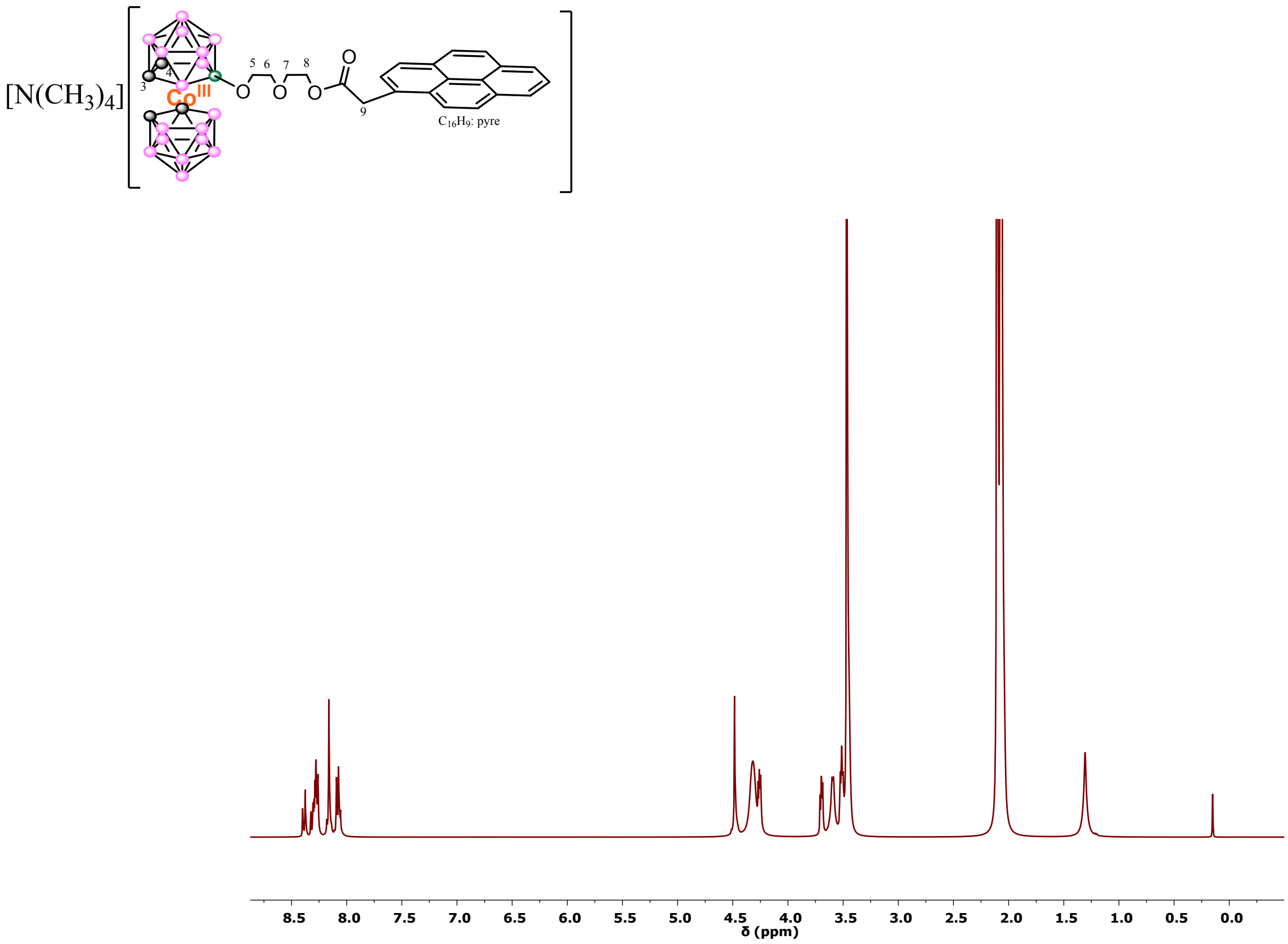

b)

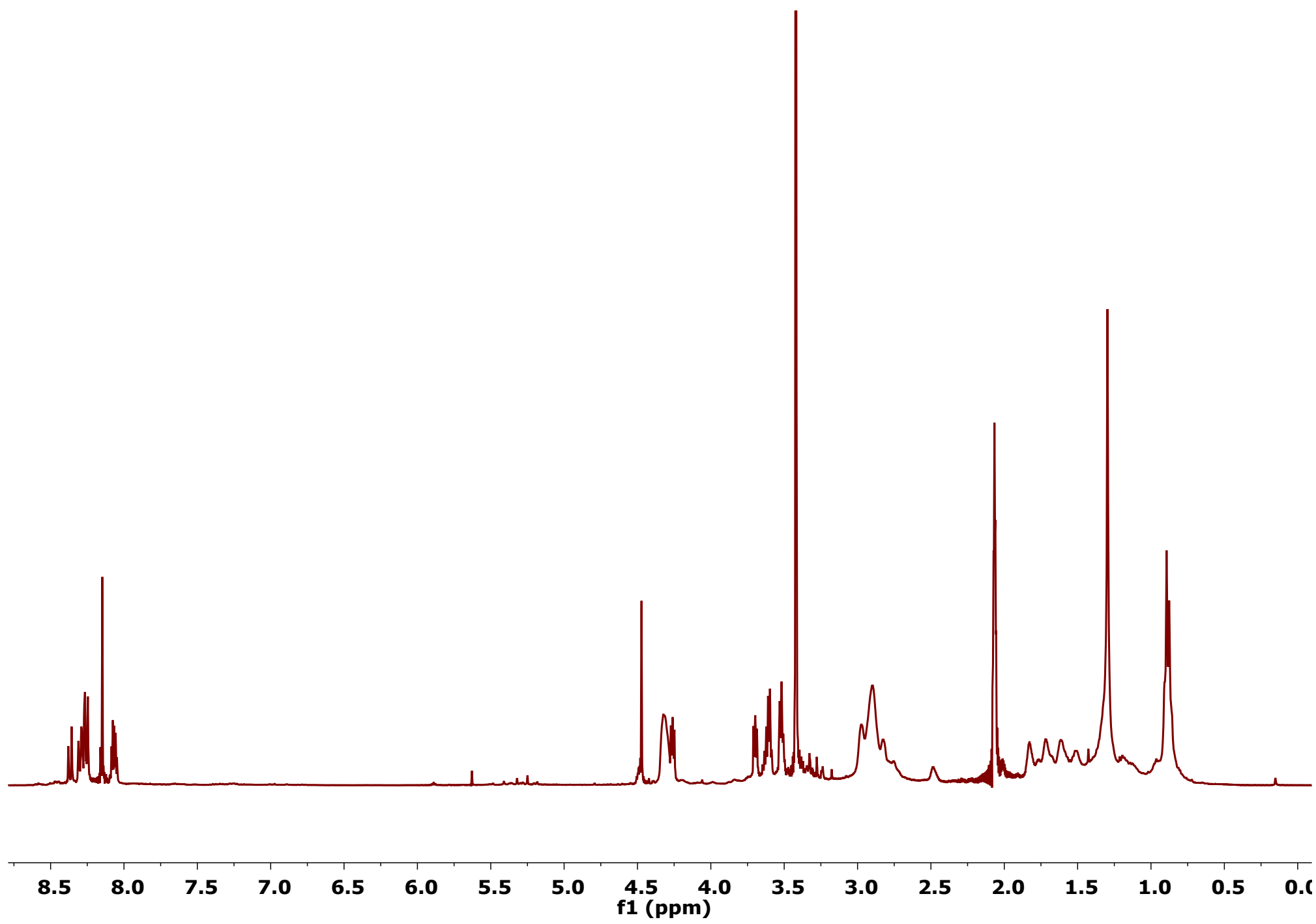

c)

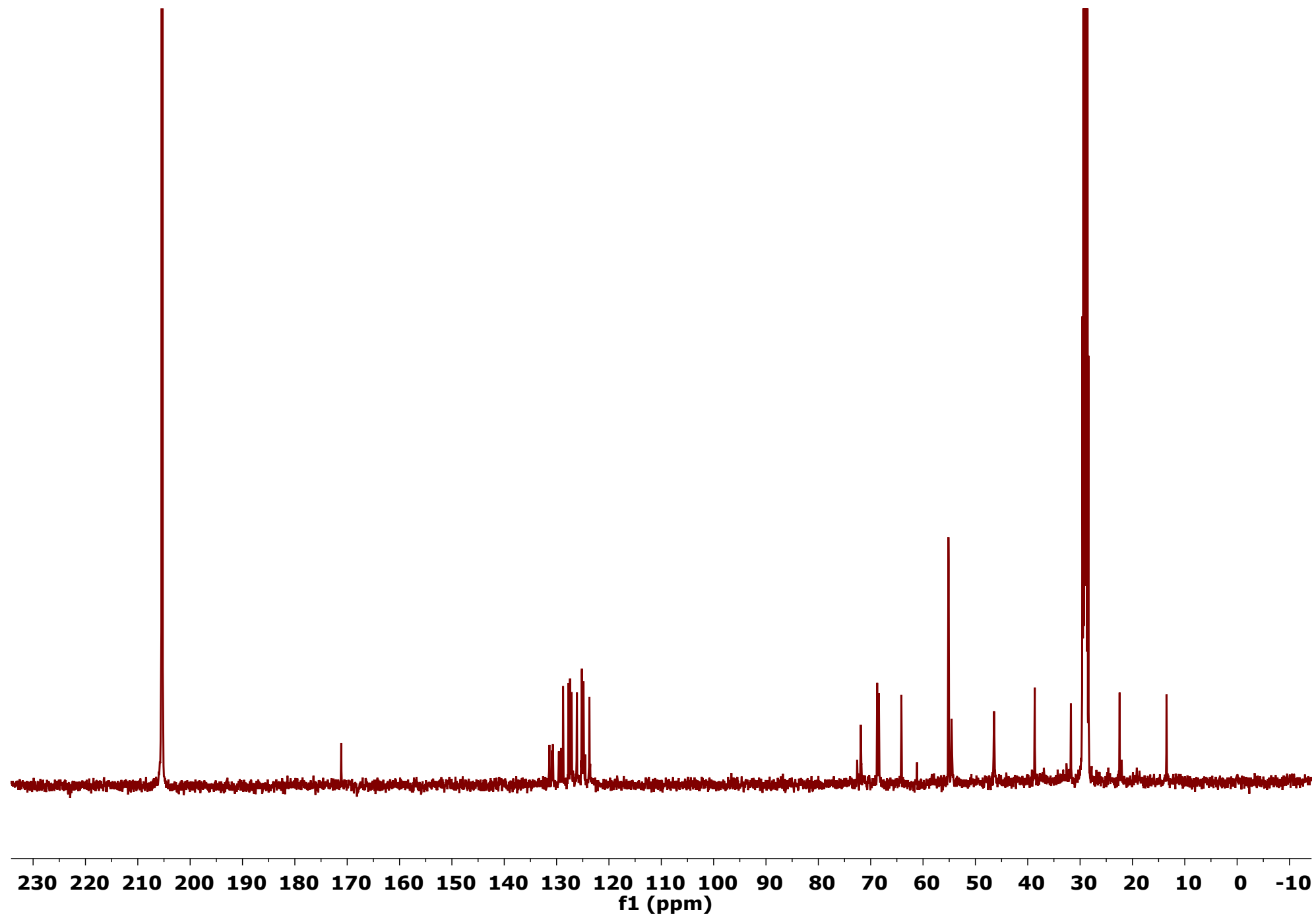
230 220 210 200 190 180 170 160 150 140 130 120 110 100 90 80 70 60 50 40 30 20 10 0 -10
f1 (ppm)

d)

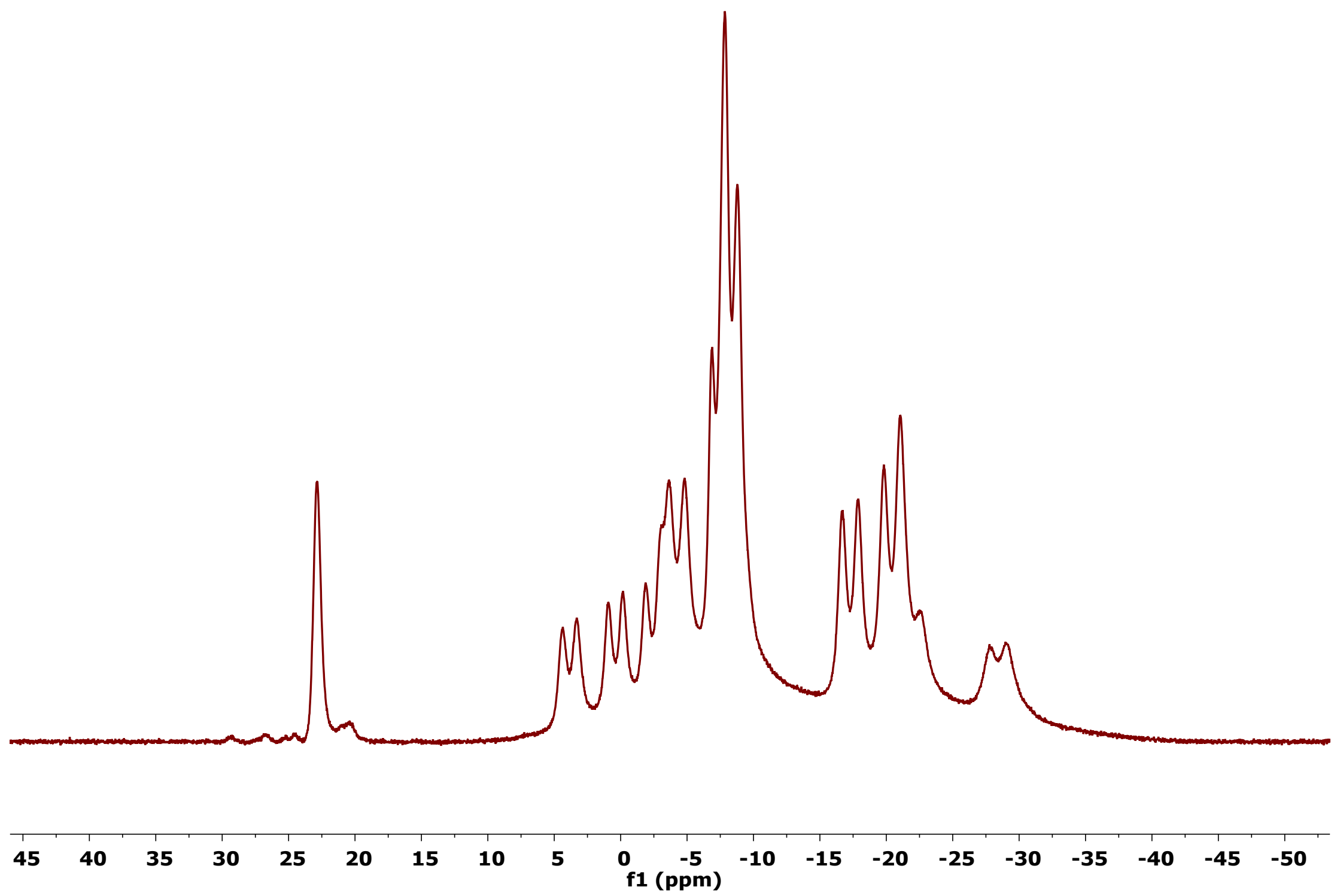
45 40 35 30 25 20 15 10 5 0 -5 -10 -15 -20 -25 -30 -35 -40 -45 -50
f1 (ppm)

e)

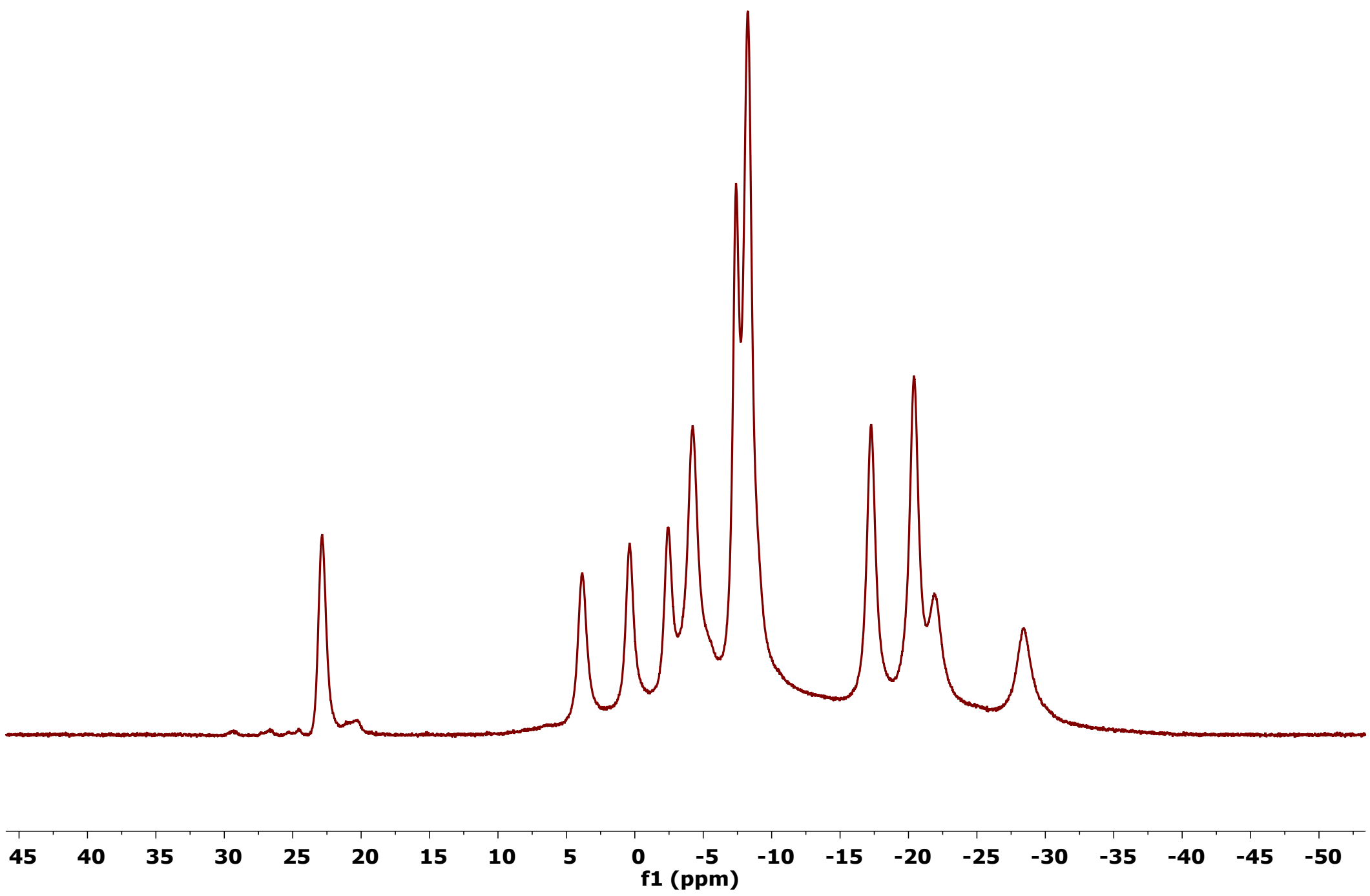
45 40 35 30 25 20 15 10 5 0 -5 -10 -15 -20 -25 -30 -35 -40 -45 -50
f1 (ppm)

f)

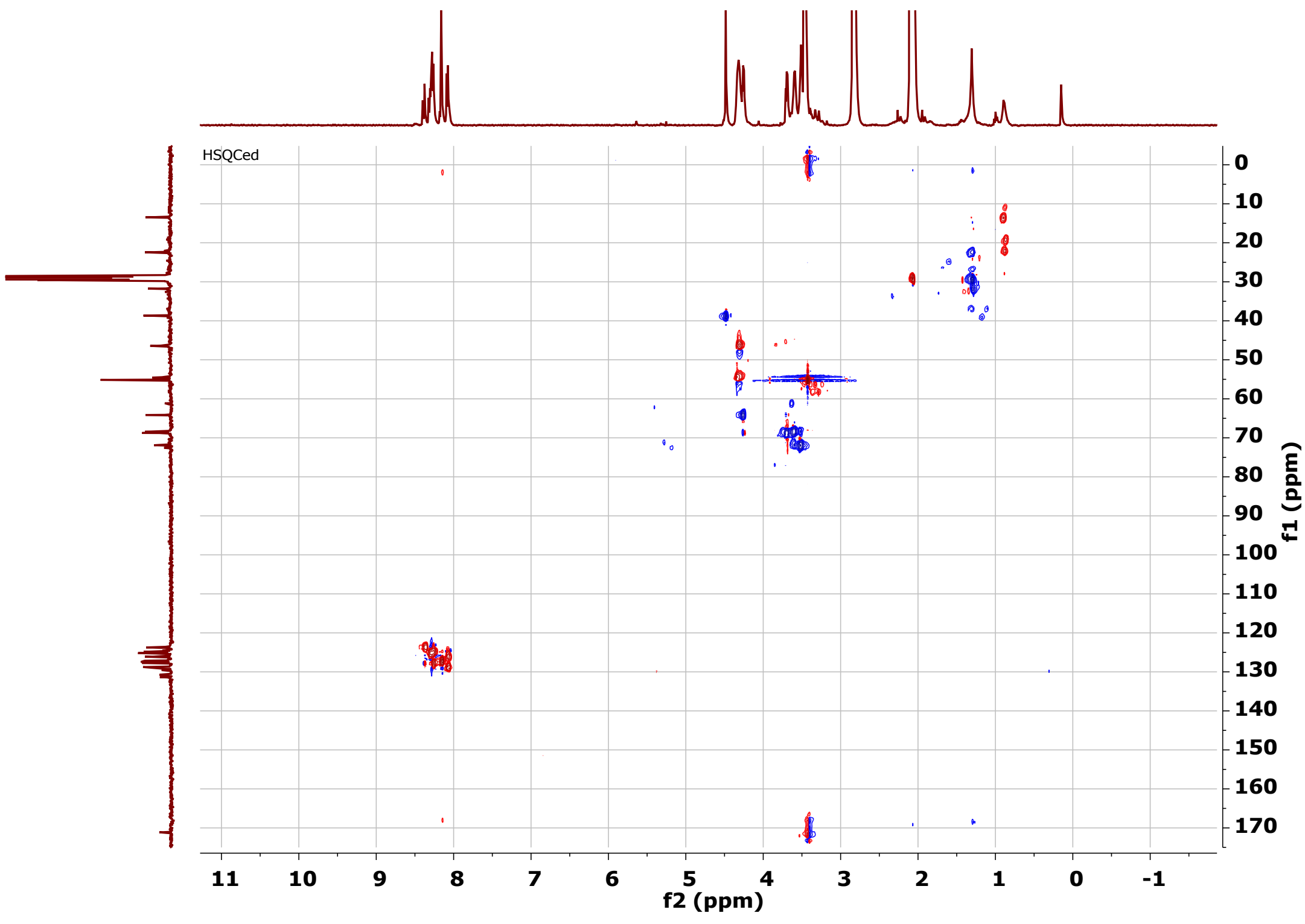
HSQCed
0 10 20 30 40 50 60 70 80 90 100 110 120 130 140 150 160 170
f1 (ppm)
11 10 9 8 7 6 5 4 3 2 1 0 -1
f2 (ppm)

Figure S4. UV-*vis* spectra of a) Na[**3**] (0.010 mM); b) [N(CH_3)$_4$][**3**] (0.015 mM) in CH_3CN and c) spectral superposition of [3,3'-Co(8-$C_4H_8O_2$-1,2-$C_2B_9H_{10}$)(1',2'-$C_2B_9H_{11}$)], **2** (green) and [Na][**3**] in CH_2Cl_2 (0.080 mM); (blue).

.

a)

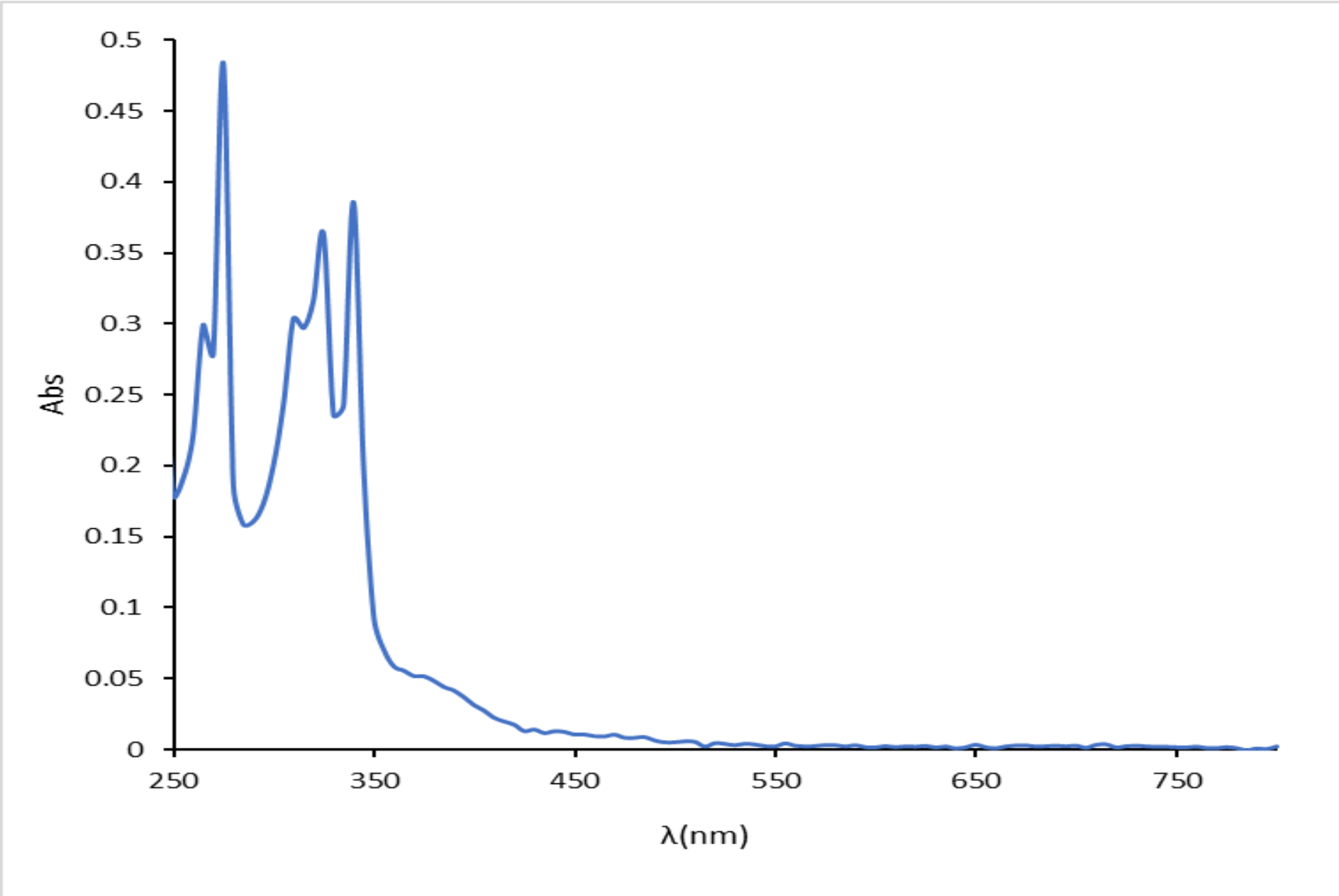

b)

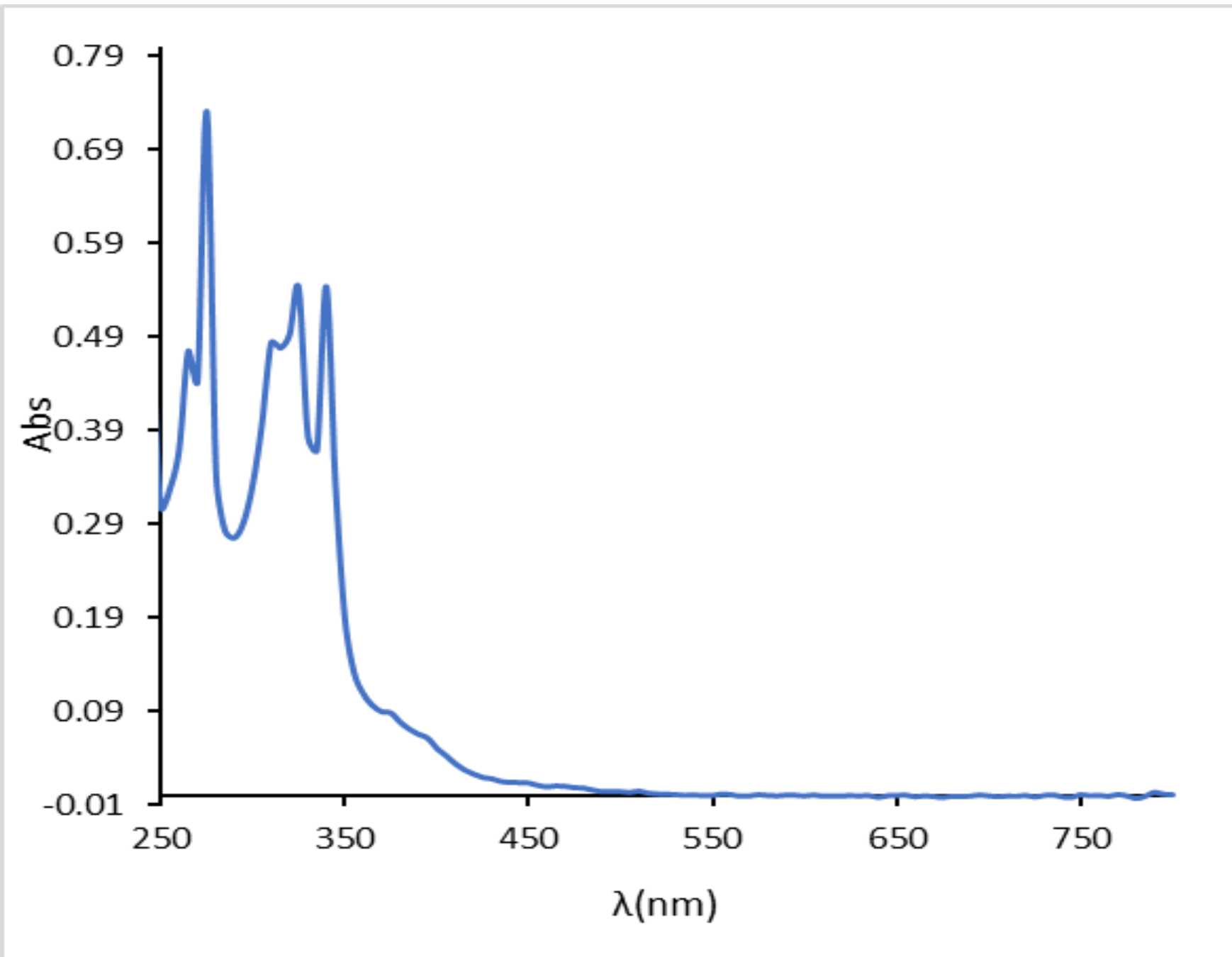

c)

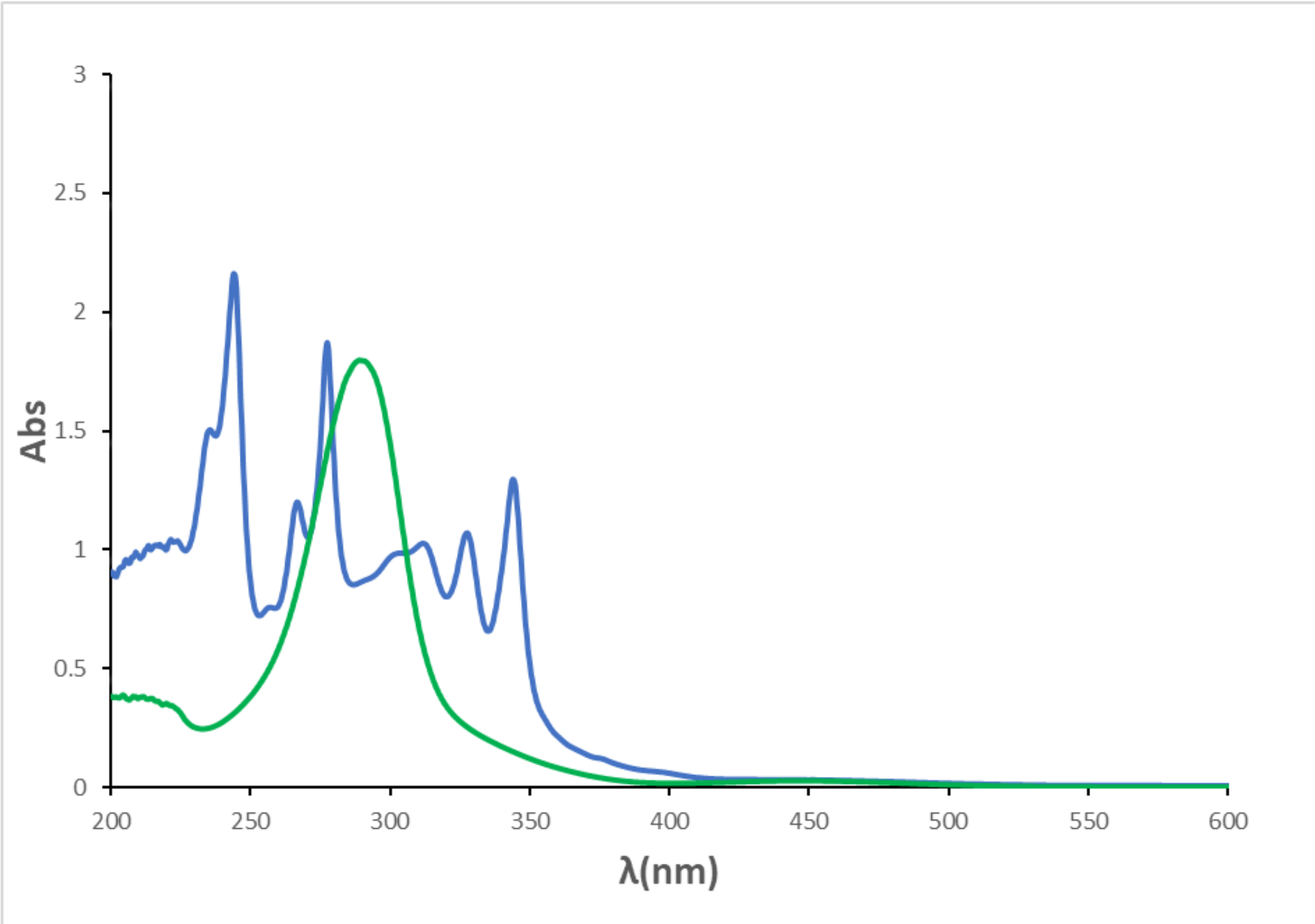

Figure S5. ESI-MS of Na[**3**] in a) negative mode and b) positive mode

a)

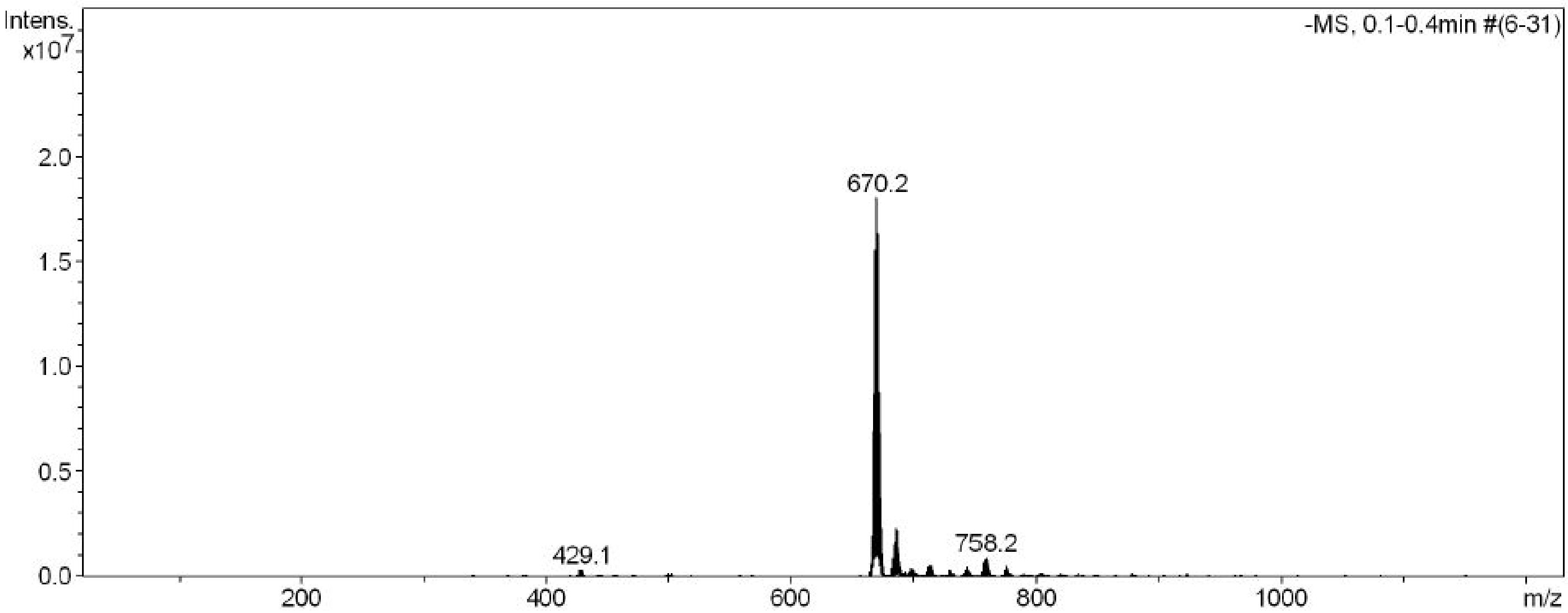

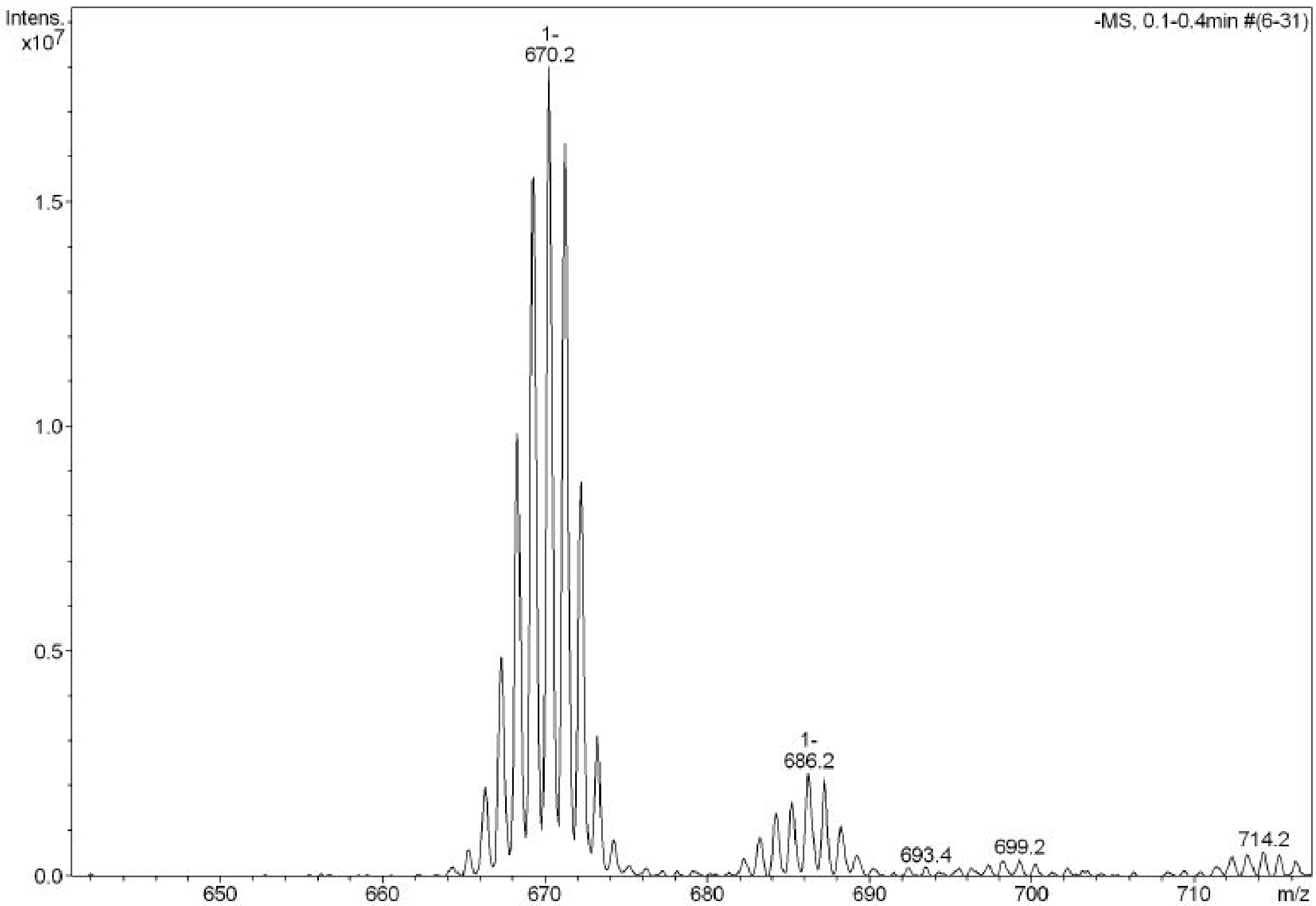

b)

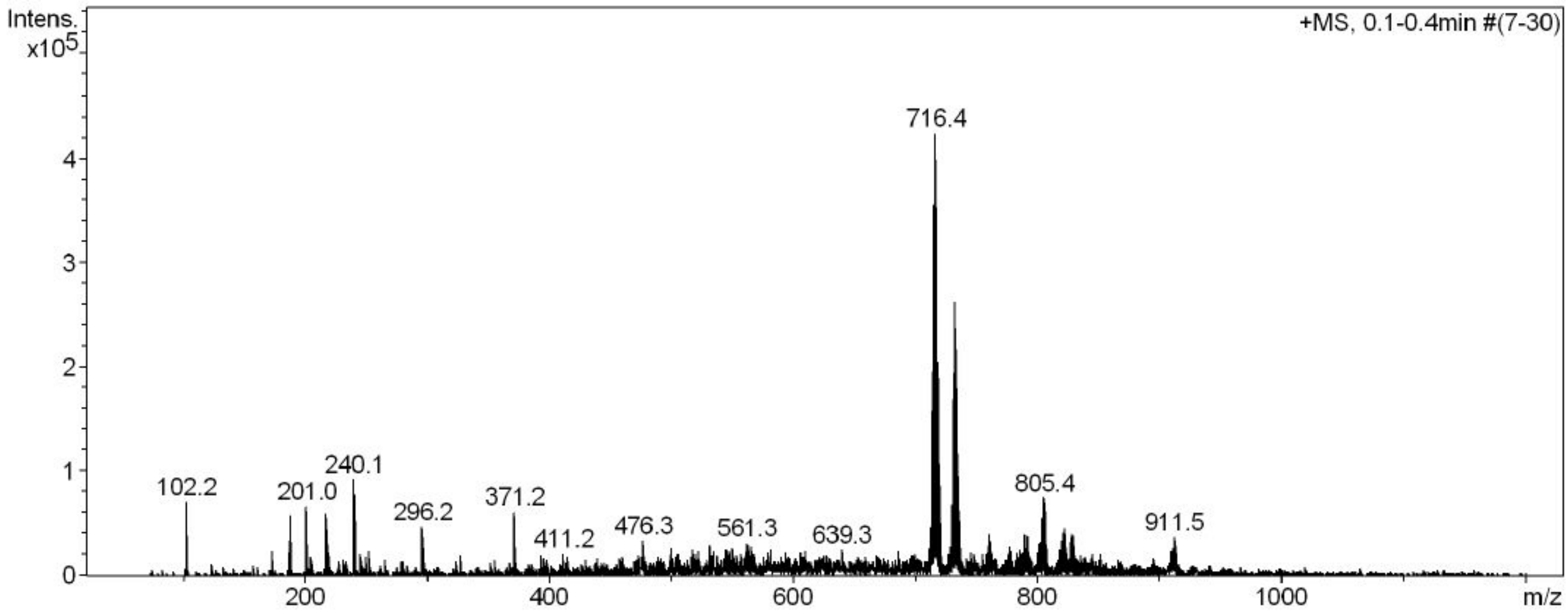

Intens.
x10^5
+MS, 0.1-0.4min #(7-30)
716.4
805.4
102.2
201.0
240.1
296.2
371.2
411.2
476.3
561.3
639.3
911.5
m/z

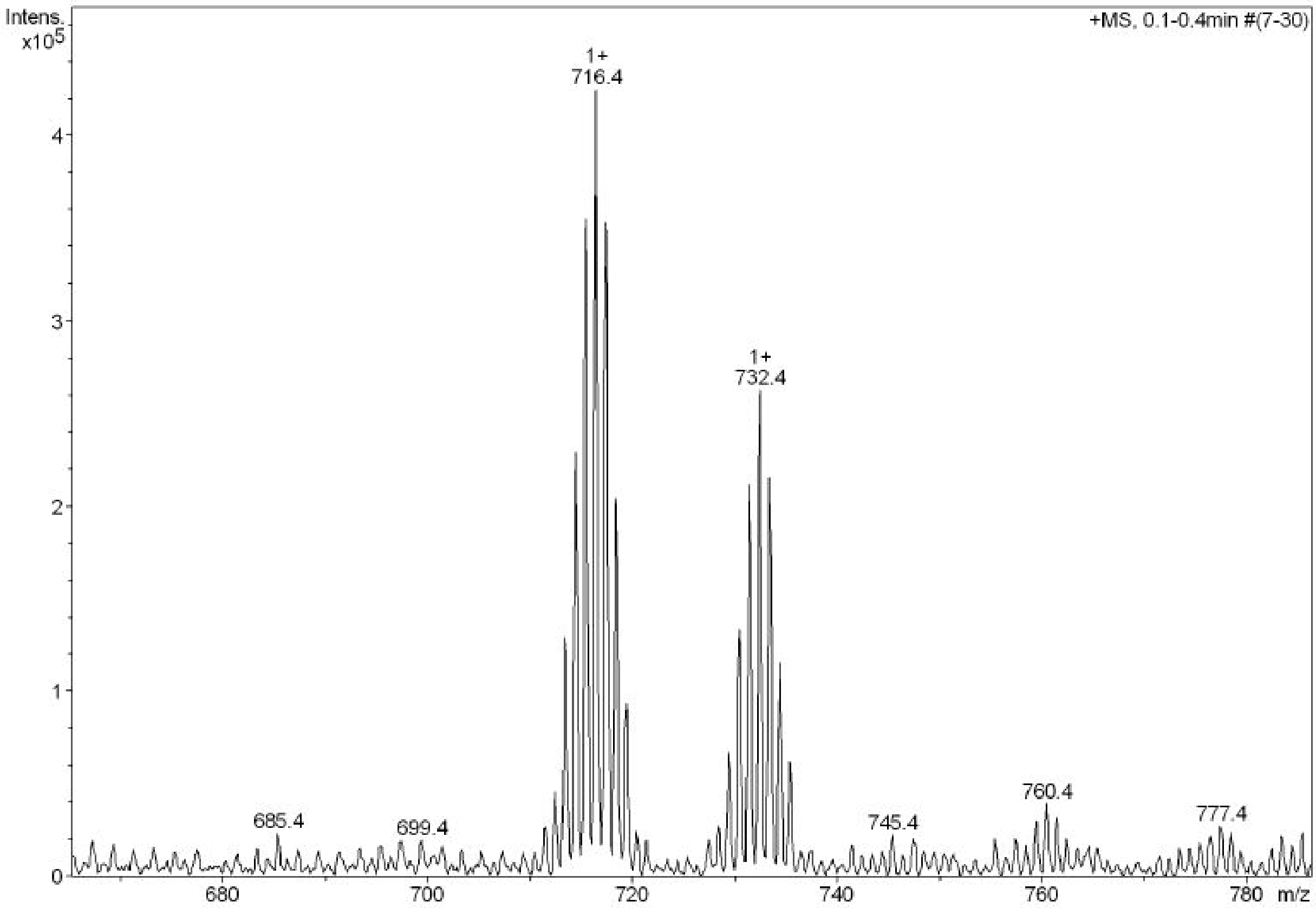

Intens.
x10^5
+MS, 0.1-0.4min #(7-30)
1+
716.4
1+
732.4
685.4
699.4
745.4
760.4
777.4
m/z

Figure S6. ESI-MS of $[N(CH_3)_4][\mathbf{3}]$ in a) negative mode and b) positive mode.

a)

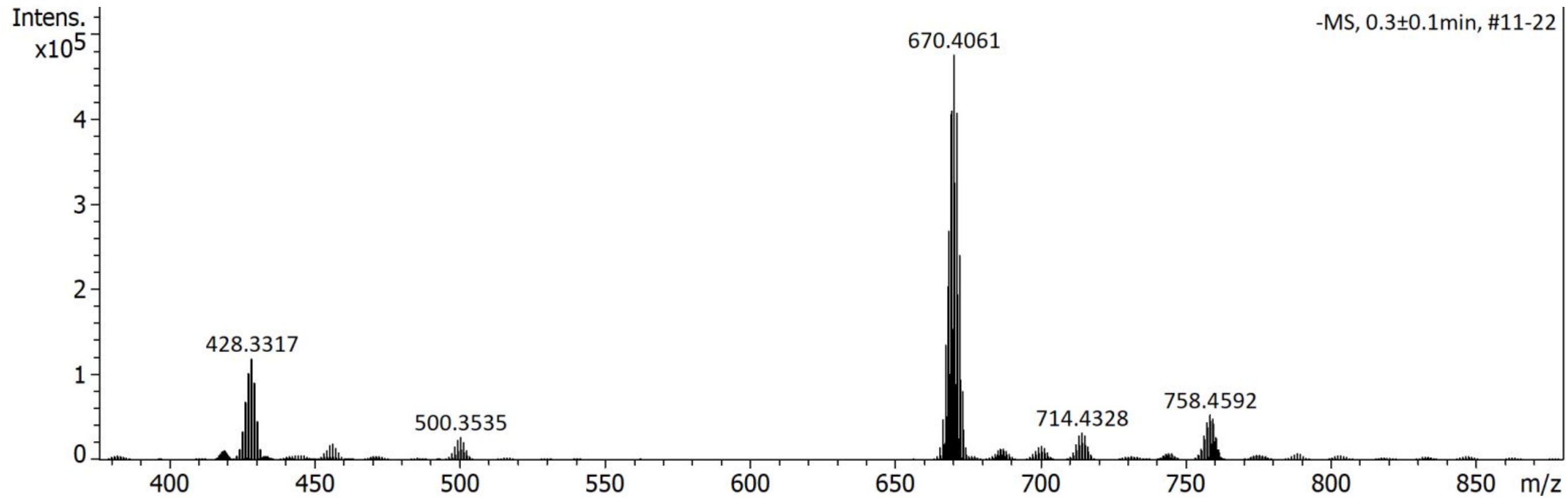

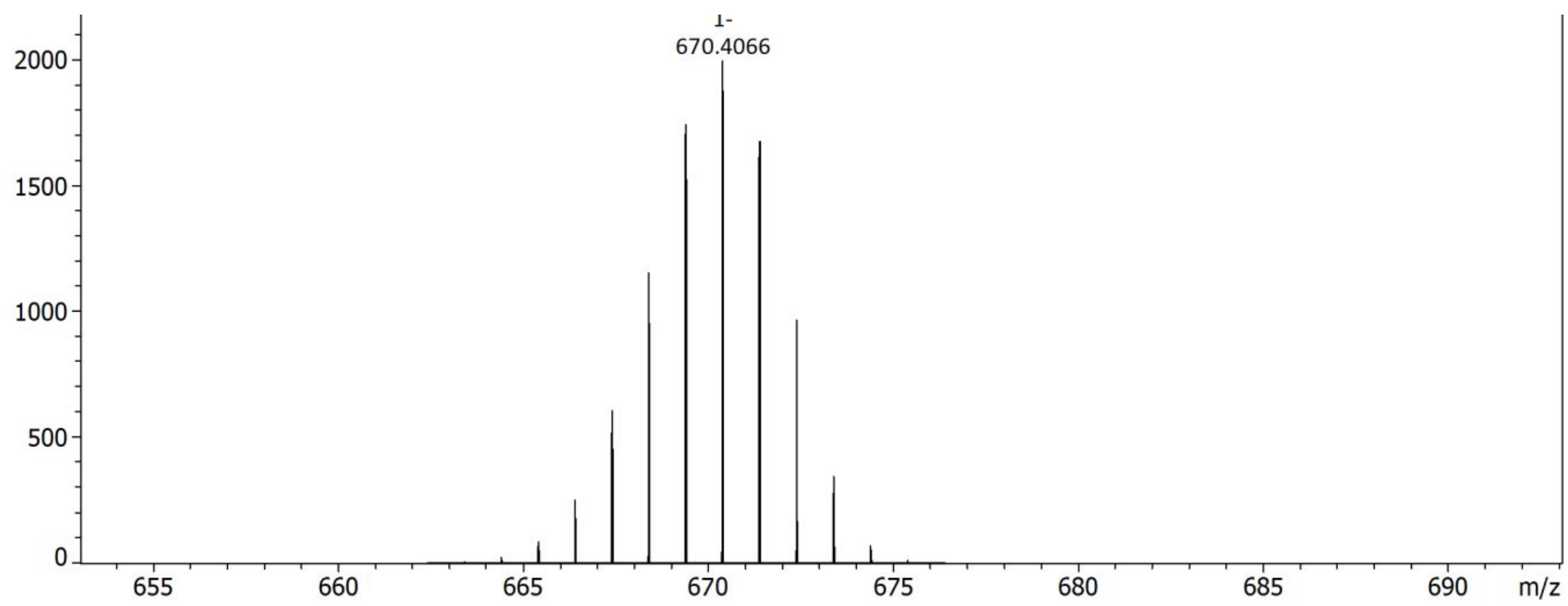

b)

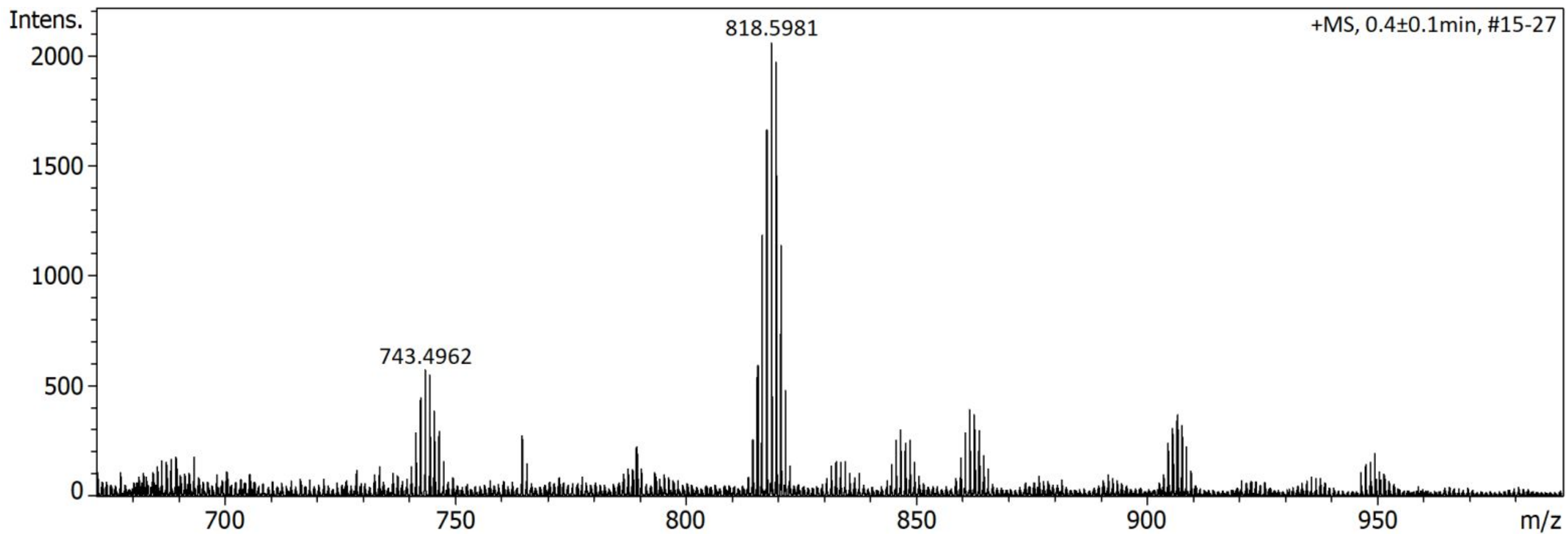
Intens.
818.5981
+MS, 0.4±0.1min, #15-27
743.4962
2000
1500
1000
500
0
700
750
800
850
900
950
m/z

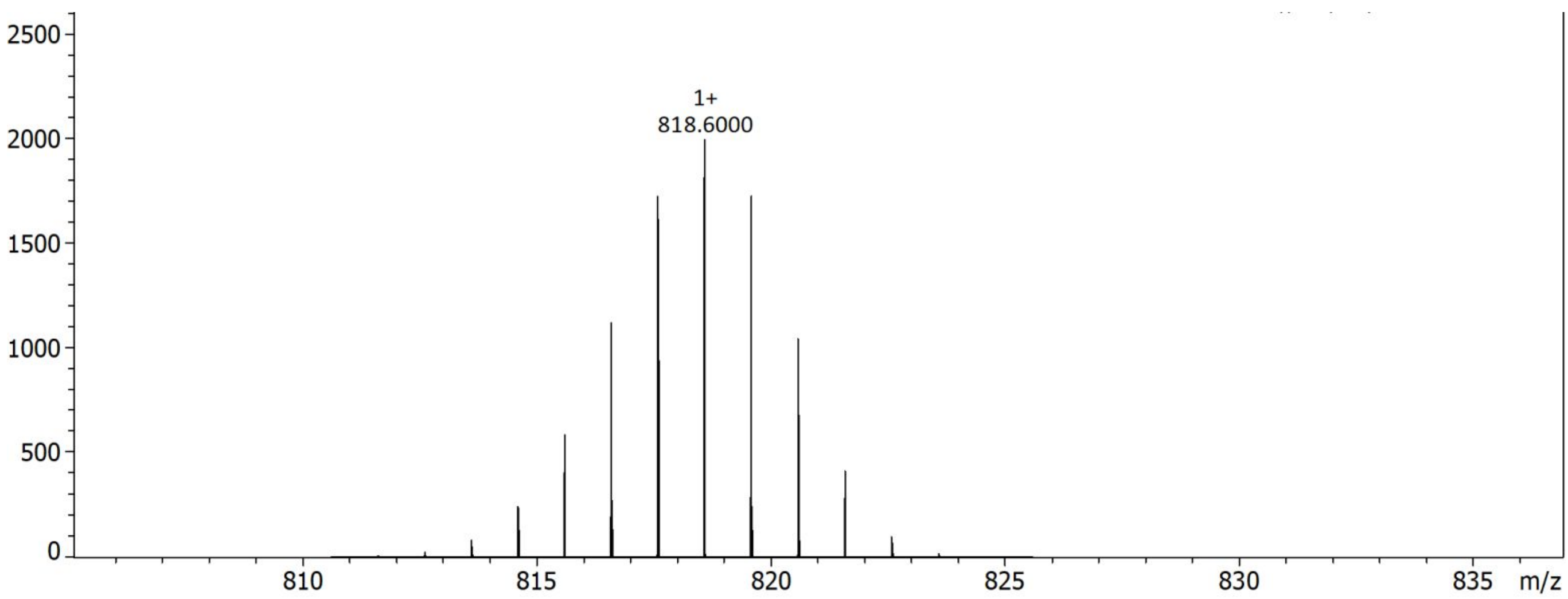
1+
818.6000
2500
2000
1500
1000
500
0
810
815
820
825
830
835
m/z

Figure S7. TGA of Na[**3**].

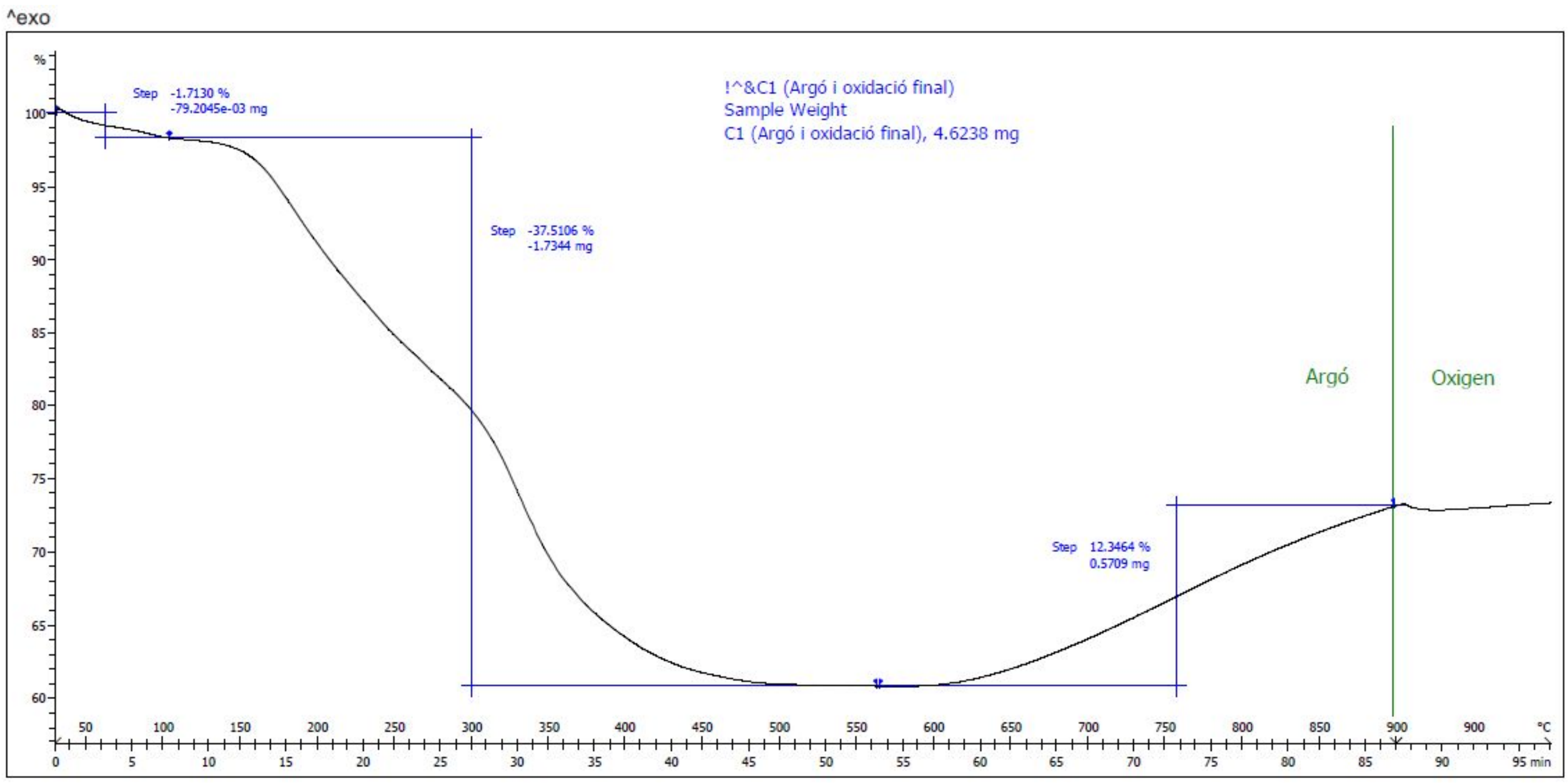

Figure S8. CVs for a) pyreneacetic acid and b) compound **2** in CH_3CN + 0.1 M [n-Bu_4N][PF_6] ($TBAPF_6$) *vs* SCE, showing the anodic and cathodic regions.

a)

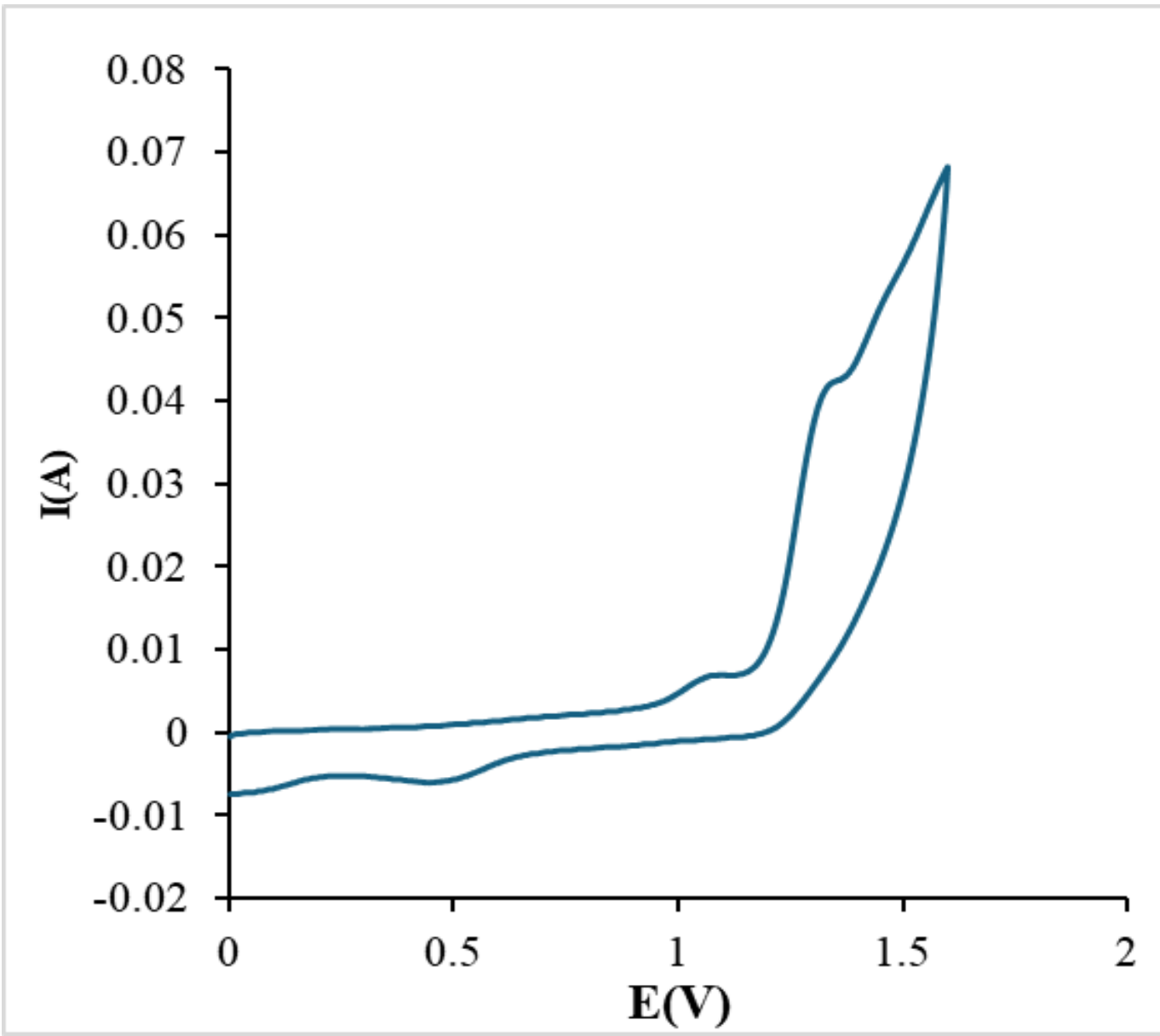

b)

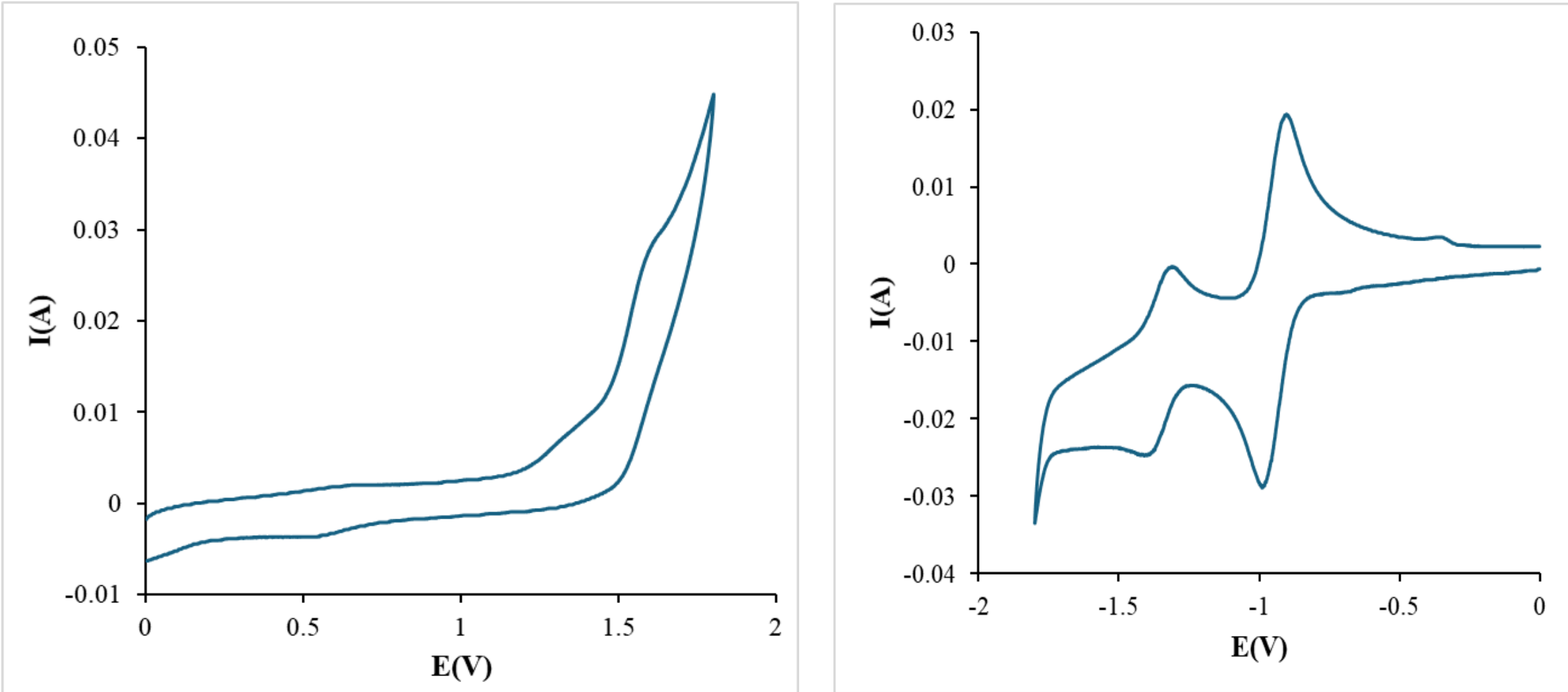

Figure S9. CVs for a $[N(CH_3)_4]$[**3**] compound in CH_3CN + 0.1 M [n-Bu_4N][PF_6] ($TBAPF_6$) *vs* SCE, showing the anodic and cathodic regions.

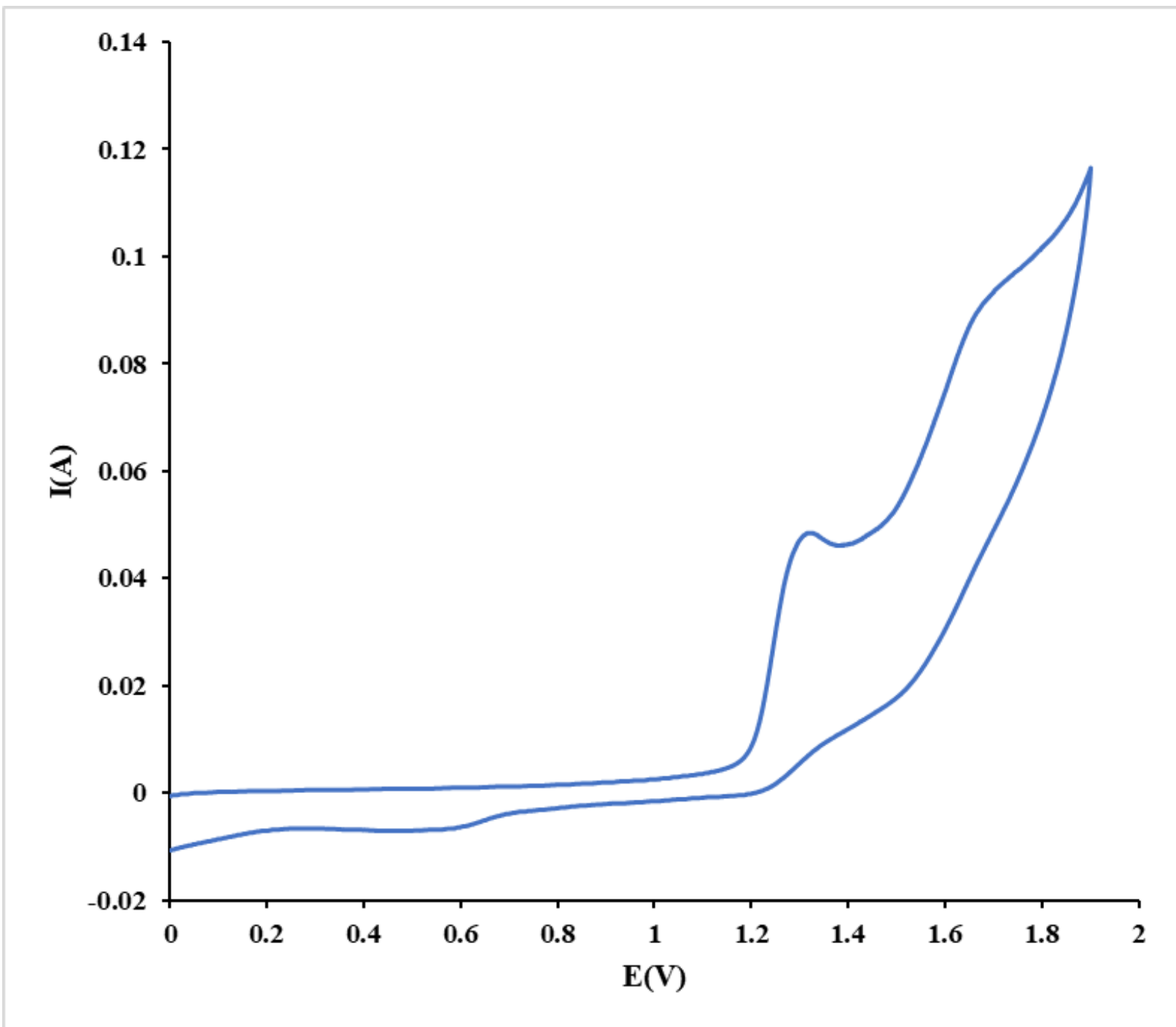

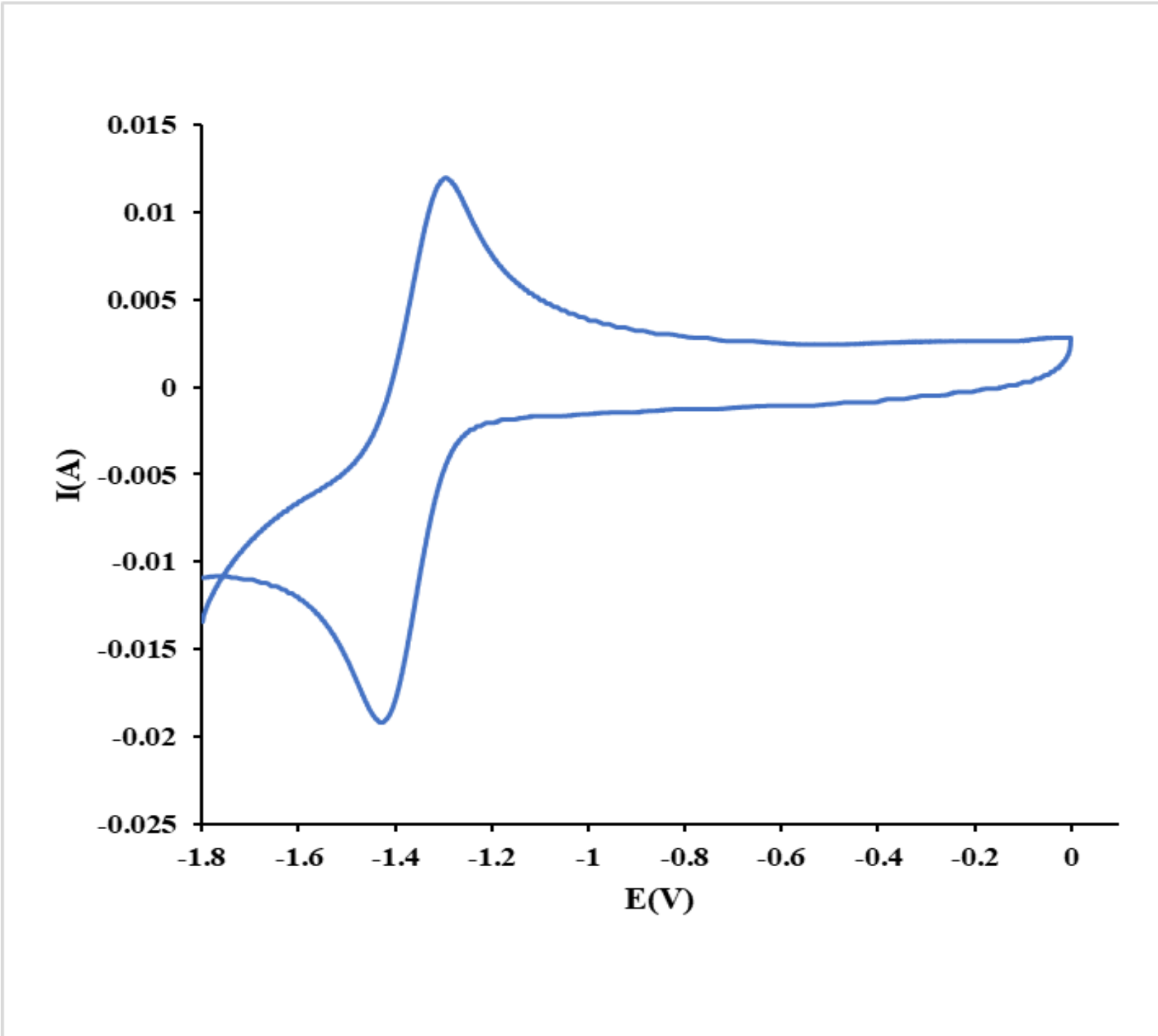

Figure S10. DPVs for a) Na[**3**] and b) [N(CH_3)$_4$][**3**] compounds in CH_3CN + 0.1 M [*n*-Bu_4N][PF_6] ($TBAPF_6$) *vs* SCE, showing the anodic and cathodic regions.

a)

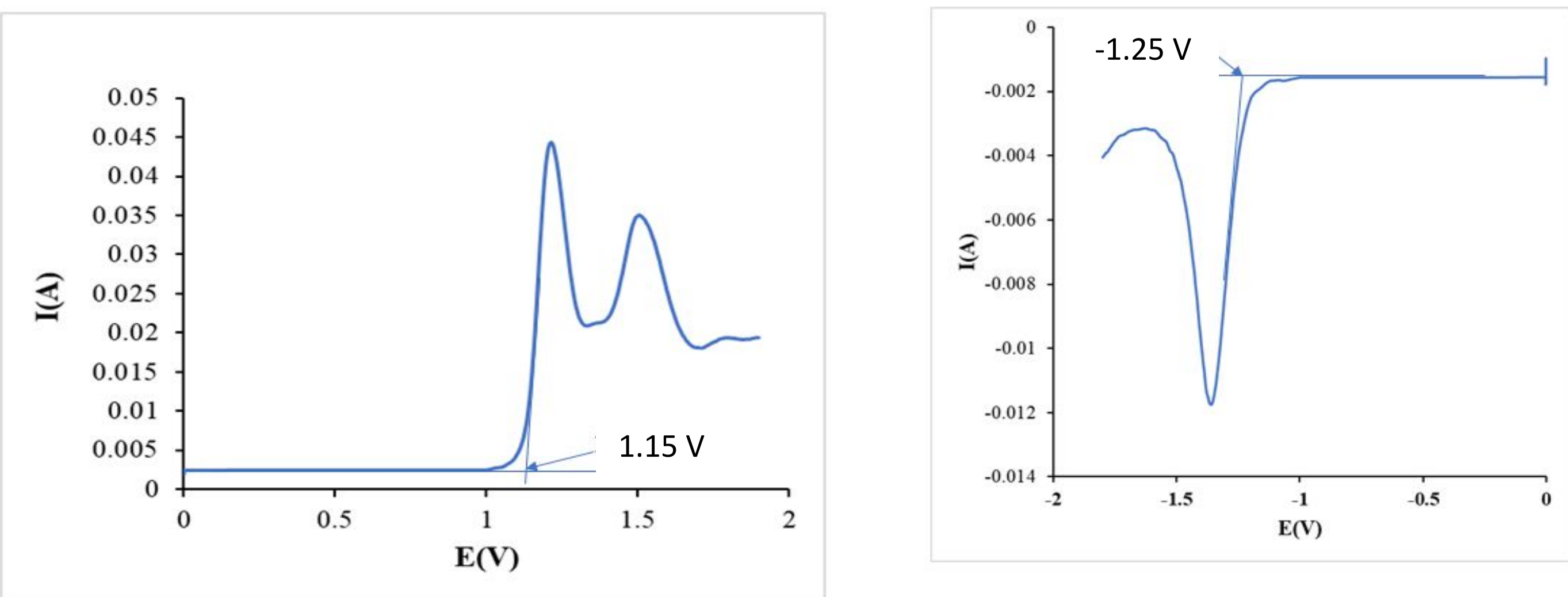

b)

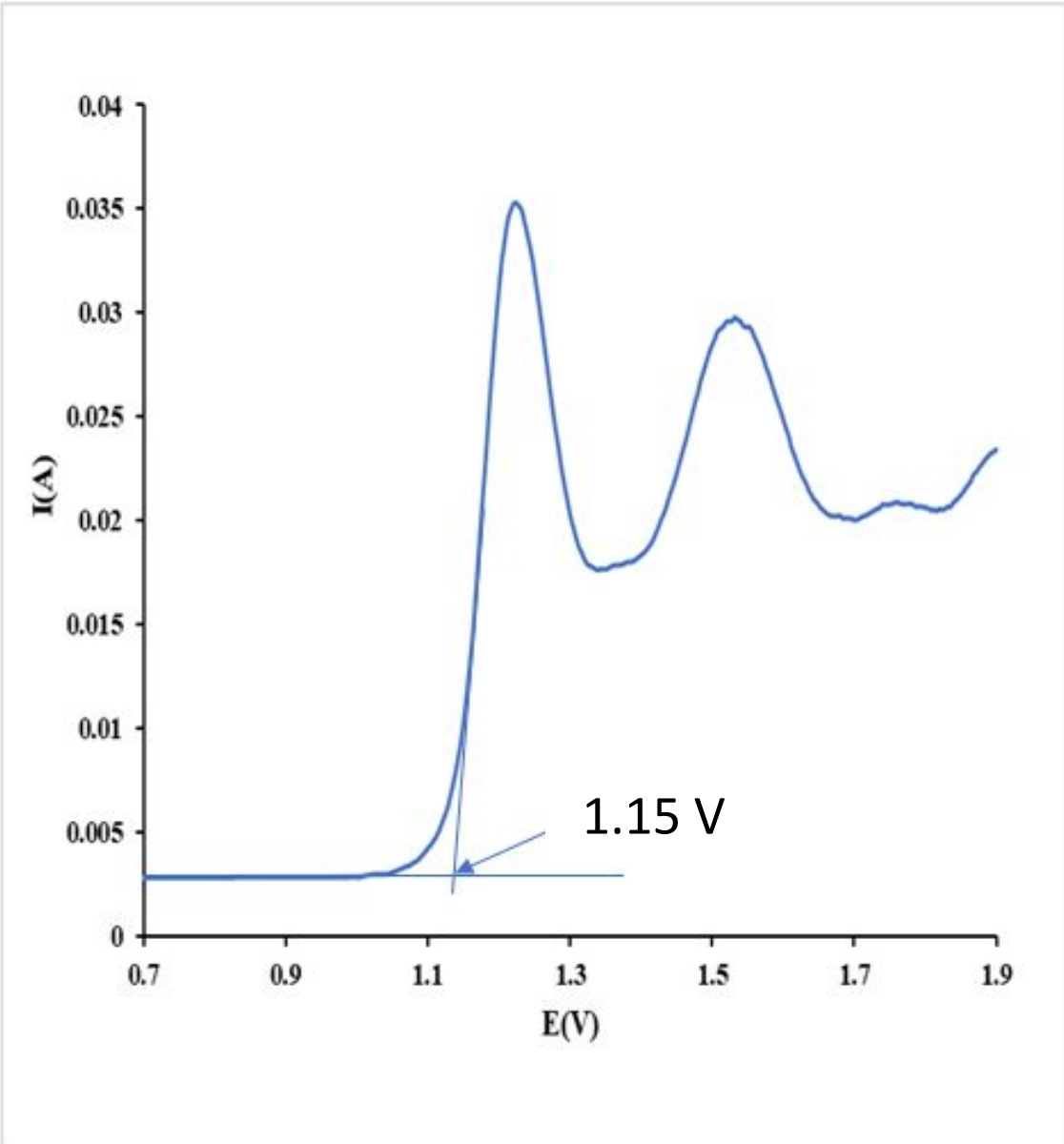

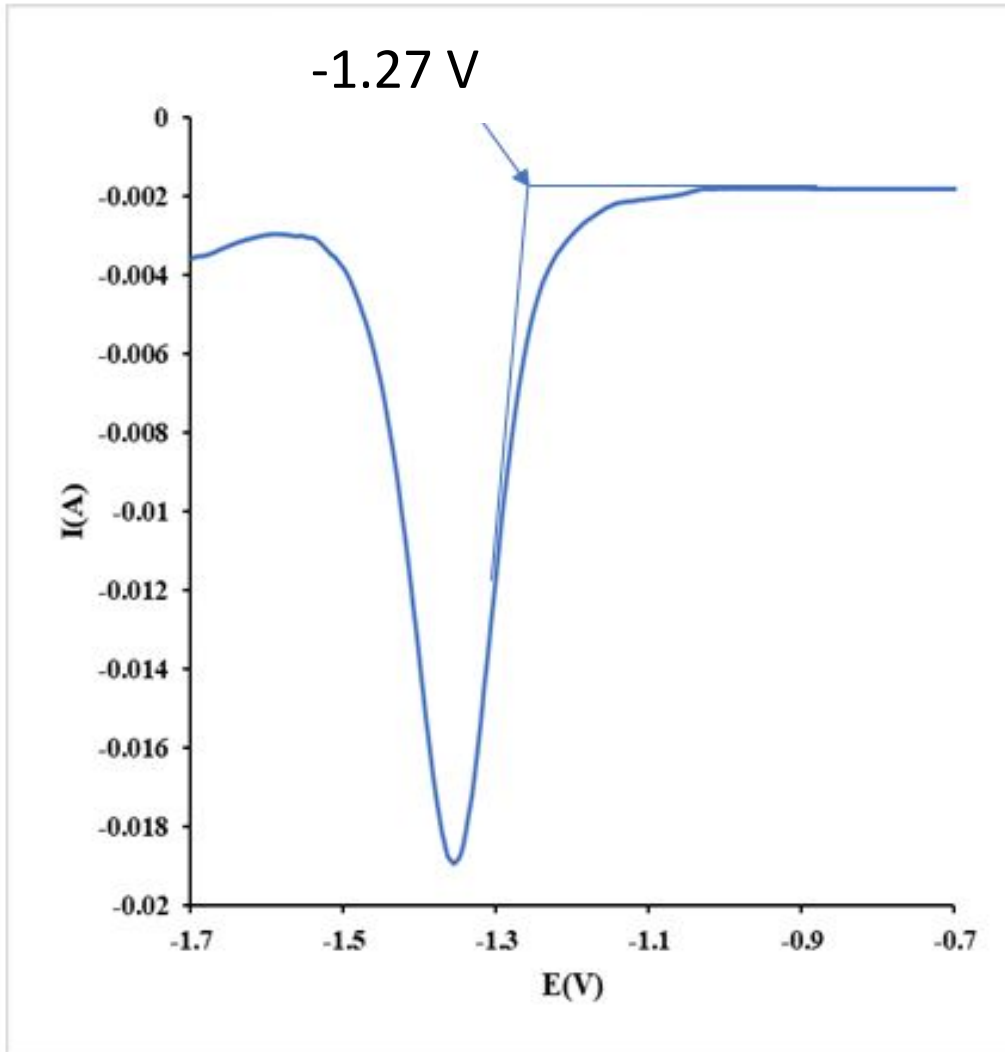

Figure S11. Size distribution graphs for a) Na[**3**] and b) $[N(CH_3)_4]$[**3**] at concentration of 0.020 mM in water:acetone (1:1) and for c) Na[**3**] and d) $[N(CH_3)_4]$[**3**] at concentration of 0.002 mM.

a)

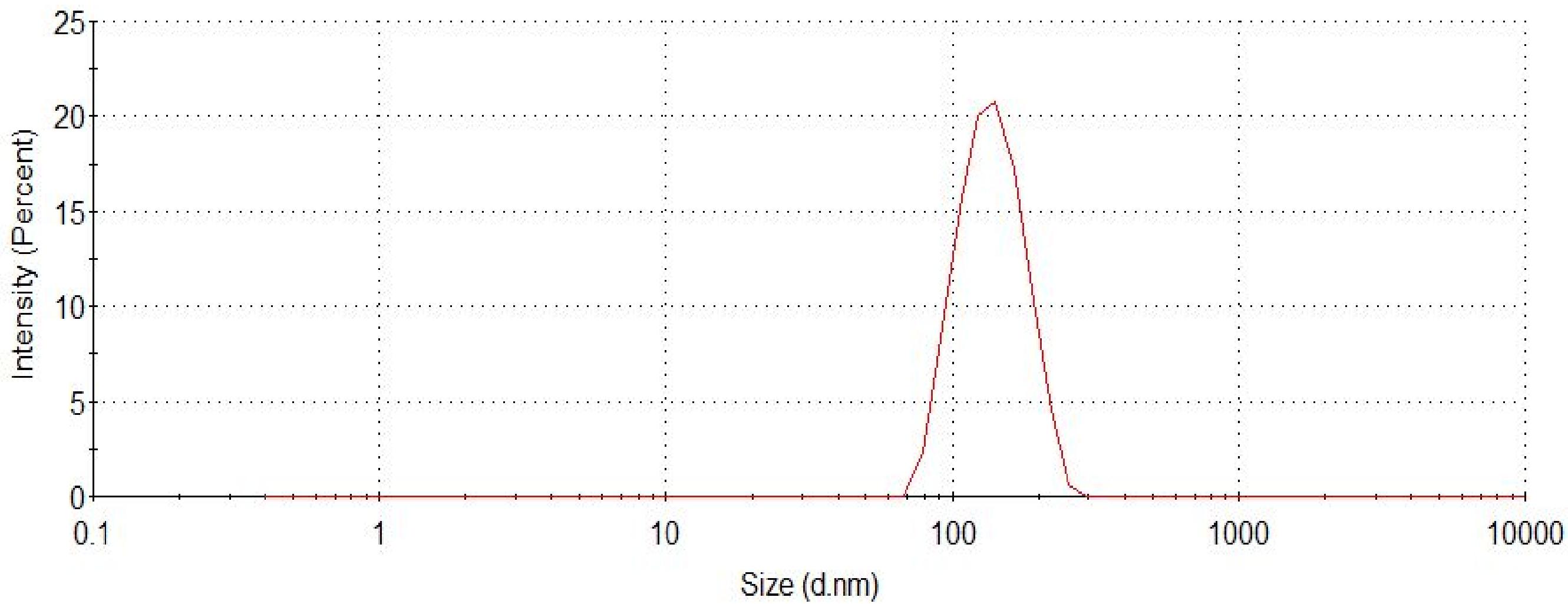

b)

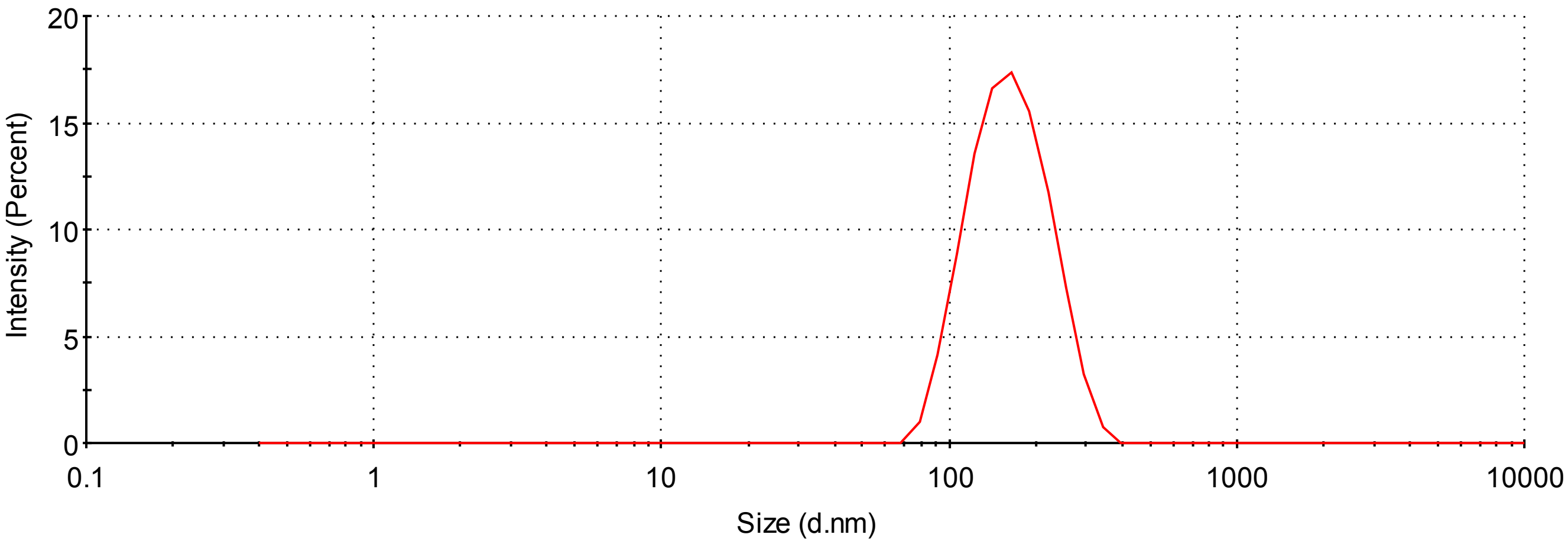

Intensity (Percent)
20
15
10
5
0
0.1
1
10
100
1000
10000
Size (d.nm)

c)

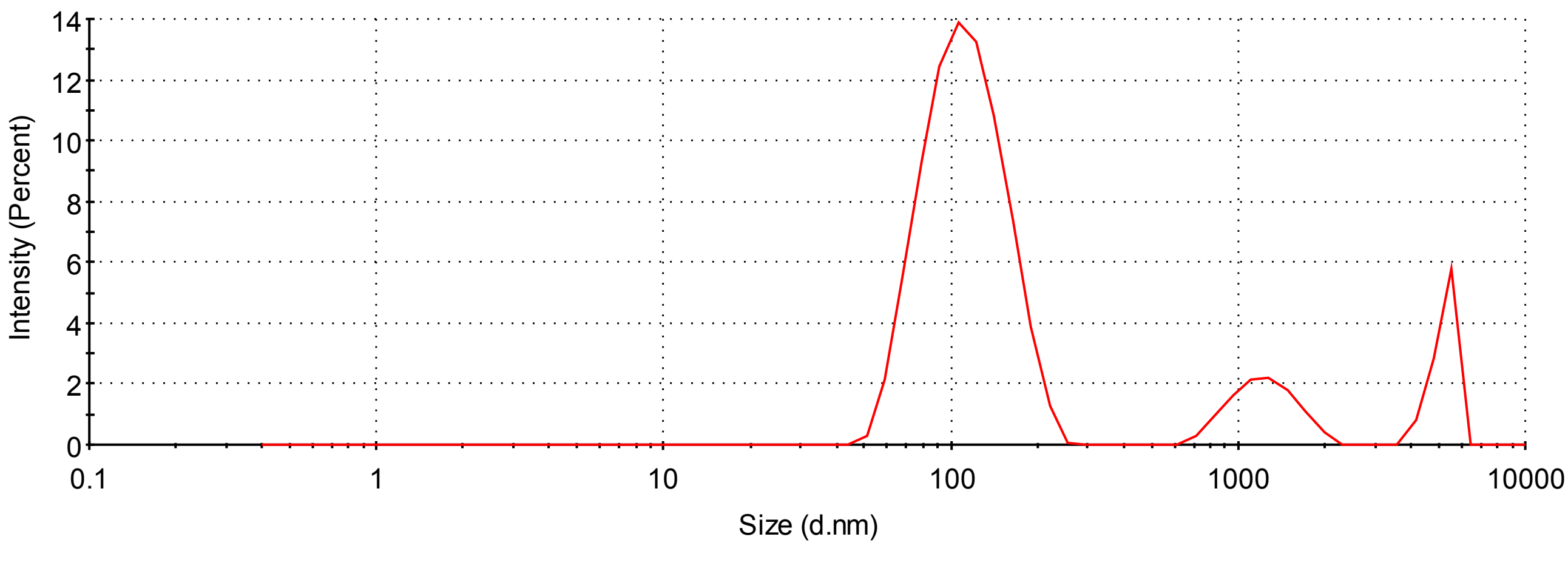

Intensity (Percent)
14
12
10
8
6
4
2
0
0.1
1
10
100
1000
10000
Size (d.nm)

d)

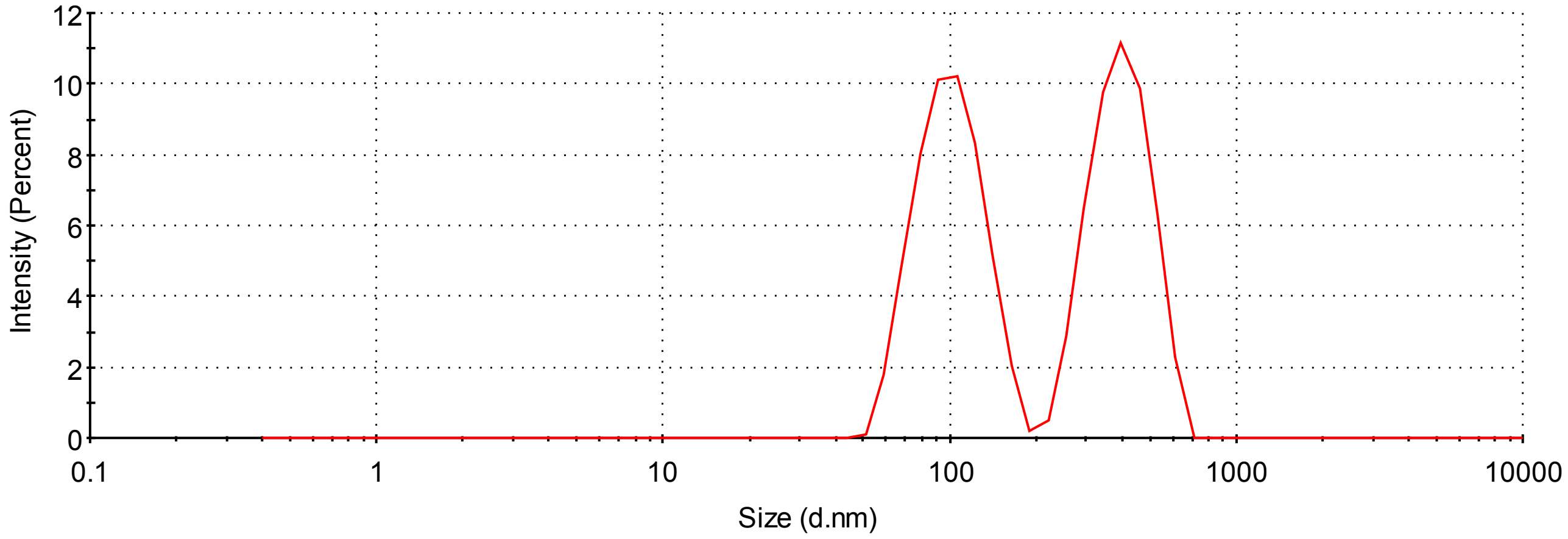

Figure S12. UV-*vis* spectrum of Na[**3**] (0.1mM) in water/acetone (blue line); UV-visible of the initial solution after 24 h in contact with GO (orange line).

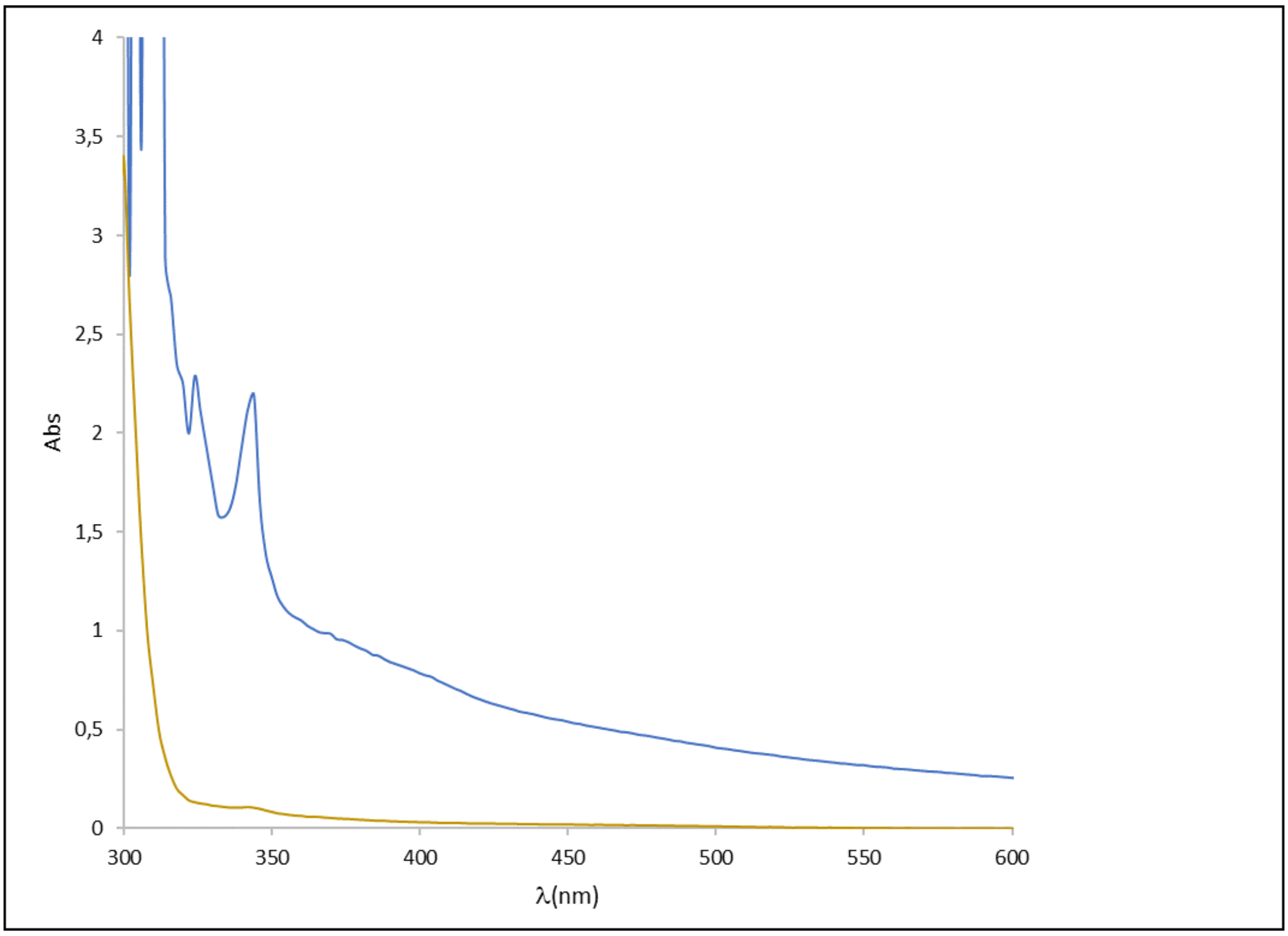

Figure S13. a) TEM images of GO@**3**, (the bars indicate a length of a) 200 nm b)100 nm and c) 20nm).

a)

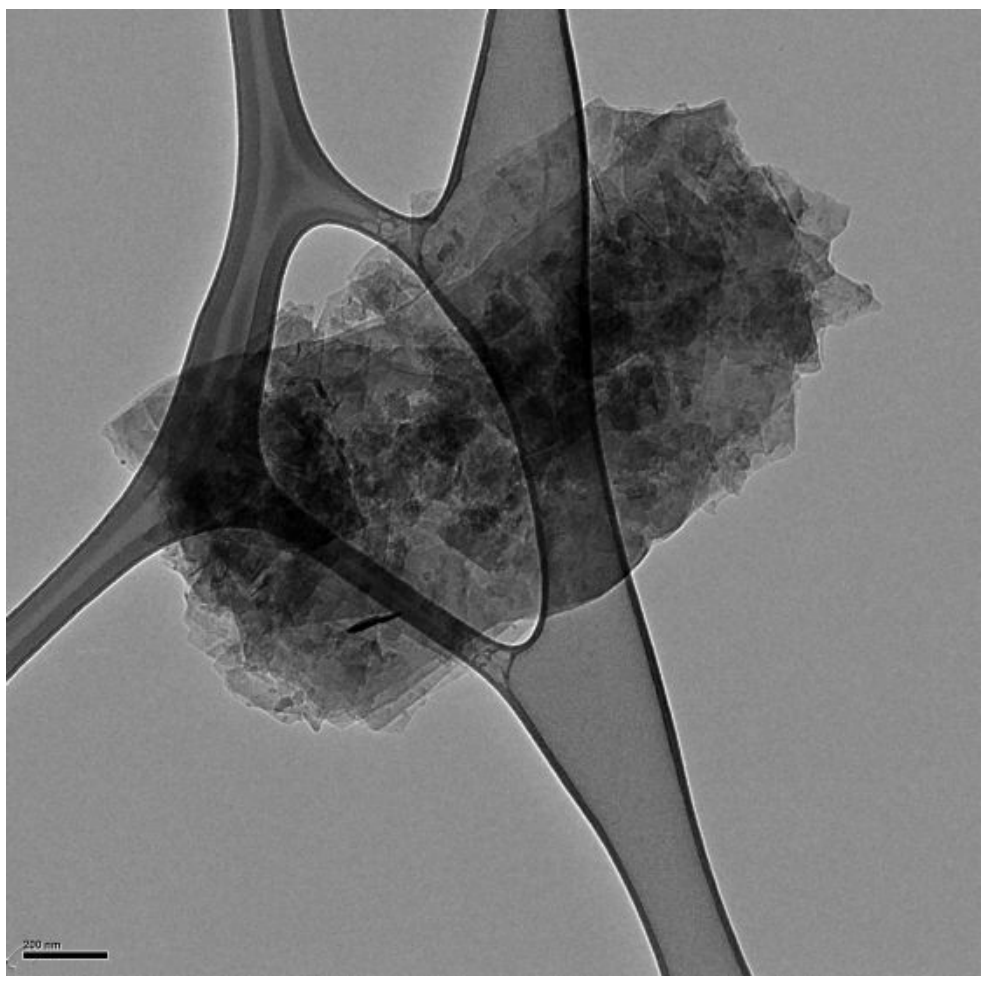

b)

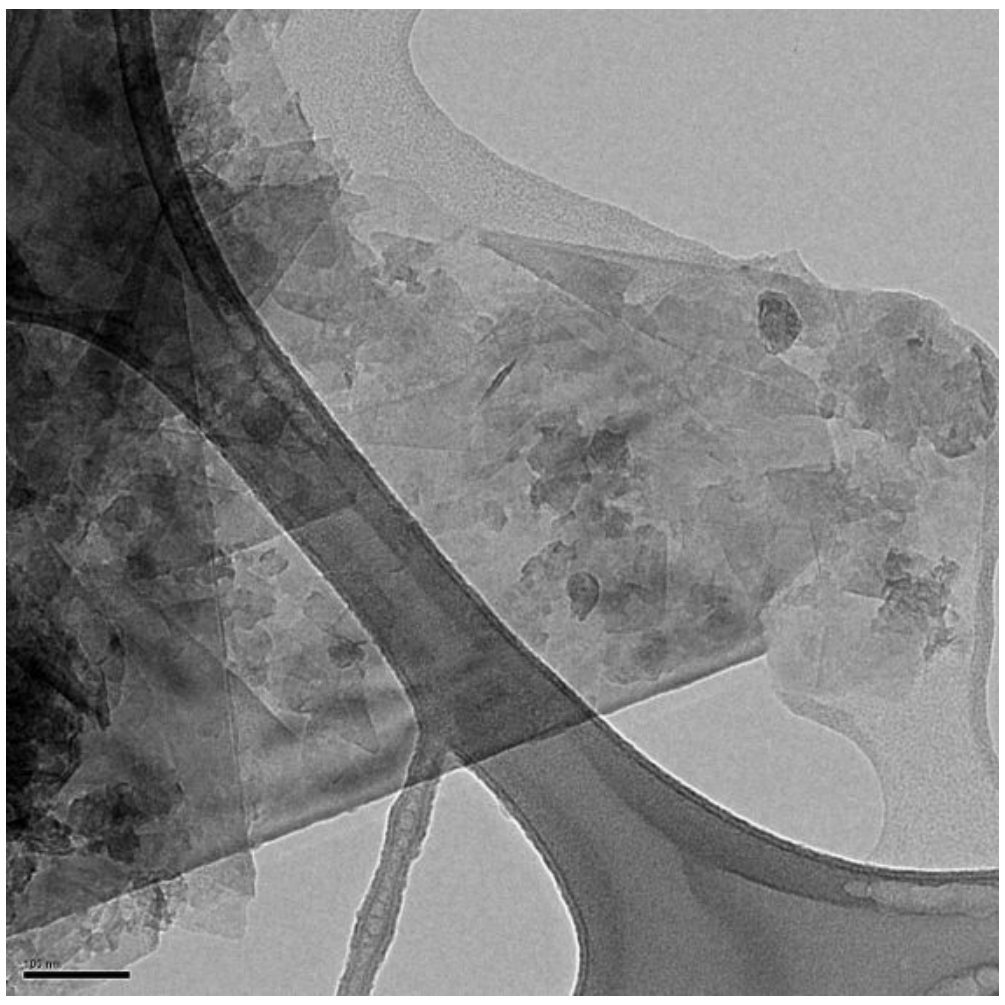

c)

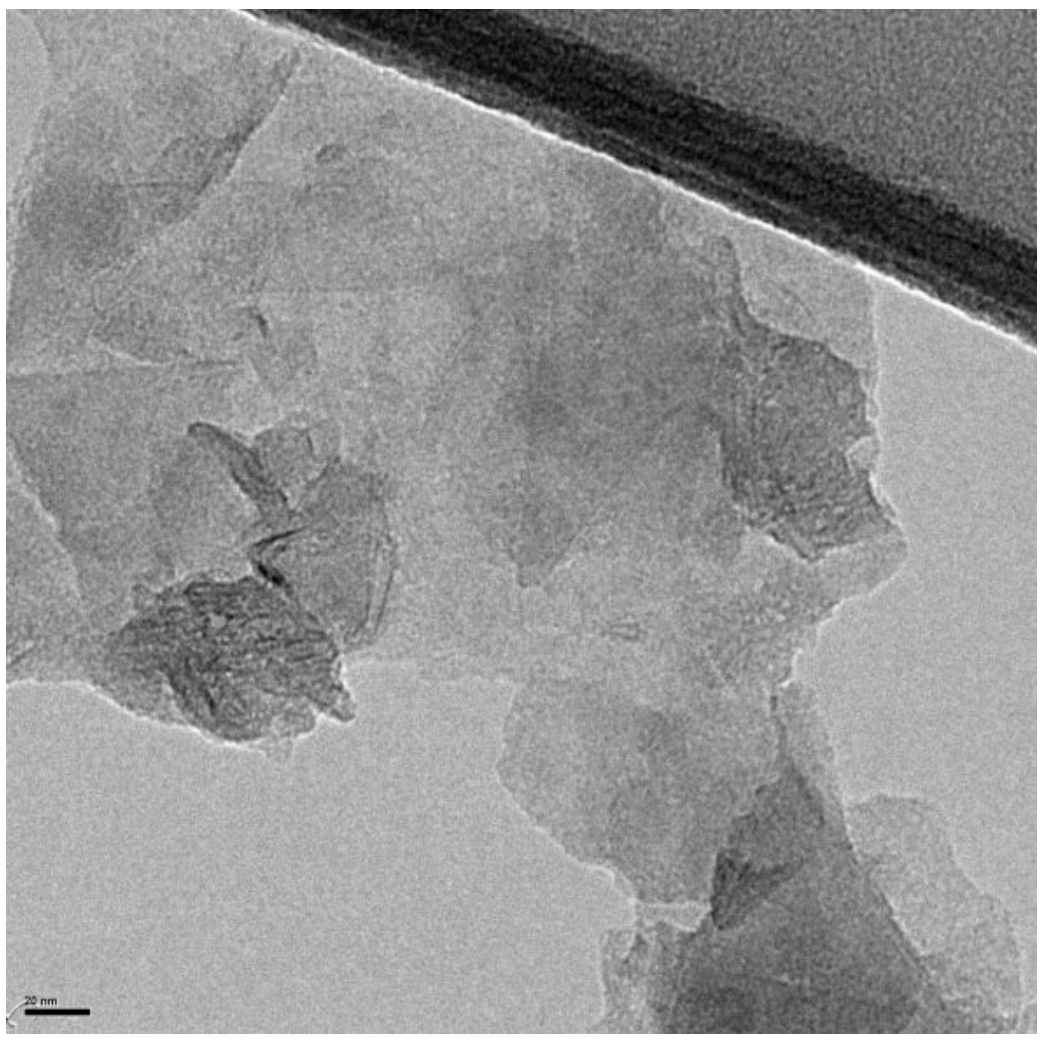

Figure S14. EDX spectra of GO@**3**.

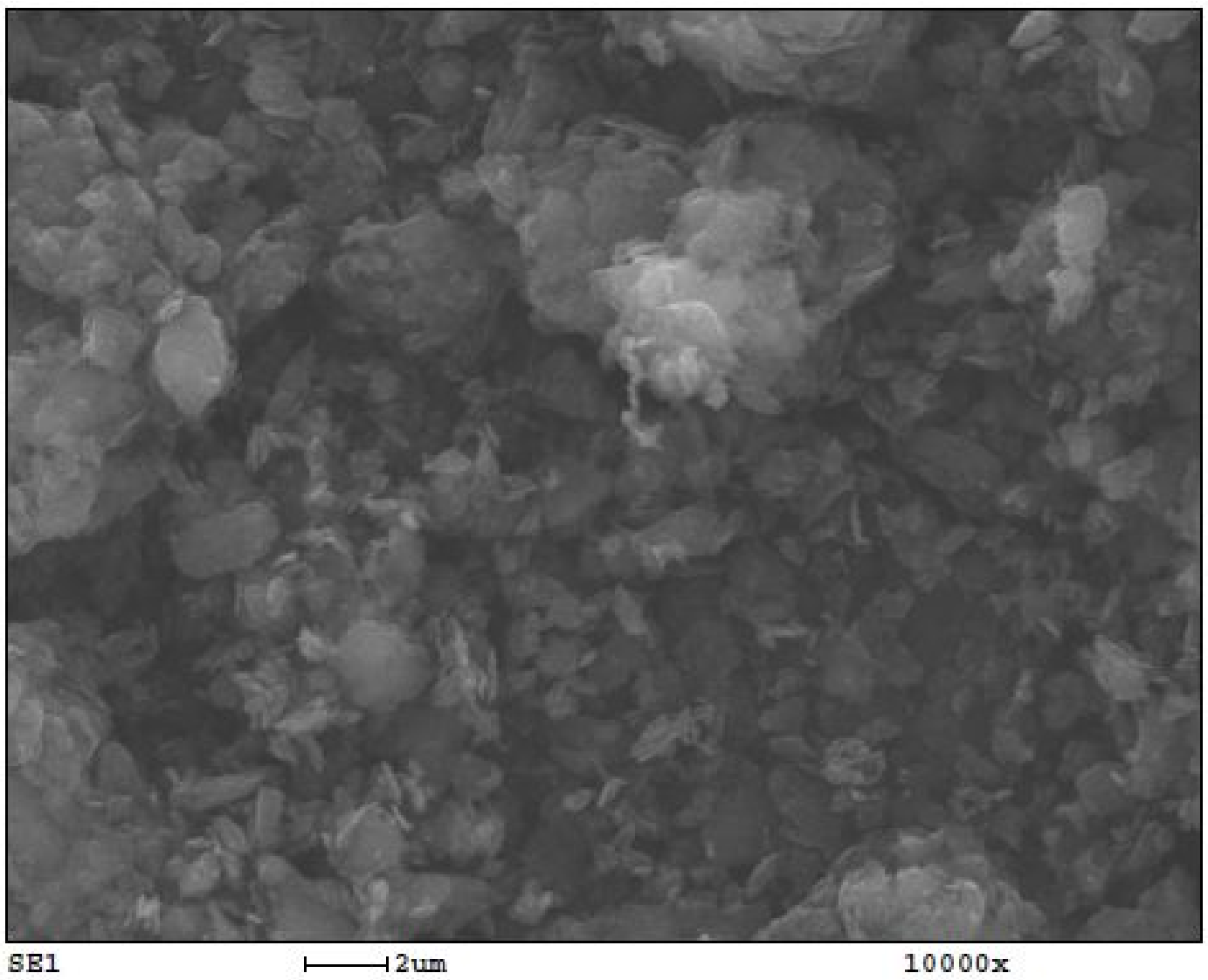

EDS Quantitative Results

Element	Wt%	At%
BK	11.65	12.86
CK	86.36	85.82
OK	1.64	1.22
NaK	0.09	0.05
CoK	0.26	0.05

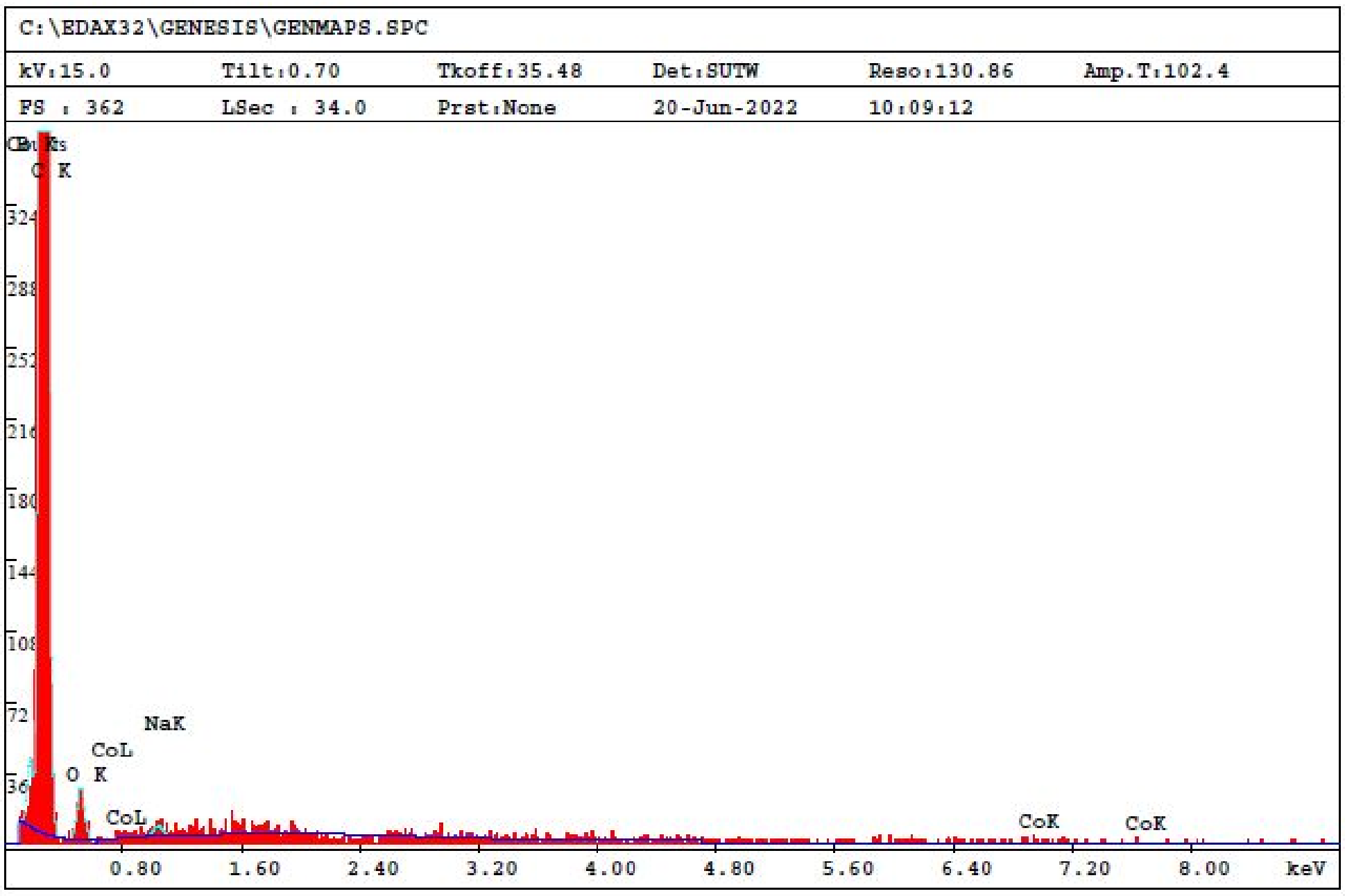

Figure S15. a) XPS spectra of GO@**3**; Magnification of the XPS spectrum of GO@**3** in b) the C1s c) the O1s and d) B 1s region.

a)

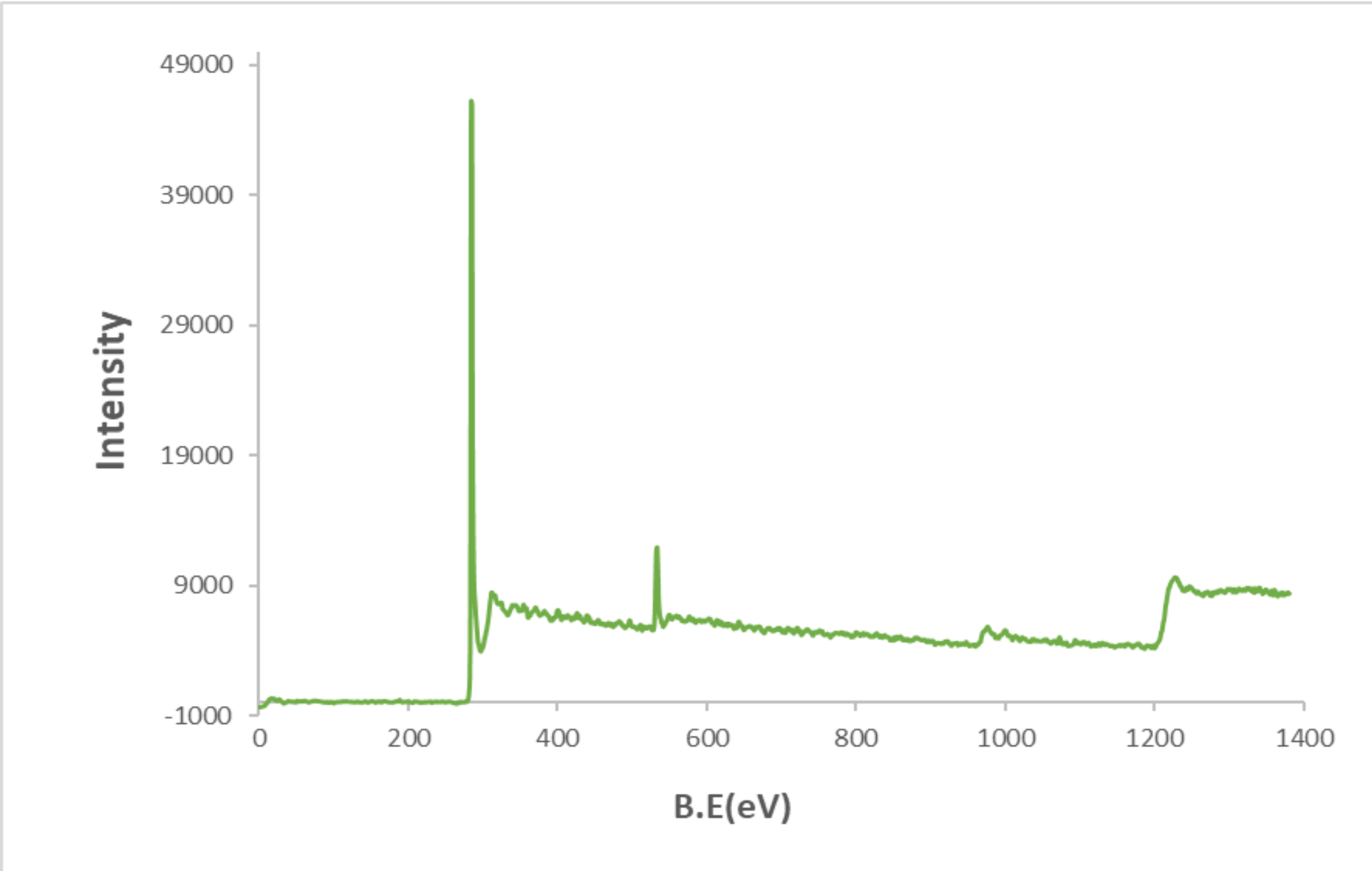

b)

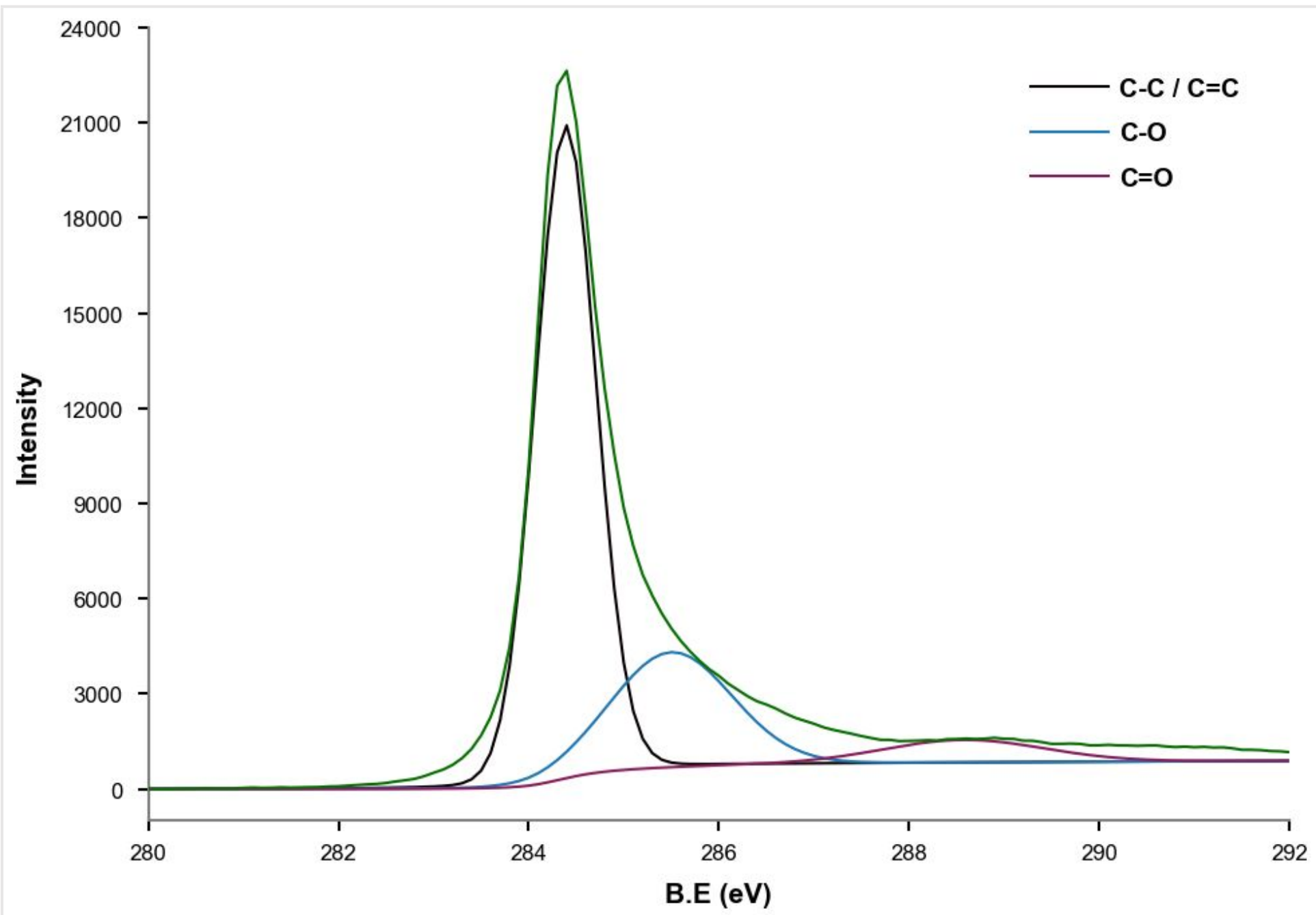

c)

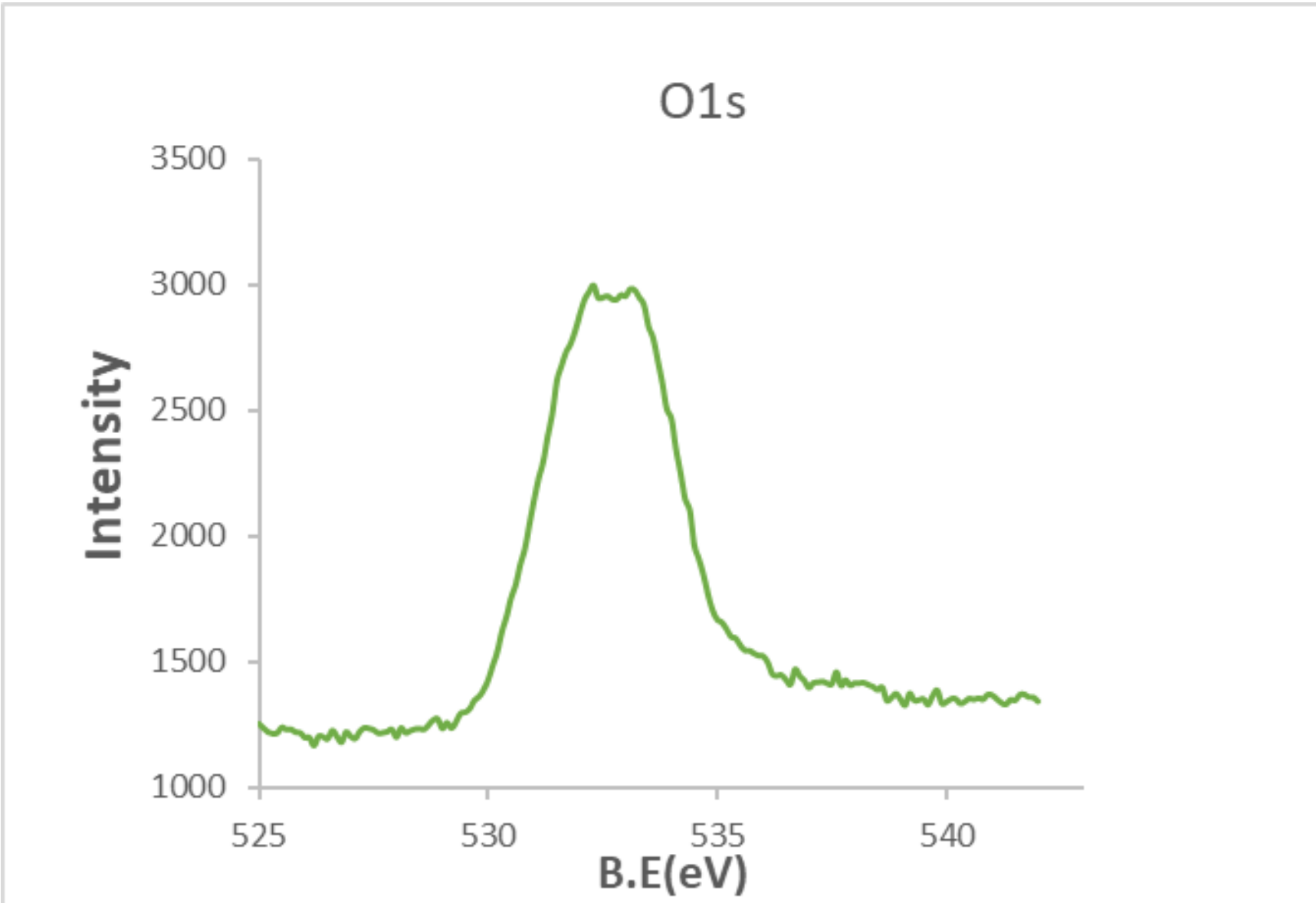
O1s
3500
3000
2500
2000
1500
1000
Intensity
525
530
535
540
B.E(eV)

d)

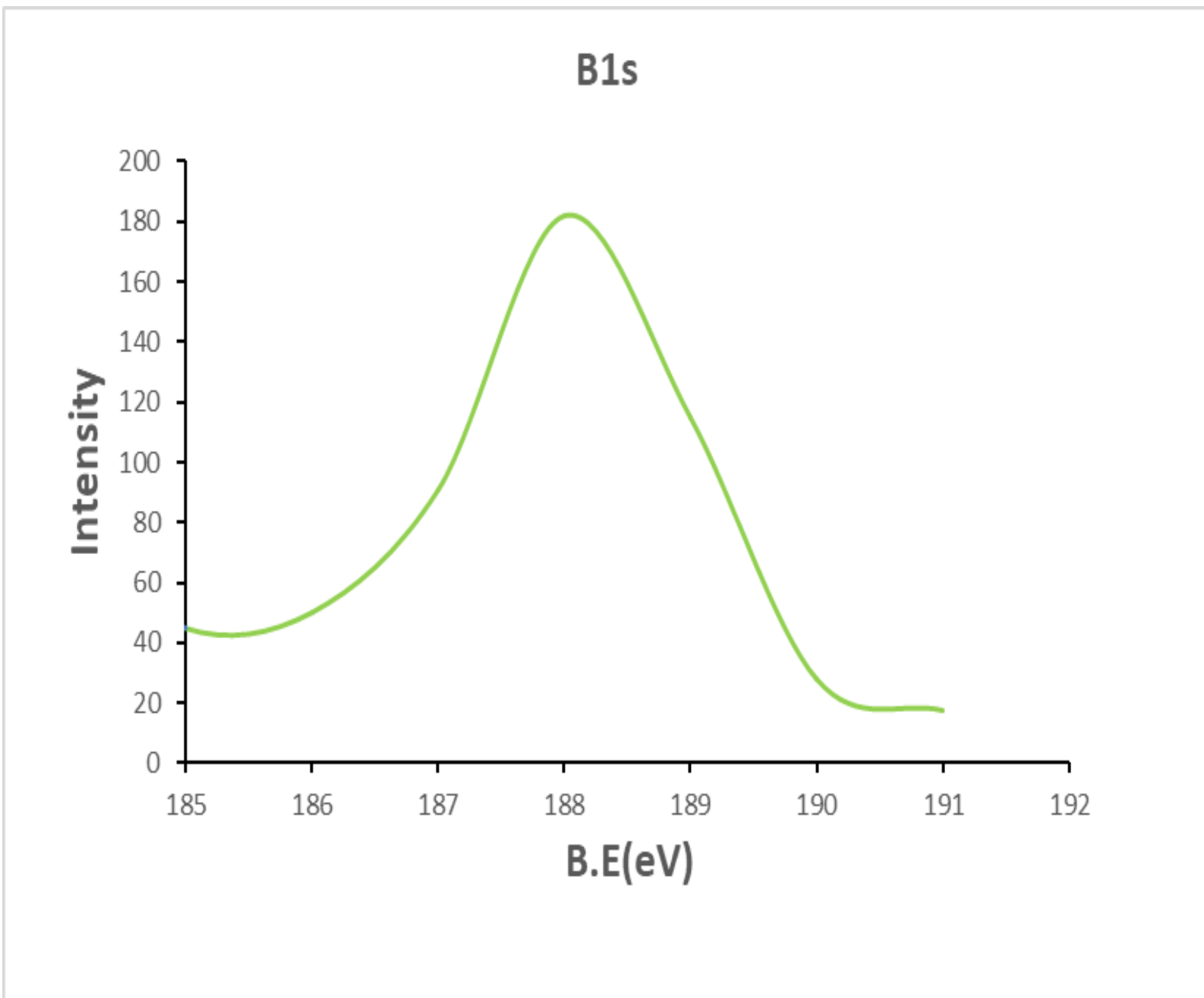
B1s
200
180
160
140
120
100
80
60
40
20
0
Intensity
185
186
187
188
189
190
191
192
B.E(eV)

Figure S16. DPVs for GO@3 in CH_3CN + 0.1 M [*n*-Bu_4N][PF_6] ($TBAPF_6$) *vs* SCE, showing the anodic (a) and cathodic (b) regions.

a)

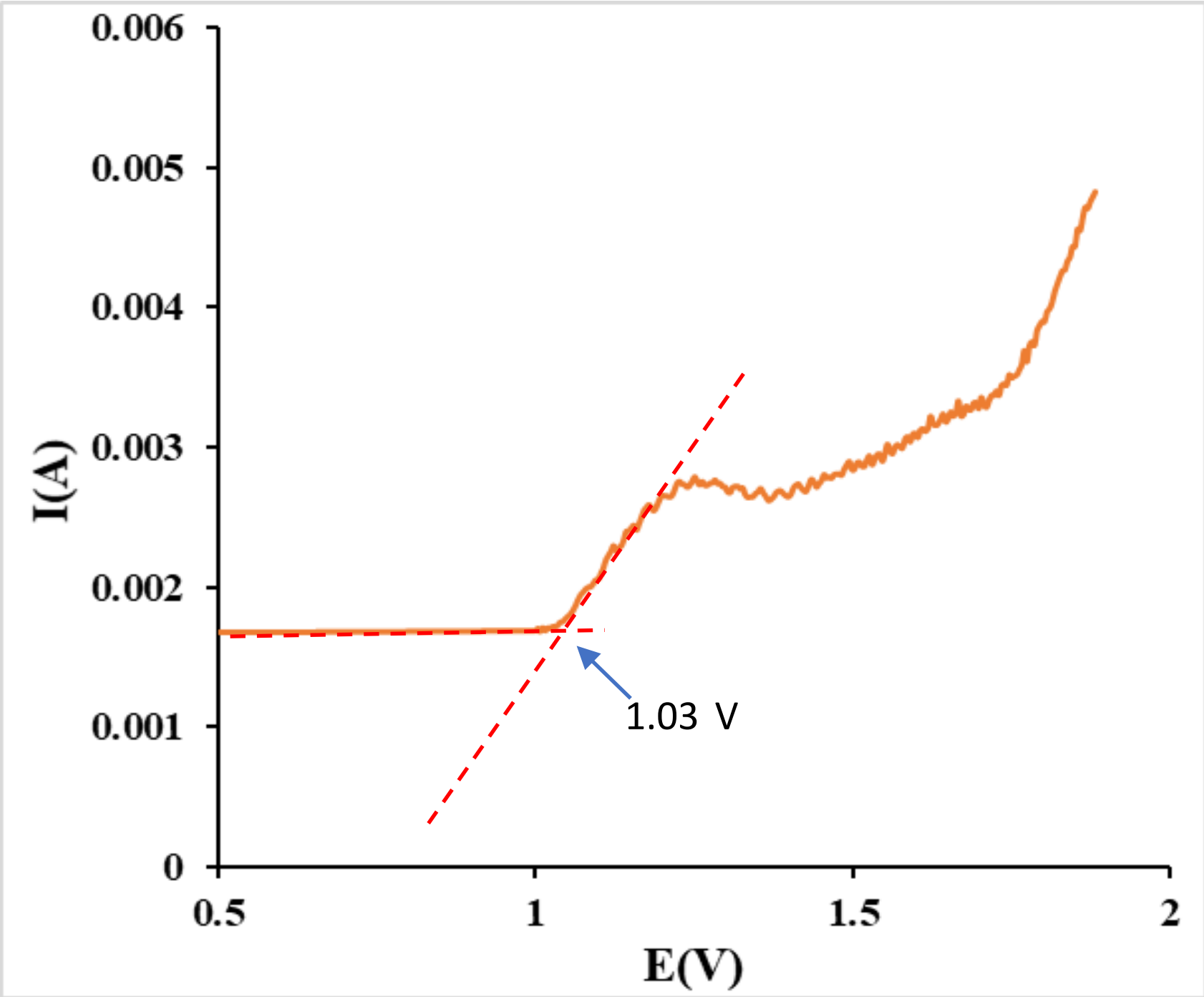

b)

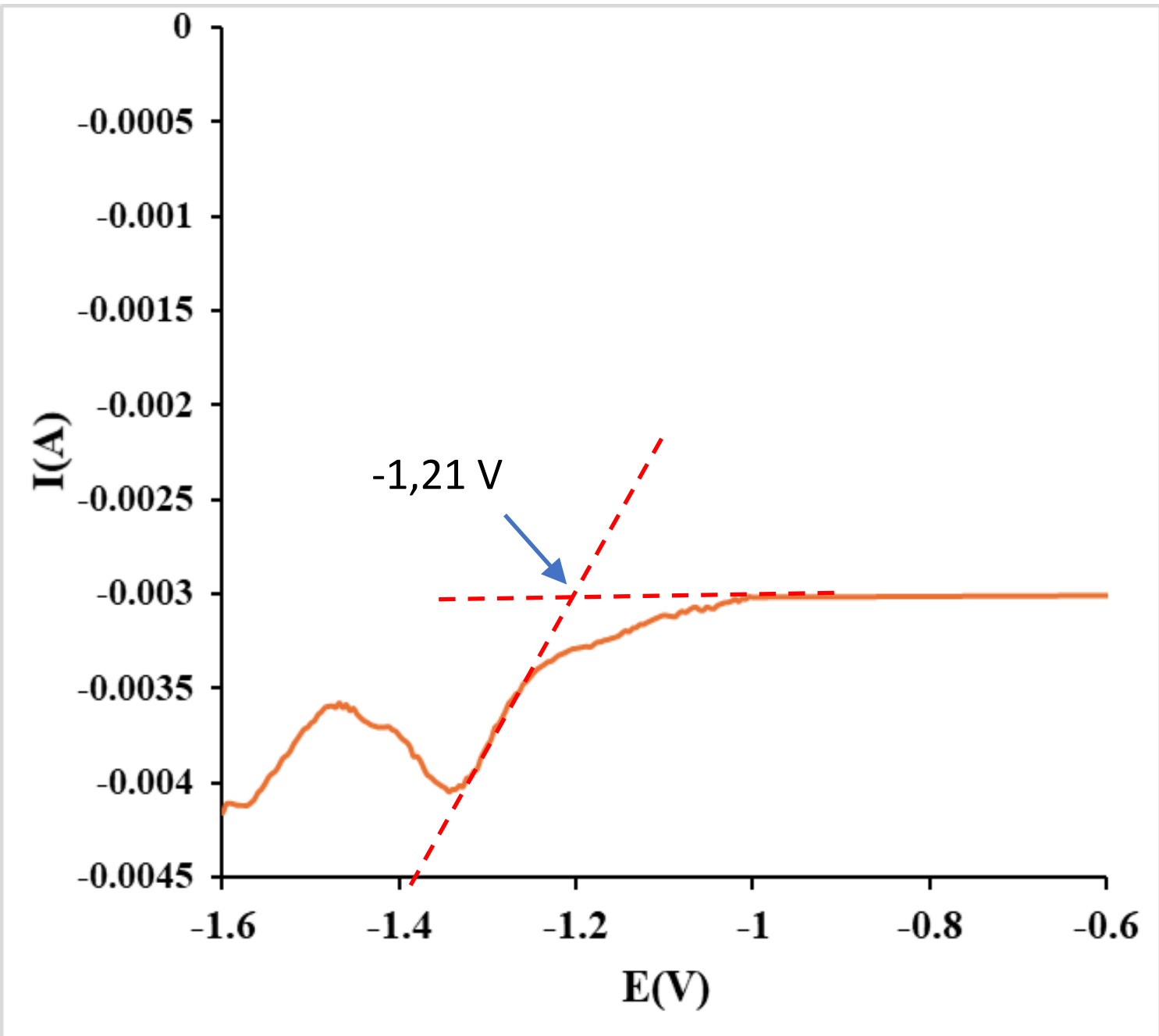

Figure S17. Tauc's plot to determine the optical bandgap of $[N(CH_3)_4][\mathbf{4}]$.

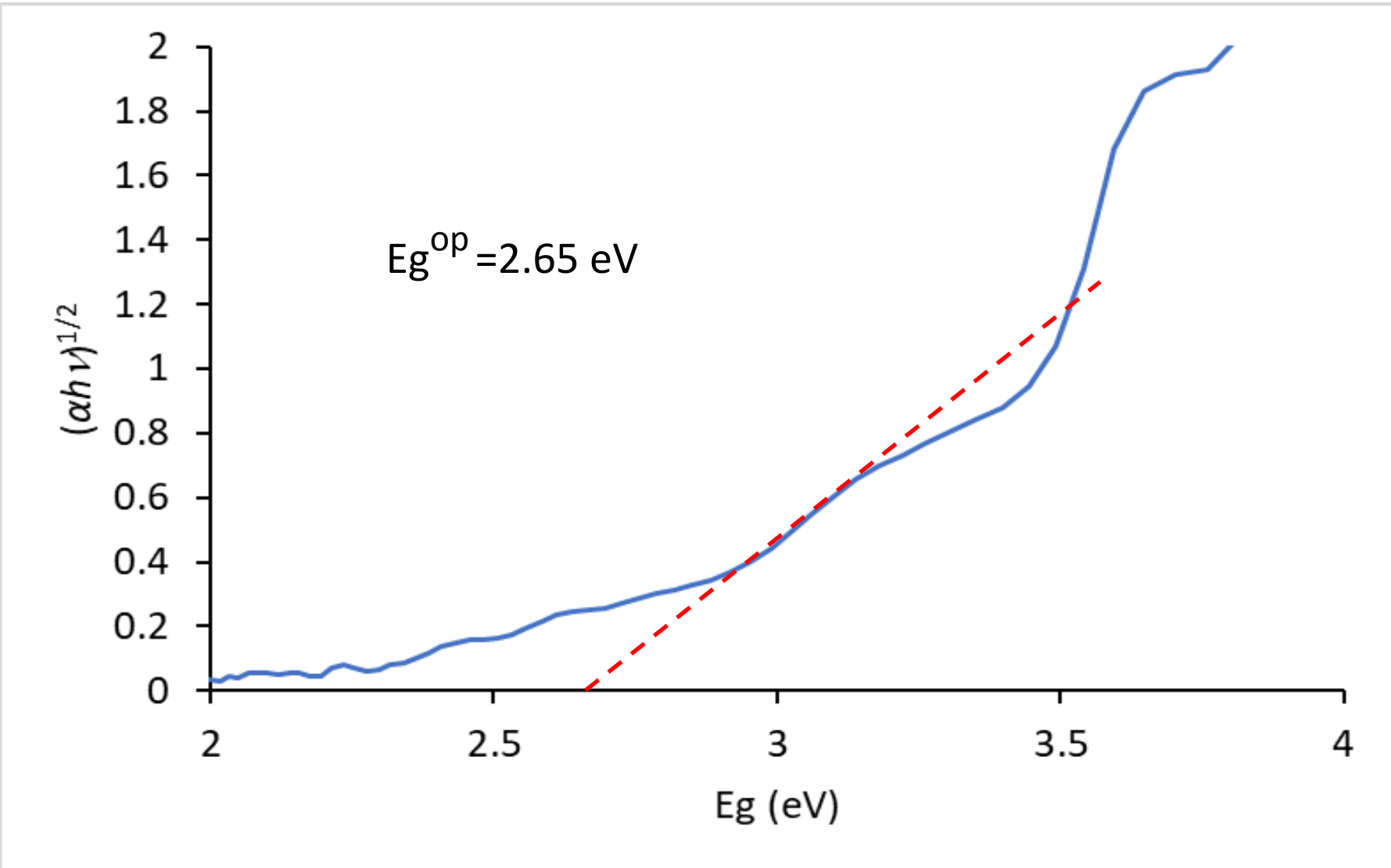

Figure S18. Plot of yield as a function of time for the photoredox catalysis of 1-phenylethanol. Conditions: Na[**3**] 0.02 mM (orange line) or 0.002 mM (blue line), substrate (20 mM), $Na_2S_2O_8$ (40 mM), 5 mL water at pH=7, light irradiation (λ= 352 nm).

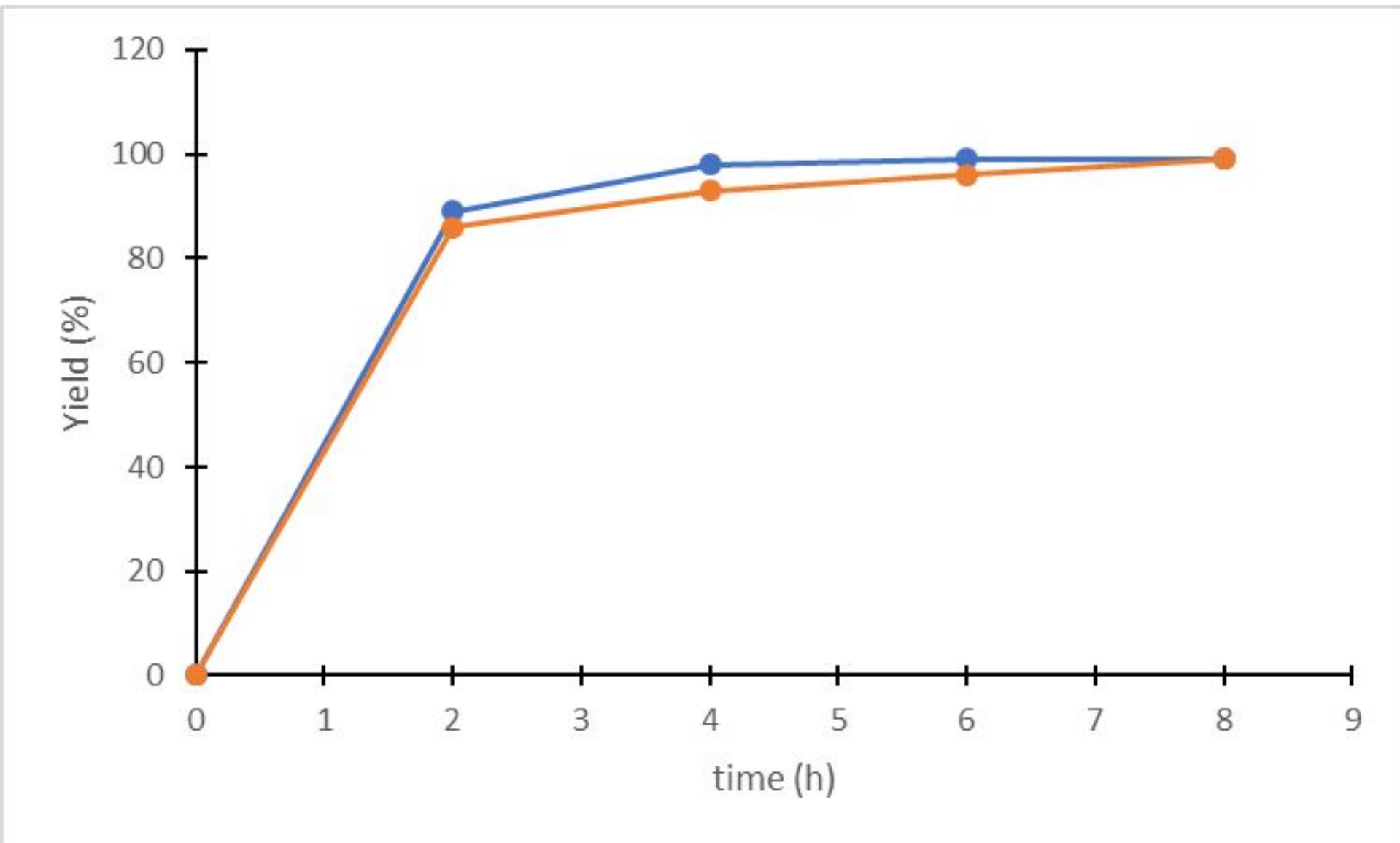

Figure S19. Yield values obtained throughout a number of successive reuses of the catalyst $[N(CH_3)_4]$[**4**] in the photooxidation of 1-phenylethanol. Conditions: $[N(CH_3)_4]$[**4**] (1.3 µmol), substrate (0.26 M), $Na_2S_2O_8$ (0.53 M), 5 mL water at pH=7, light irradiation using a lamp with λ = 352 nm, 6 h per run.

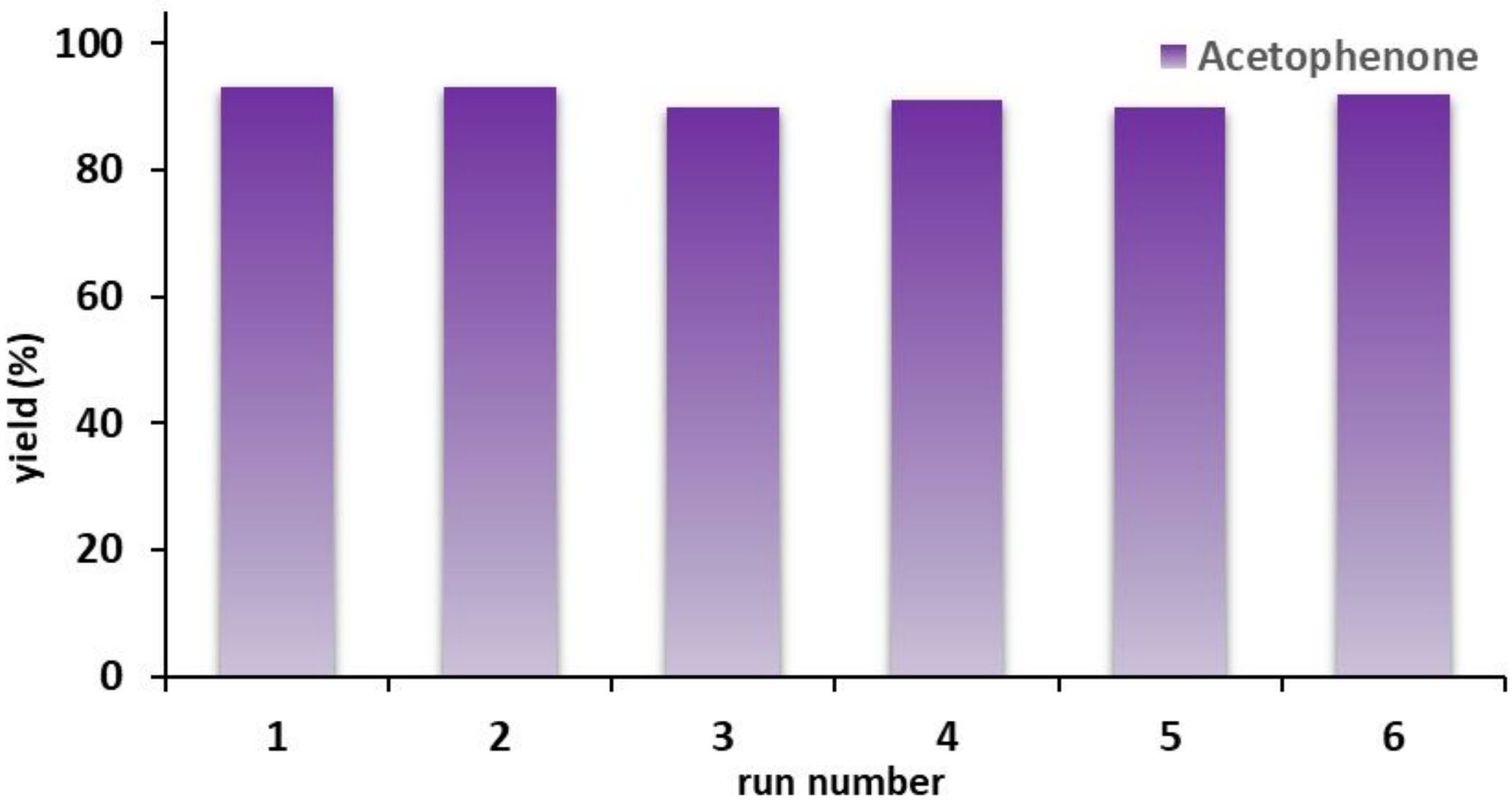

Figure S20. TEM images of GO@**3** after six reuses, (the bars indicate a length of a) 100 nm; b) 50 nm).

a)

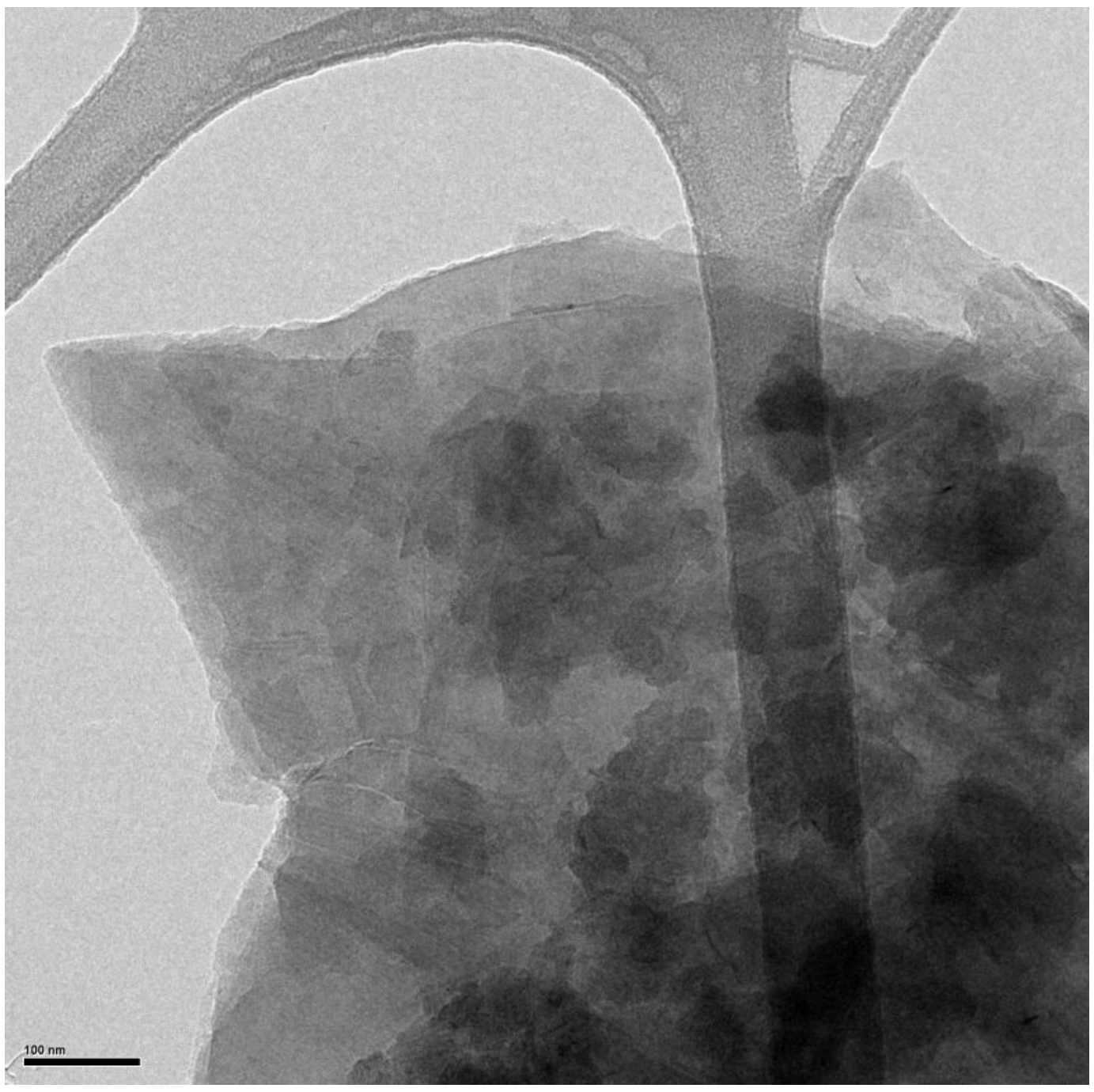

b)

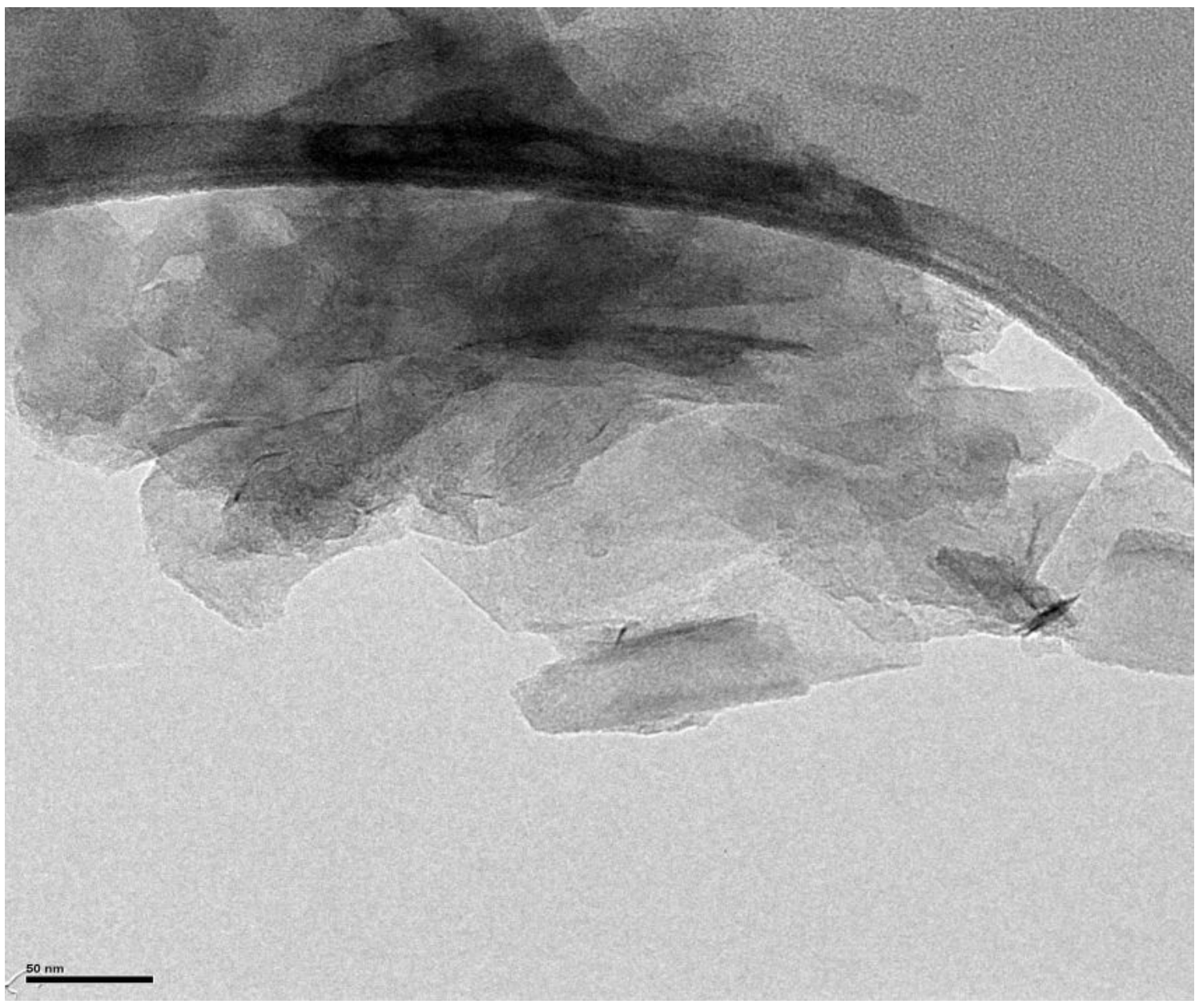

Figure S21. Representative NMR spectra of the obtained products in the photooxidation of alcohols.

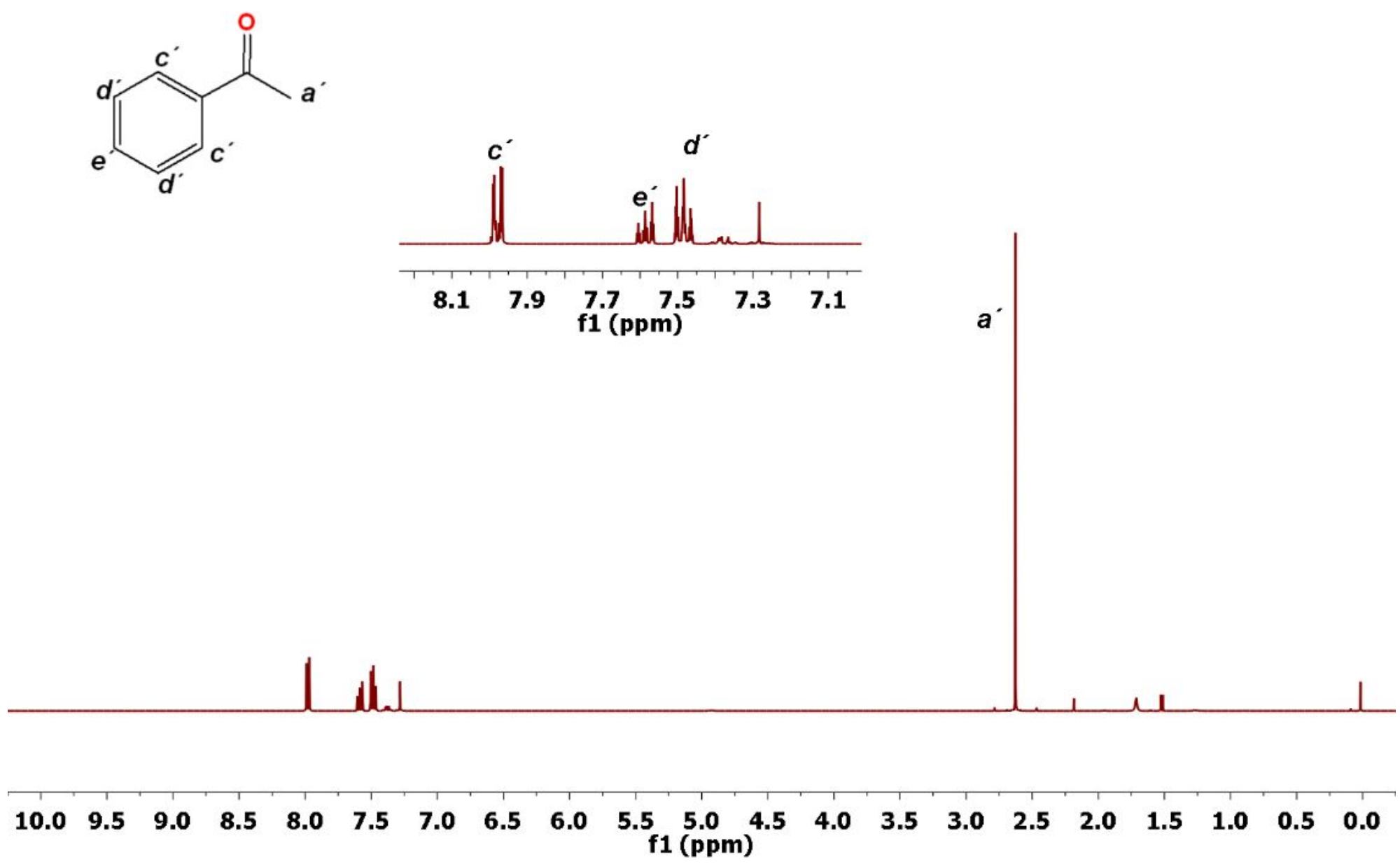

^{1}H NMR spectrum ($CDCl_3$) corresponding to the photooxidation of 1-phenylethanol, using **GO@3** as photocatalyst. Results showed in ***Table 2, entry 1b.***

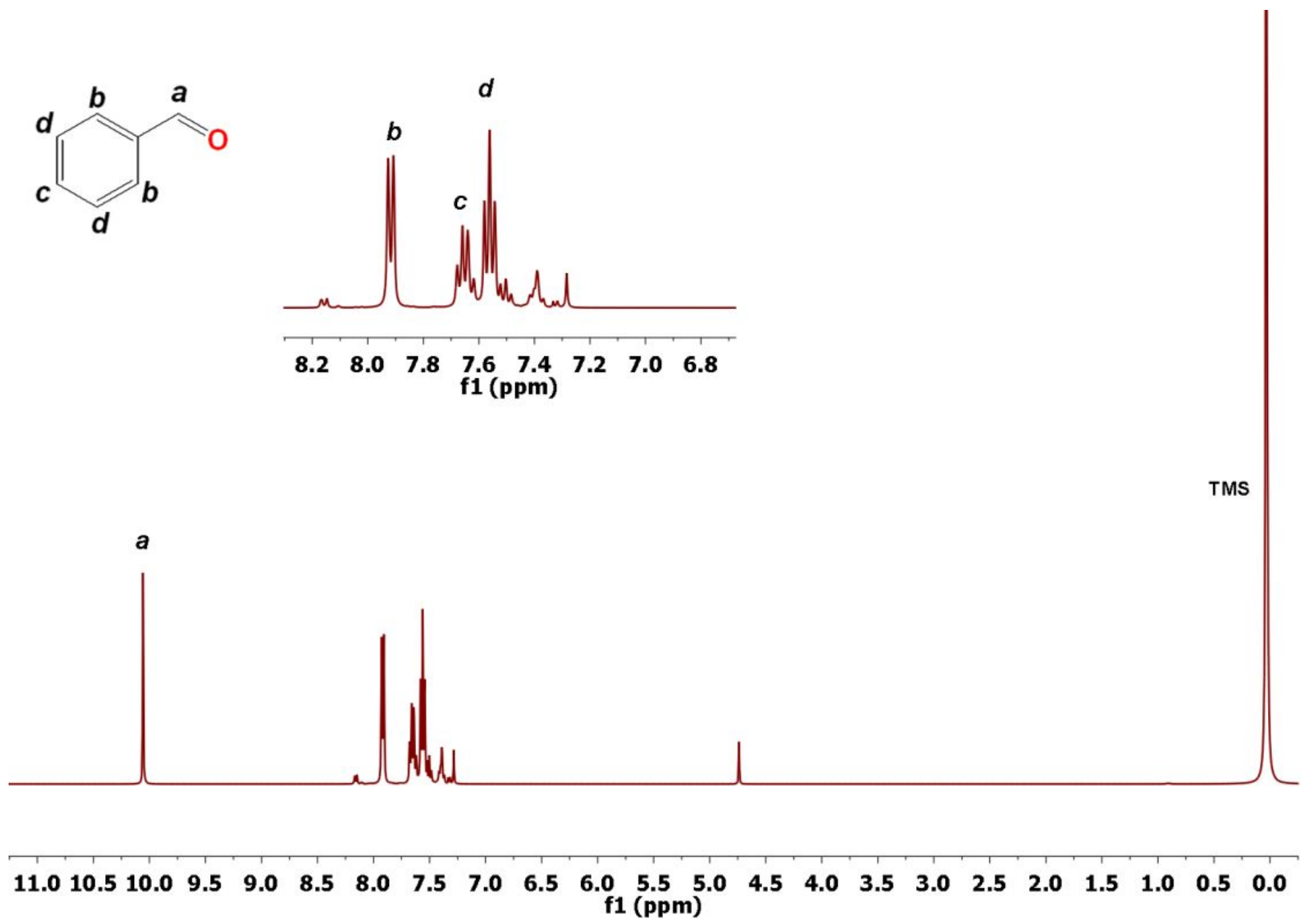

^{1}H NMR spectrum ($CDCl_3$) corresponding to the photooxidation of benzyl alcohol, using **GO@3** as photocatalyst. Results showed in ***Table 2, entry 2b.***

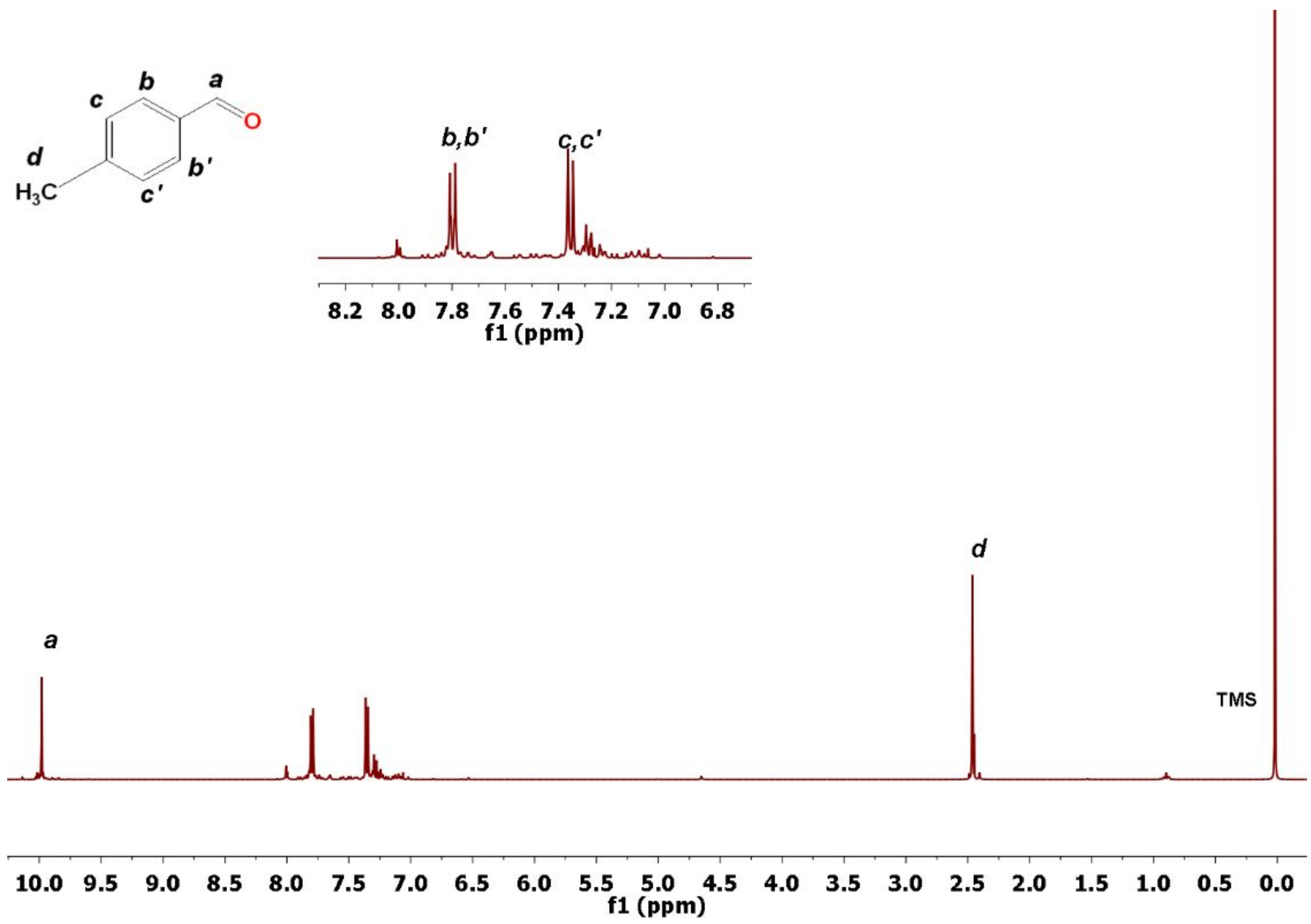

^{1}H NMR spectrum ($CDCl_3$) corresponding to the photooxidation of 4-methyl benzyl alcohol, using **GO@3** as photocatalyst. Results showed in ***Table 2, entry 3b.***

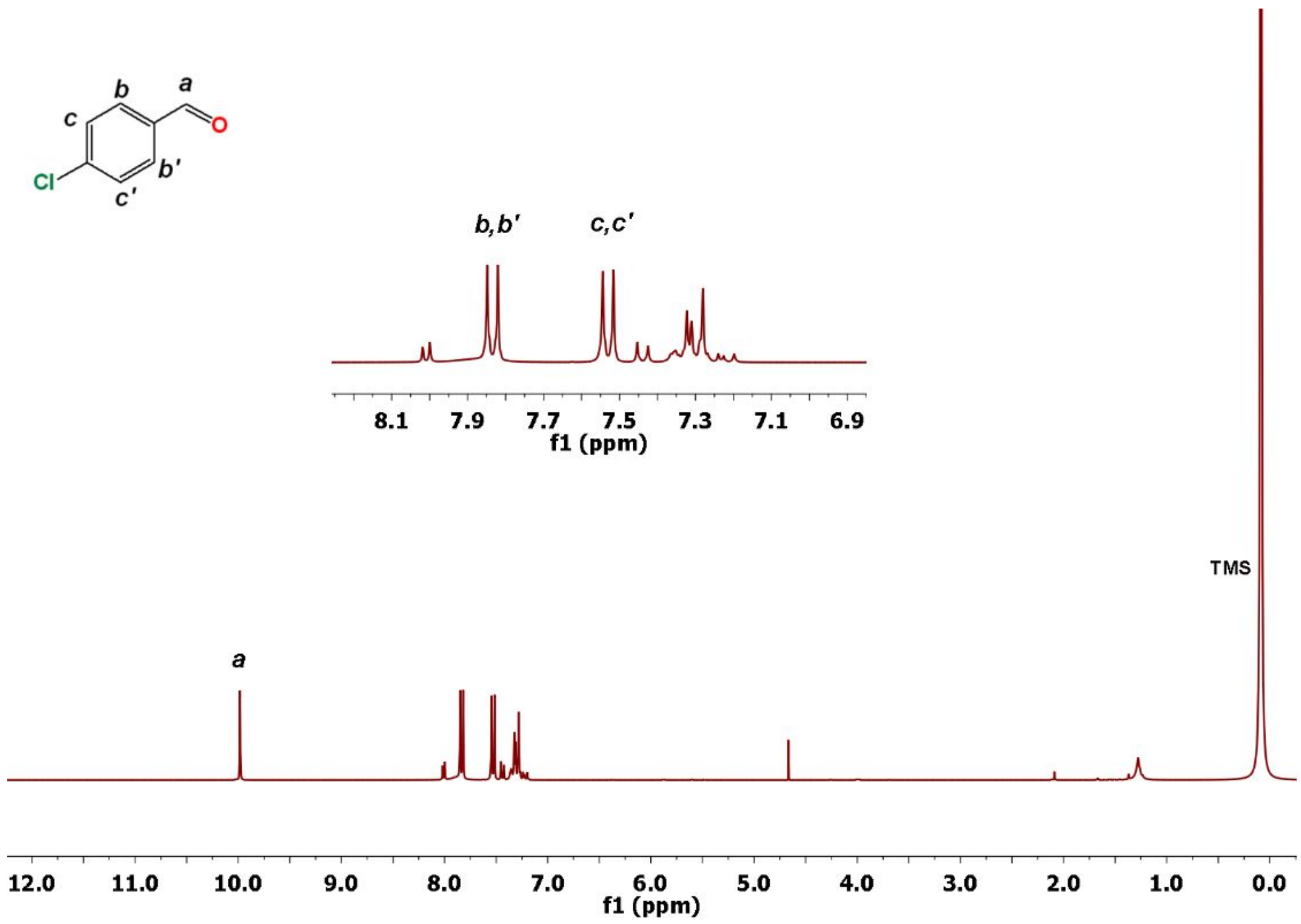

^{1}H NMR spectrum ($CDCl_3$) corresponding to the photooxidation of 4-chloro benzyl alcohol, using **GO@3** as photocatalyst. Results showed in ***Table 2, entry 4b.***

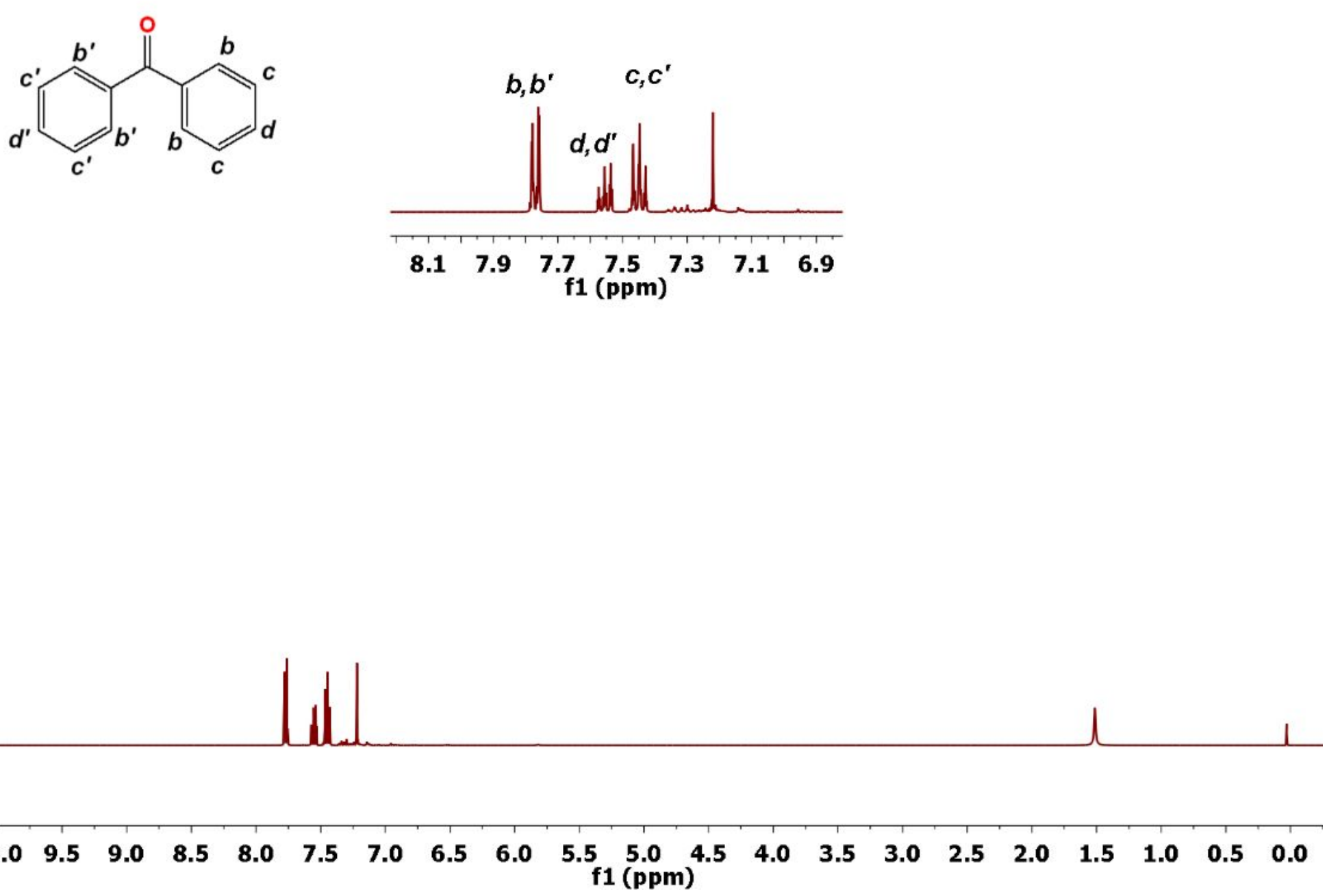

^{1}H NMR spectrum ($CDCl_3$) corresponding to the photooxidation of diphenyl methanol, using **GO@3** as photocatalyst. Results showed in ***Table 2, entry 5b*.**

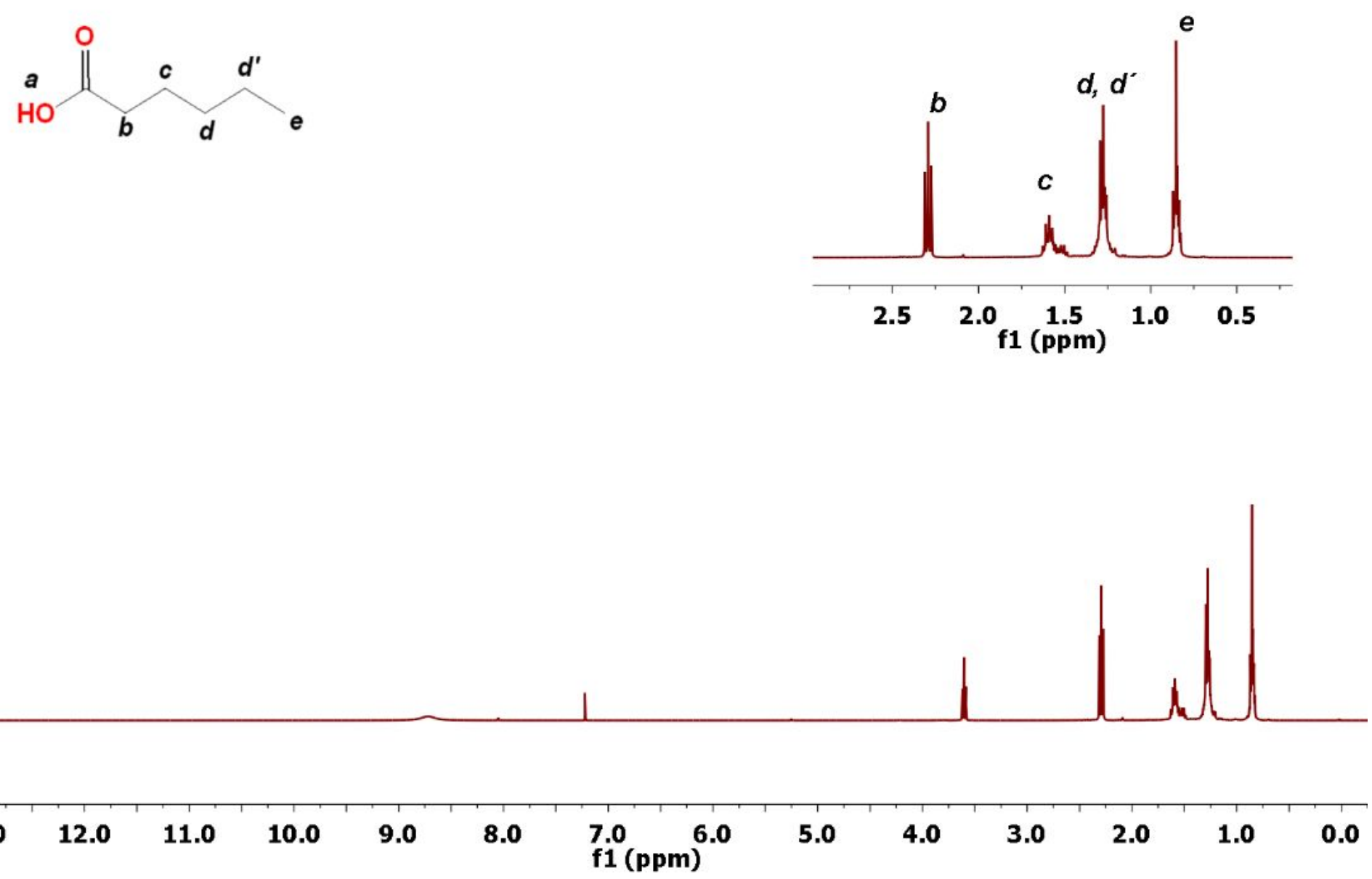

^{1}H NMR spectrum ($CDCl_3$) corresponding to the photooxidation of 1-hexanol, using **GO@3** as photocatalyst. Results showed in ***Table 2, entry 6b*.**

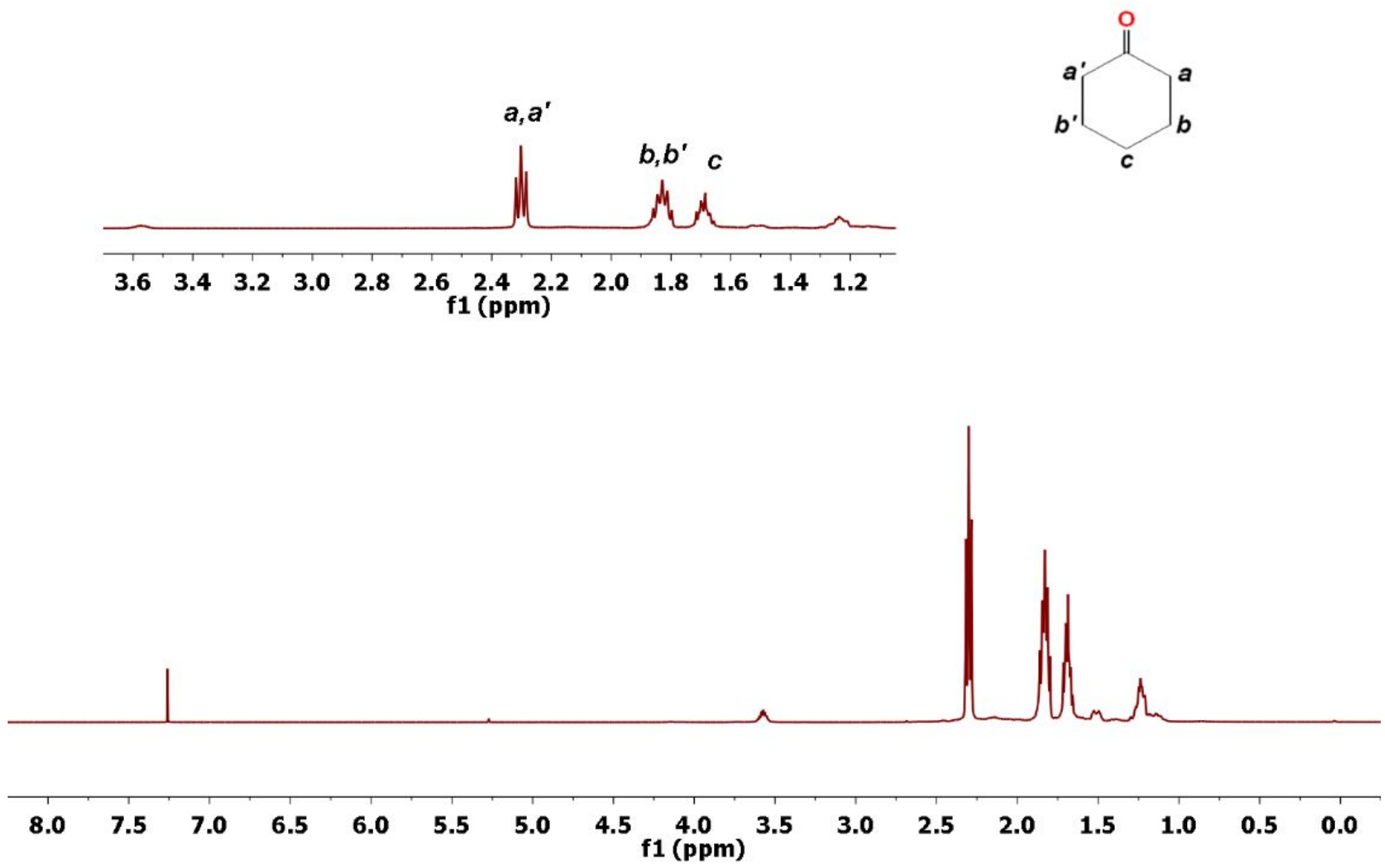

^{1}H NMR spectrum ($CDCl_3$) corresponding to the photooxidation of cyclohexanol, using **GO@3** as photocatalyst. Results showed in ***Table 2, entry 7b.***

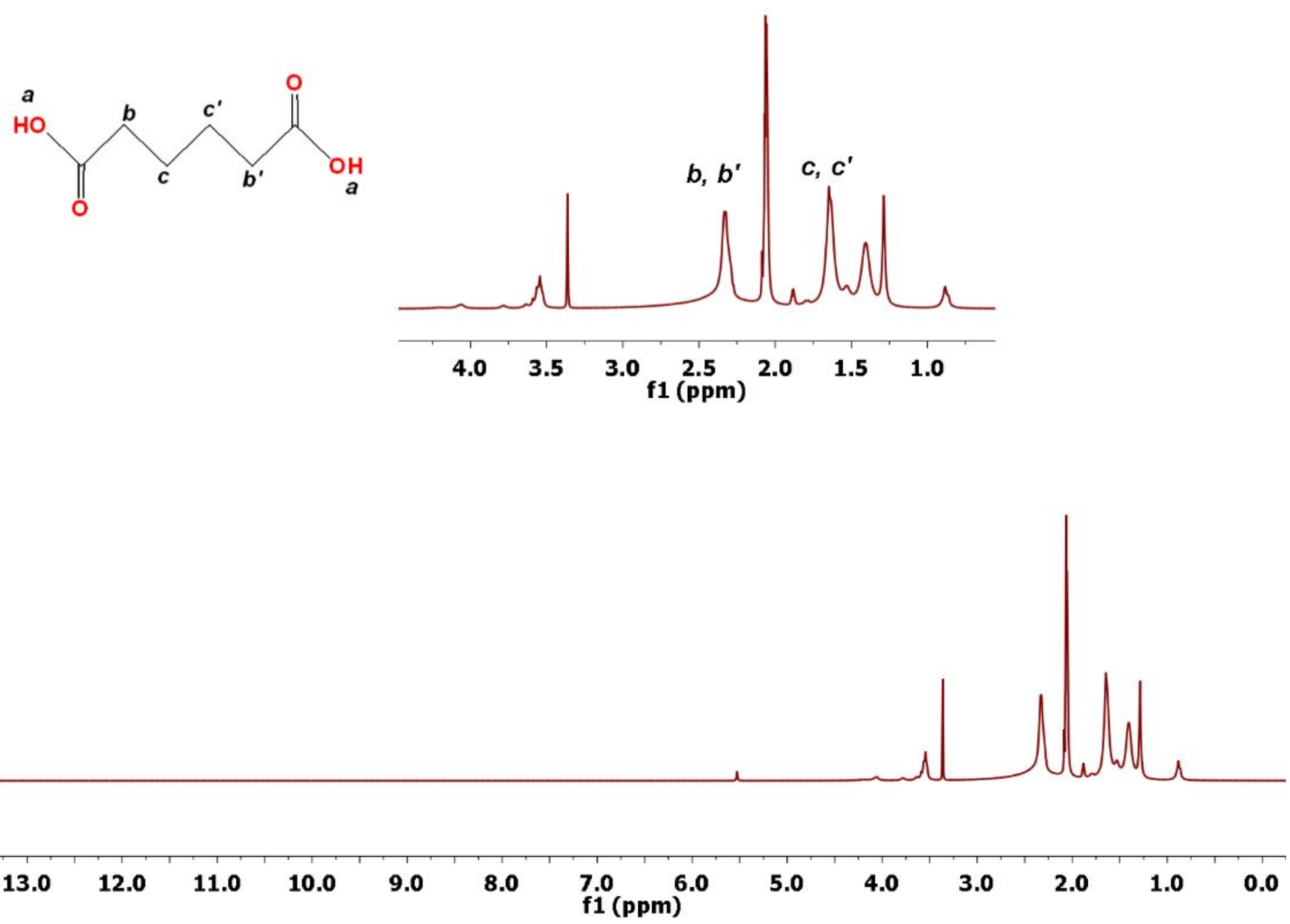

^{1}H NMR spectrum (acetone-d_6) corresponding to the photooxidation of 1,6-hexanediol, using **GO@3** as photocatalyst. Results showed in ***Table 2, entry 8b.***

Figure S22. NMR spectra of the pure products in the photooxidation of alcohols.

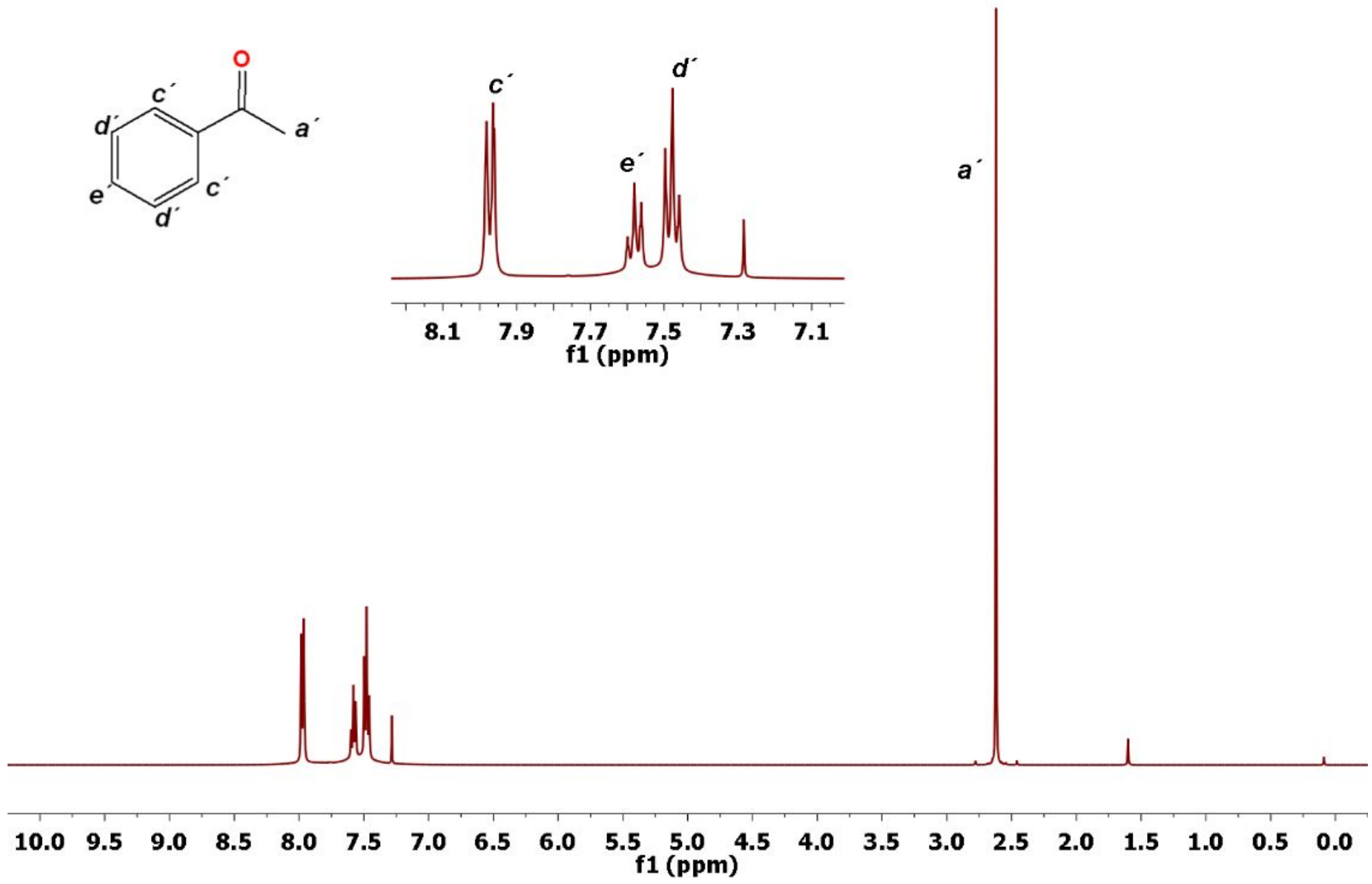

^{1}H NMR spectrum ($CDCl_3$) corresponding to pure acetophenone

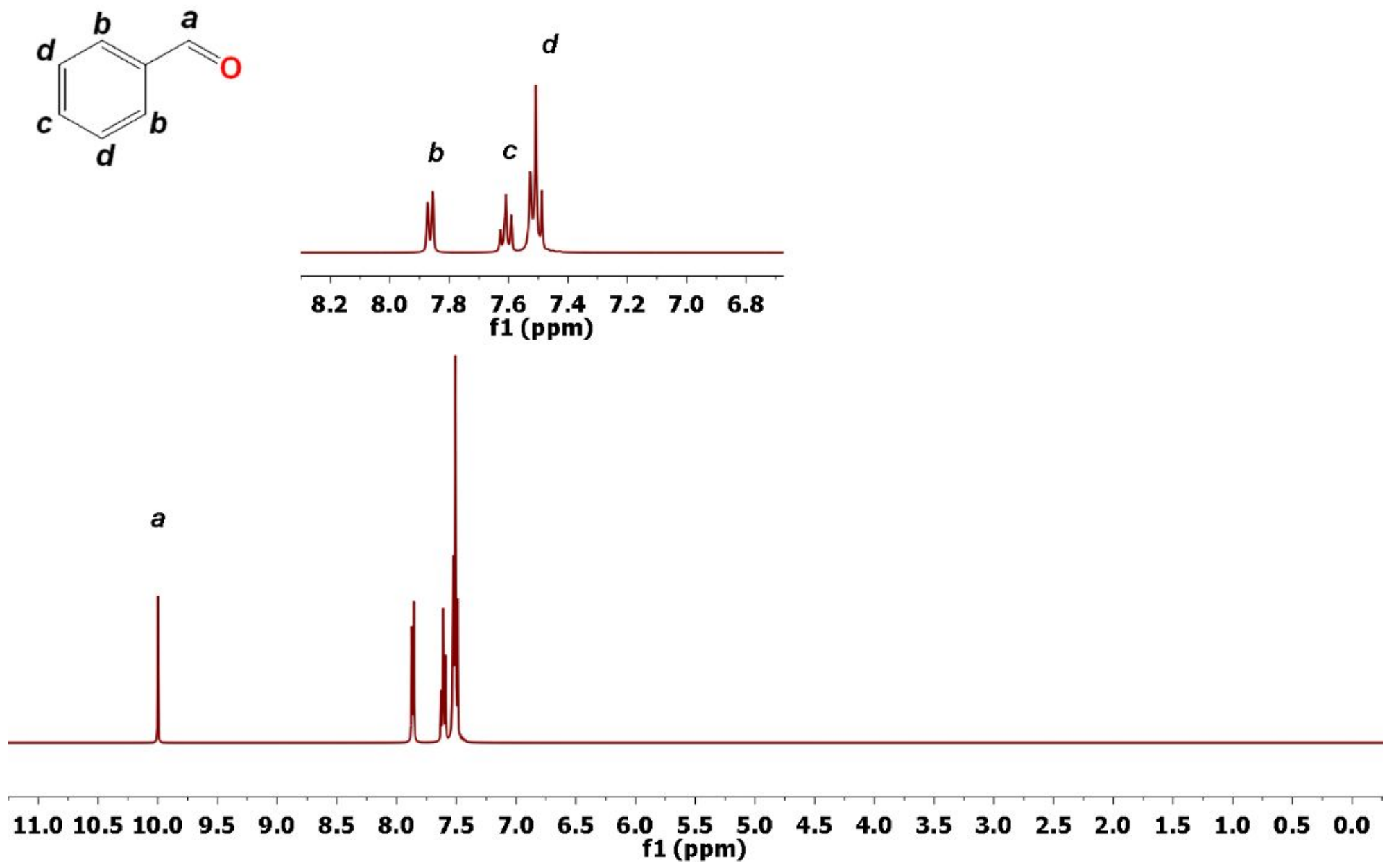

^{1}H NMR spectrum ($CDCl_3$) corresponding to pure benzaldehyde

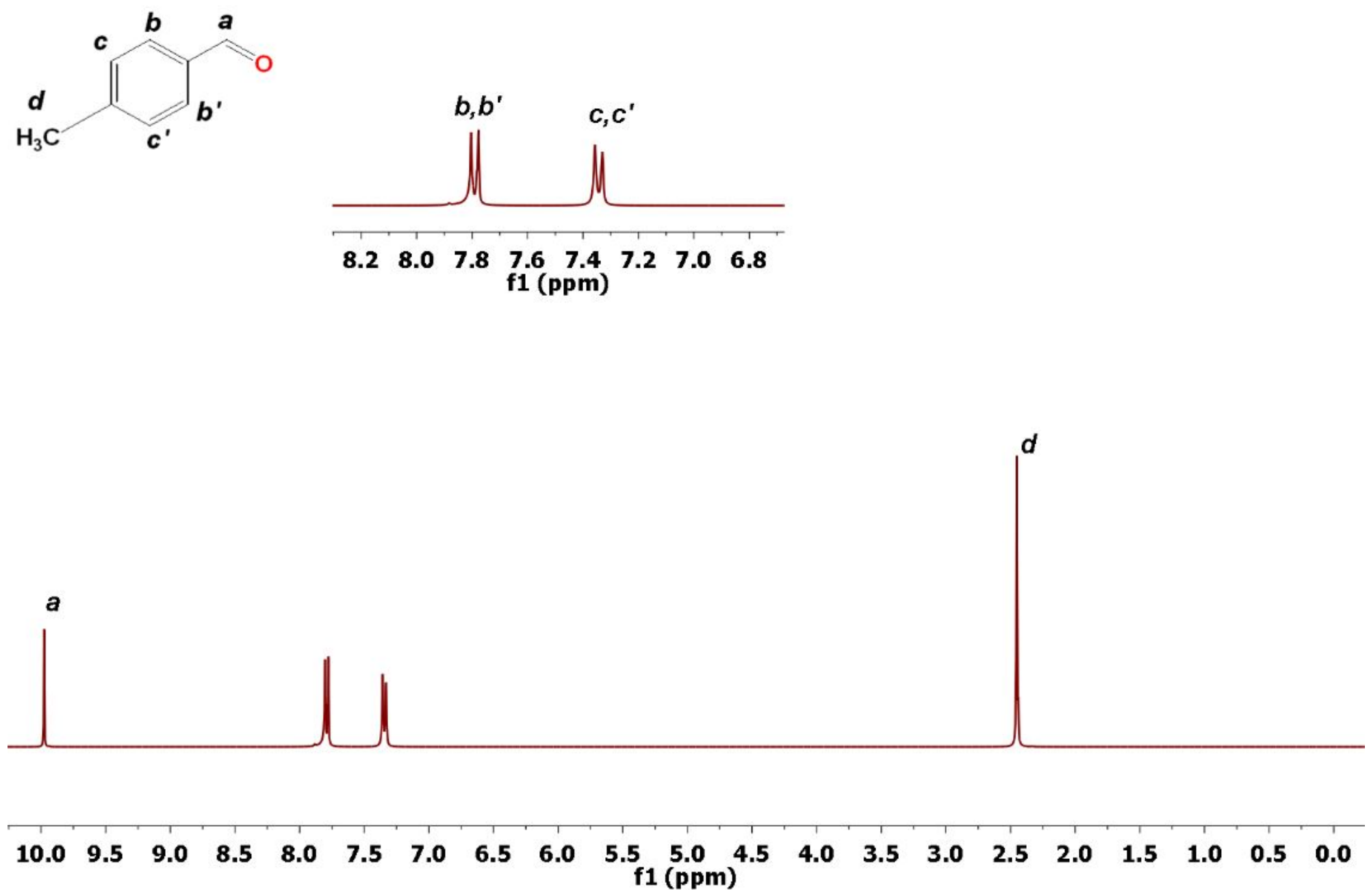

^{1}H NMR spectrum ($CDCl_3$) corresponding to pure *p*-toluenbenzaldehyde

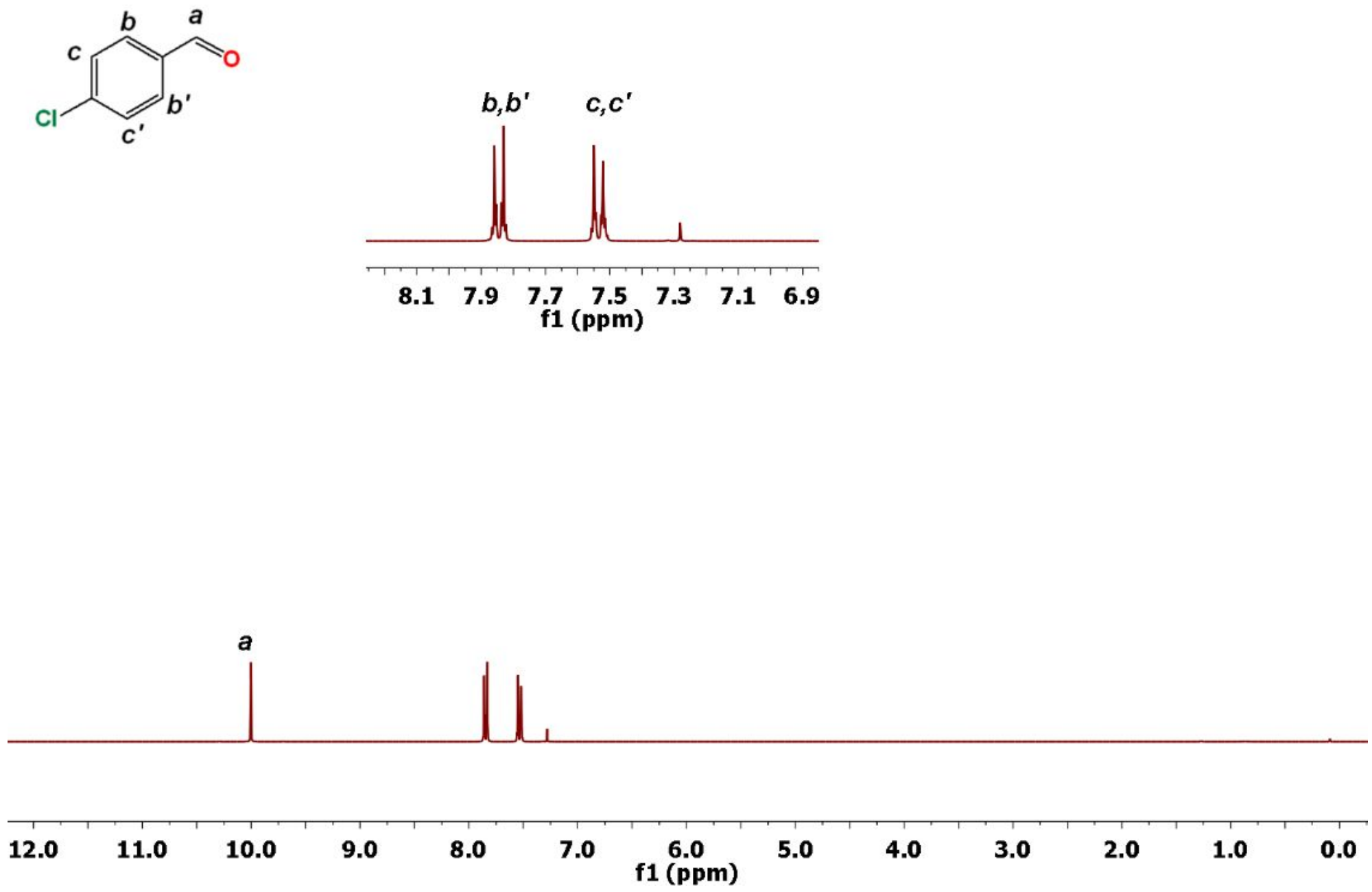

^{1}H NMR spectrum ($CDCl_3$) corresponding to pure 4-chloro benzaldehyde

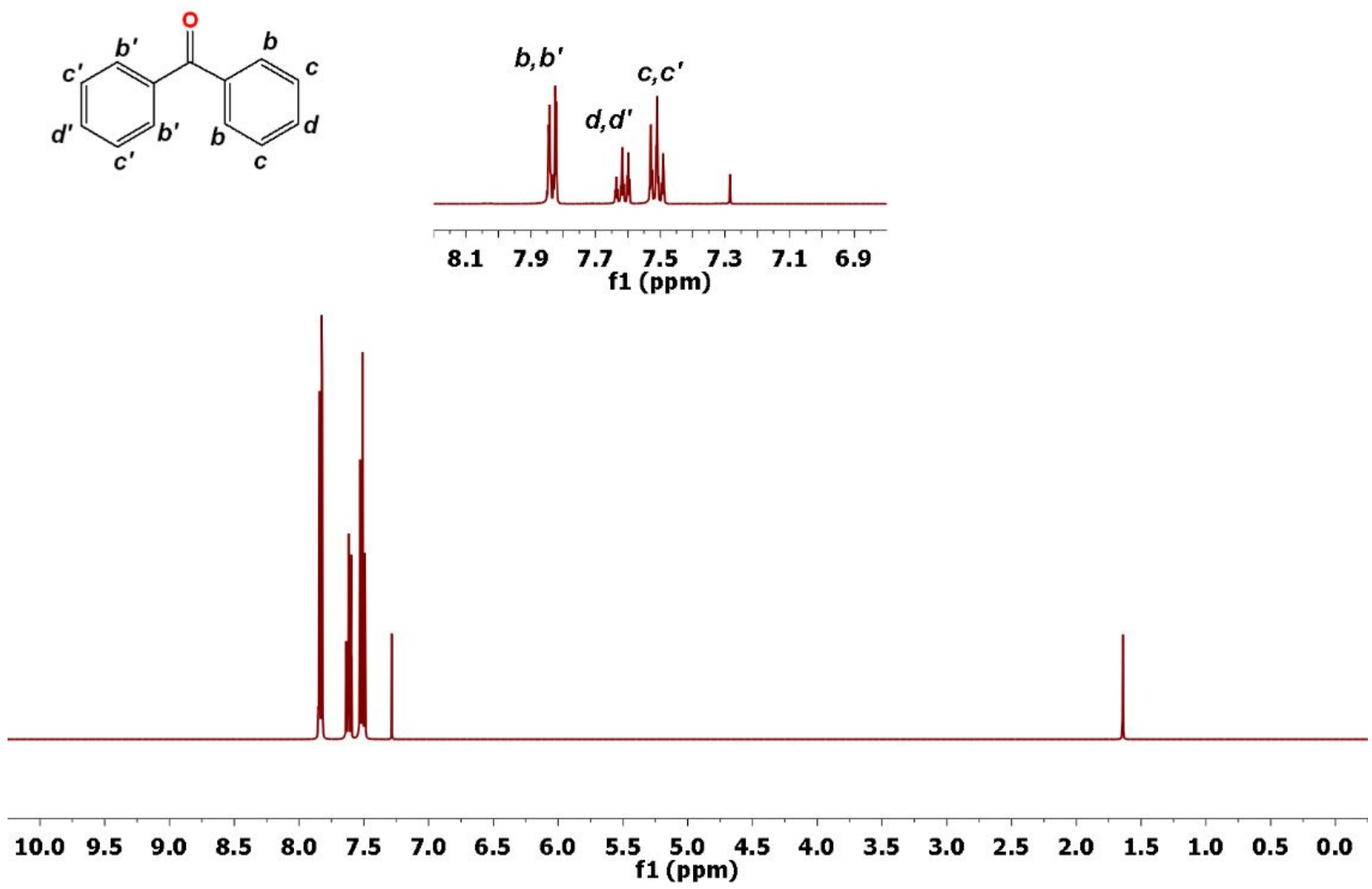

^1H NMR spectrum ($CDCl_3$) corresponding to pure benzophenone

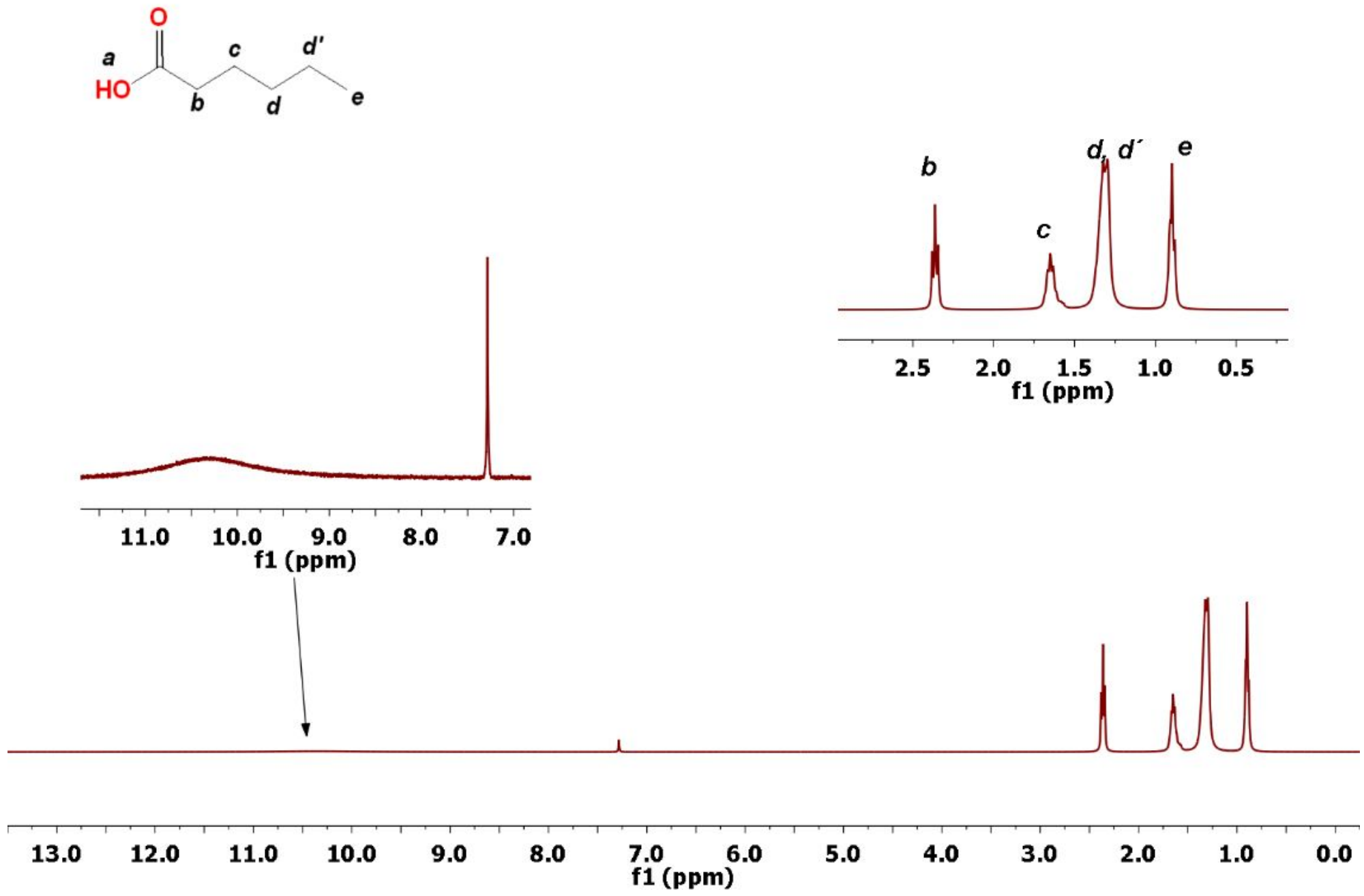

^1H NMR spectrum ($CDCl_3$) corresponding to pure hexanoic acid

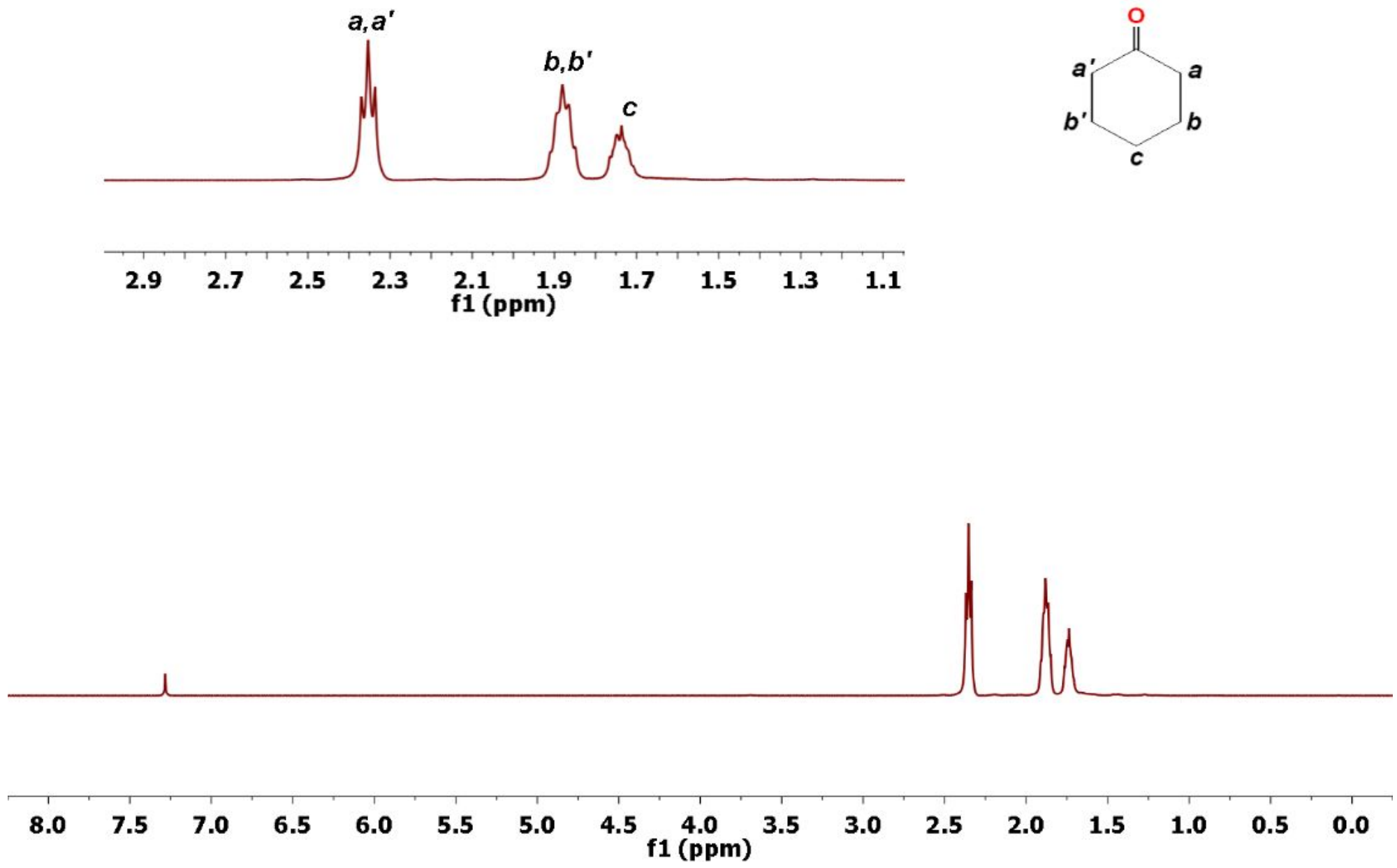

^{1}H NMR spectrum ($CDCl_3$) corresponding to pure cyclohexanone

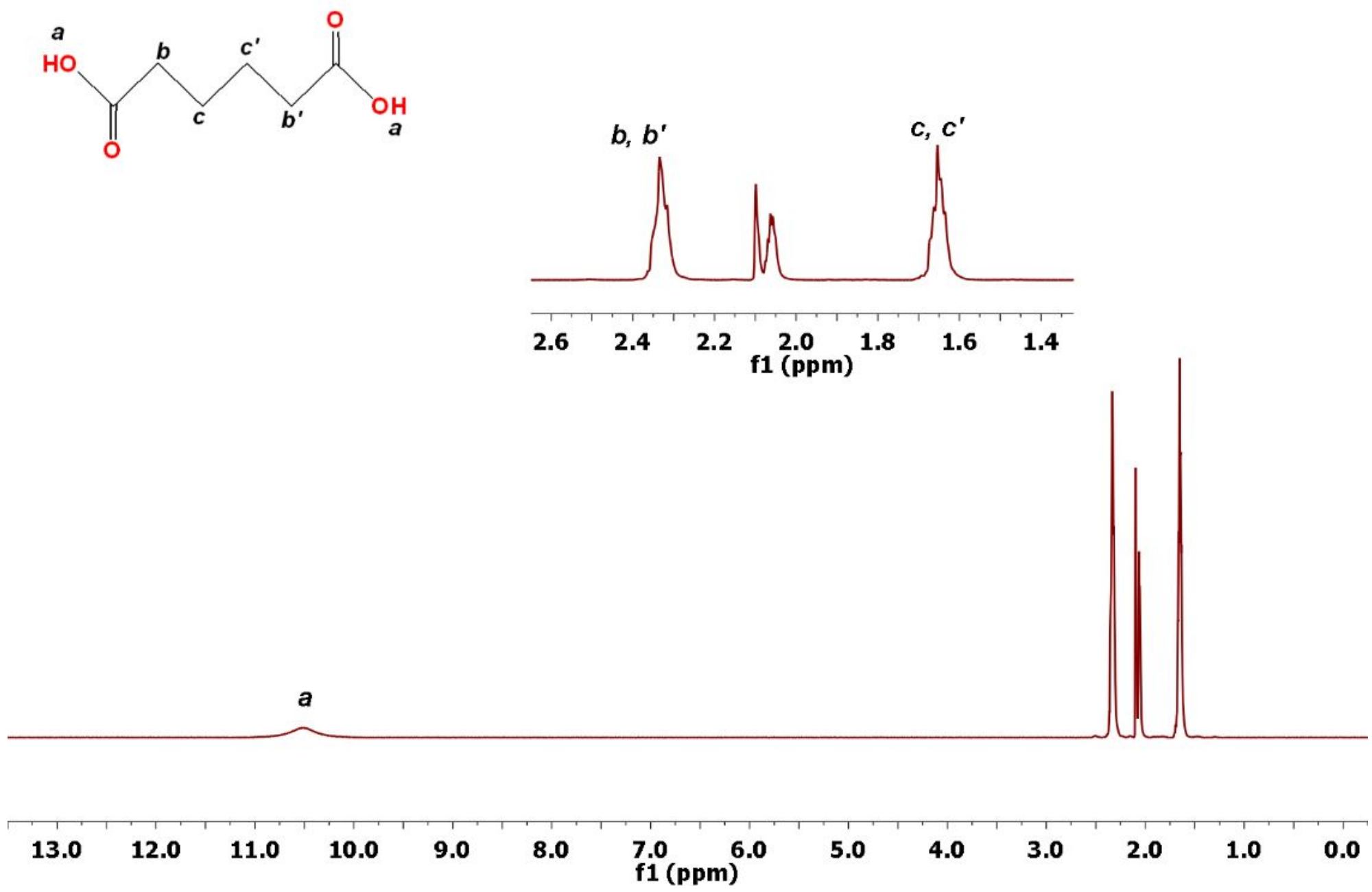

^{1}H NMR spectrum (acetone-d_6) corresponding to pure adipic acid
